# Natural and Anthropogenic Sources of Seismic, Hydroacoustic, and Infrasonic Waves: Waveforms and Spectral Characteristics (and Their Applicability for Sensor Calibration)

**DOI:** 10.1007/s10712-022-09713-4

**Published:** 2022-07-25

**Authors:** Michaela Schwardt, Christoph Pilger, Peter Gaebler, Patrick Hupe, Lars Ceranna

**Affiliations:** Federal Institute for Geosciences and Natural Resources, Stilleweg 2, 30655 Hannover, Germany

**Keywords:** Comprehensive nuclear-test-ban treaty, International monitoring system, Metrology, Calibration, Waveforms, Natural/man-made signals

## Abstract

The record of seismic, hydroacoustic, and infrasonic waves is essential to detect, identify, and localize sources of both natural and anthropogenic origin. To guarantee traceability and inter-station comparability, as well as an estimation of the measurement uncertainties leading to a better monitoring of natural disasters and environmental aspects, suitable measurement standards and reliable calibration procedures of sensors, especially in the low-frequency range down to 0.01 Hz, are required. Most of all with regard to the design goal of the Comprehensive Nuclear-Test-Ban Treaty Organisation’s International Monitoring System, which requires the stations to be operational nearly 100% of the time, the on-site calibration during operation is of special importance. The purpose of this paper is to identify suitable excitation sources and elaborate necessary requirements for on-site calibrations. We give an extensive literature review of a large variety of anthropogenic and natural sources of seismic, hydroacoustic, and infrasonic waves, describe their most prominent features regarding signal and spectral characteristics, explicitly highlight some source examples, and evaluate the reviewed sources with respect to requirements for on-site calibrations such as frequency bandwidth, signal properties as well as the applicability in terms of cost–benefit. According to our assessment, earthquakes stand out across all three waveform technologies as a good natural excitation signal meeting the majority of the requirements. Furthermore, microseisms and microbaroms allow a calibration at very low frequencies. We also find that in each waveform technique man-made controlled sources such as drop weights or air guns are in good agreement with the required properties, although limitations may arise regarding the practicability. Using these sources, procedures will be established allowing calibration without record interrupting, thereby improving data quality and the identification of treaty-related events.

## Article Highlights

- Various sources of seismic, hydroacoustic, and infrasonic waves are analysed regarding their waveform and frequency content
- We evaluate the potential use of both natural and anthropogenic sources as excitation signals for on-site calibration
- Earthquakes and man-made signals are in best agreement with the required properties for excitation signals

## Introduction

Seismic, hydroacoustic, and infrasonic (SHI) waves are emitted by a great variety of sources of both natural and anthropogenic origin. It is essential to detect and record these waves in order to monitor extreme events such as earthquakes, volcanic eruptions, or nuclear explosions. As the record of such waves has a long history, the monitoring technologies in this field are well established: Local and global networks of seismic, hydroacoustic, and infrasonic sensors such as the German Regional Seismic Network (GRSN; e.g. Stammler et al. 2021) or the Global Seismographic Network (GNS; e.g. Gee and Leith 2011) record the waveform data and make it available to a wide scientific community. Additionally, measurements of infrasonic, hydroacoustic, and seismic signals are core technologies within the International Monitoring System (IMS) for compliance with the Comprehensive Nuclear-Test-Ban Treaty (CTBT). However, the data quality is affected by the lack of metrological measurement standards as well as on-site calibration procedures in the low-frequency range down to 0.1 Hz and lower, which establish a relation between the measured electrical signal by the sensor and the respective unit of physical quantity. Yet, calibration at periodic intervals is necessary for maintaining traceability to the International System of Units (SI). As laboratory calibration requires dismantling of the sensors as well as high efforts, on-site calibration procedures using comparison calibration with traceable transfer standards are considered. Concerning the safety–critical design goal of the IMS, which requires the stations to be operational nearly 100% of the time, the on-site calibration during operation is of special importance as it neither interrupts the measurements, nor changes are made to certified sensors. There are several technical requirements for IMS stations, e.g. “Mission capability” or “Data availability”, that need to be fulfilled and are influenced by the number of operational sensors. Although there are some standards for the calibration of vibration transducers (e.g. ISO 16063-1; ISO 16063-11; ISO 16063-12; ISO 16063-21; see Veldman 2006 for an overview), they do not cover the whole frequency range of seismometers, especially the low-frequency range is excluded so far. As part of the joint research project “Metrology for low-frequency sound and vibration—19ENV03 Infra-AUV” primary and secondary calibration methods are developed and suitable standards are selected to provide traceable calibration for environmental sensors deployed in monitoring networks as well as define the influences of measurement uncertainties. Within the project and this paper, we review a variety of human-made as well as natural sources of seismic, hydroacoustic, and infrasonic waves and evaluate them in order to identify suitable excitation sources for on-site calibrations.

From a physical point of view, SHI waves are mechanical waves, which are particle deformations that propagate through a medium by generating local stress and strain within that medium, and transporting energy. In general, waves are characterized by frequency, velocity, and amplitude. Further waveform, signal length, onset time, and polarity, which all depend on the wave type, the source mechanism and duration as well as the path through the propagation medium are important parameters to consider when describing SHI waves.

A seismic wave is an oscillation caused by elastic deformation (Bormann et al. 2013). Based on their propagation, seismic waves are divided into body waves, propagating through the Earth’s interior, and surface waves, propagating along the Earth’s surface. Body waves are further subdivided according to their type of particle motion into compressional P waves, and S waves, which are shear waves. For the surface waves, a distinction is made between Rayleigh, Love, and Stoneley waves. Hydroacoustic and infrasonic waves are sound waves propagating through either water or the atmosphere, respectively. Generally, sound waves are longitudinal waves in which the particle motion occurs in the same direction as propagation. While propagating through a medium, a sound wave disturbs the equilibrium state of this medium by compressions and rarefactions. As they are elastic, a force proportional to the particle displacement acts on the particles to return them to their original position. In the so-called SOFAR channel (sound fixing and ranging), a low velocity zone found in the deep oceans typically at depths of 600 to 1200 m, hydroacoustic waves are guided, allowing an efficient propagation through the oceans over long distances without significant damping (Dahlman et al. 2011; Pilger et al. 2017). Infrasound is sound below the human hearing threshold (< 20 Hz). Infrasonic waves are intense low-frequency compressional waves (Dahlman et al. 2011), which propagate as pressure perturbations through the atmosphere with their primary (most efficient) propagation in the stratospheric waveguide, extending up to 40 to 60 km altitude (Le Pichon et al. 2010; Pilger et al. 2017). The propagation media for both infrasonic and hydroacoustic waves are significantly more variable compared to seismic waves; for instance, infrasonic wave propagation is influenced by both small- and large-scale variations in atmospheric conditions such as temperature and wind, making the description of propagation more complicated.

To date, a large number of studies have focused only on a single one of the three waveform technologies and their respective sources and a comprehensive cross-technology study is not yet available. Reviews of natural and anthropogenic seismic sources are given in, e.g. Webb (2008), Bormann et al. (2013), Díaz (2016), and Foulger et al. (2018). Hildebrand (2009) and Dahlman et al. (2011) show and describe various sources of hydroacoustic waves. Different sources of infrasound are described in Blanc (1985), McKisic (1997), Campus and Christie (2010), Dahlman et al. (2011), and Pilger et al. (2017). However, a single source can emit all three wave types (e.g. Green et al. 2013) and waves can be converted from one type to the other (e.g. Heyburn et al. 2020). With regard to the characterization of natural and anthropogenic events and phenomena, the interaction of the waveform technologies is becoming increasingly important, especially with regard to the localization of events and the identification of source mechanisms. In this context, a reliable calibration of sensors will significantly contribute to the improvement of data quality and thus to the localization as well as to the identification of events and their source mechanisms and will allow traceability and inter-station comparability.

Therefore, the aim of this paper is three-fold:

1. Elaboration of necessary requirements for sources to be used for on-site calibration and provision of a brief overview of existing calibration procedures.
2. Provision of a detailed review of natural and anthropogenic sources of seismic, hydroacoustic, and infrasonic waves and their interrelationships, emphasizing source and signal characteristics such as frequency content, typical waveforms, and their variability.
3. Evaluation of these sources with a focus on their use for on-site calibration and assessment of the sources that are most suitable for this purpose.

To address these points, this paper is organized as follows:

First, the calibration of the sensors is considered in more detail, particularly with respect to the necessary properties of a source for use in on-site calibration. Second, a variety of sources for the three different wave types are reviewed in terms of their signal and source characteristics, focusing on the observed waveforms and frequency spectra in the form of power spectral densities and some sources are highlighted with examples recorded at IMS stations. Last, their use for on-site calibration is discussed under the consideration of the requirements elaborated in Sect. 2.

## Calibration

Digital seismometers, hydrophones (underwater microphones), and infrasound sensors (microphones, microbarometers) convert a ground motion or a pressure change into an electrical signal, i.e. they do not express these signals in units of velocity/acceleration or pressure change, but mostly as voltage, current, or number of counts. The sensors of all three waveform technologies are well established, but their respective calibration methods are insufficient, i.e. infrasound and low-frequency acoustic and seismic measurements are currently not fully covered by primary or secondary measurement standards and not traceable to SI, which affects reliability as well as broad acceptance of the measurements. Measurement standards are procedures or objects, which define a relationship to internationally standardized reference objects that are used under carefully controlled laboratory conditions to define the units of physical quantities. In a metrological traceable calibration, a standard is used whose value is traceable: through an unbroken chain of calibrations, a relationship to the definitions of the SI units is established. If the result of the calibration is then expressed with deviation and uncertainty, this calibration result is also traceable. Therefore, traceability describes the property of a result rather than that of a device. In addition, a distinction is made between primary and secondary calibration. In primary calibration, the measurement quantity is traced back to a natural constant or to other physical quantities, up to the SI units. In this case, a comparison is made with an absolute measurement quantity, without prior calibration with other measuring instruments. In comparison, the secondary calibration compares with a reference transducer of the same measurement quantity. Consequently, the calibration of the sensors allows to establish a relation between the input in the respective unit and the output (electrical signal), i.e. it is done to determine the response function of the sensors to a ground motion or pressure change of a certain frequency and amplitude and to define it mathematically (e.g. Willmore 1959; Pavlis and Vernon 1994; Wielandt 2012; Larsonnier et al. 2014).

In order to calibrate seismometers, which measure ground motion, the electrical output should be determined at a ground motion of known amplitude and frequency. There are two established ways to calibrate seismometers: Either the calibration is done mechanically using shake tables under laboratory conditions or purely electrically using calibration coils if they are available in the sensors (e.g. Pavlis and Vernon 1994). The application of shake tables is the most direct way to determine the frequency response of seismometers, but this method is tied to the laboratory (e.g. van Kann and Winterflood 2005; Wielandt 2012). Built-in calibration coils are also applied for in situ calibration (Pavlis and Vernon 1994). Once the relationship between the current in the coil and the equivalent ground motion is known, external electromagnetic excitation from a signal generator can be used to calibrate the seismometer (Wielandt 2012; Larsonnier et al. 2014). A general overview and introduction to seismometer calibration can be found in Wielandt (2012) and detailed descriptions and guidelines are given in Hutt et al. (2009). Larsonnier et al. (2014) compare mechanical with electrical calibration for various seismometers and Klaus and Kobusch (2018) first calibrated seismometers with a multi-component acceleration exciter. As not all seismometers possess calibration coils, other excitation mechanisms need to be applied. A common approach is the comparison of the sensor output with that of a (laboratory) calibrated reference sensor. In this case, the sensor under test and the reference sensor are placed in close proximity to each other during the measurement and it is assumed that they measure the same coherent signal. Excitation sources that can be used include continuous recordings of ground noise (Pavlis and Vernon 1994) or the Earth’s tides (Davis and Berger 2007), the latter covering a frequency range below the one considered in this context.

Both microphones/microbarometers and hydrophones can be calibrated under laboratory conditions, using comparable methods. On the one hand, reciprocity calibration is often used (Vincent et al. 2018); on the other hand, methods based on optical interferometry are applied (e.g. Hayman et al. 2013). The reciprocal calibration method uses three identical instruments, one of which serves as the signal source and a second as the signal receiver. The procedure is repeated with the third sensor as an additional receiver. By repeating the procedure with mutually exclusive pairs of instruments, sensitivities can thus be determined. This method does not require a previously calibrated reference sensor, but is time-consuming due to the measurement arrangements (e.g. Beamiss et al. 2002). A method based on optical interferometry is the calibration of sensors by means of pistonphones, which is based on a sealed cavity driven by a piston. If the volume of the cavity and the volume change caused by the piston are known, the sound pressure can be calculated using the adiabatic gas law. Using, e.g. laser interferometry or a water manometer, the piston displacement and thus the pressure change can be determined and used for the calibration of the instrument in the cavity by measuring and comparing the known pressure change with the sensors output voltage. For calibrating microbarometers using methods based on optical interferometry, pistonphones are commonly used. In the case of hydrophone calibration, laboratory tanks (e.g. Acoustic Pressure Vessel from NPL; Beamiss et al. 2002) or open-water facilities are available. By measuring the output signal of the hydrophone to be calibrated, the sensitivity of the sensor can be calculated by comparing it with the optical determined pressure field (Hayman et al. 2013). In a water tank, hydrophones can also be calibrated by varying their depth within the tank and the resulting change in pressure (e.g. Levin 1973). The in situ calibration of hydrophones is similar to that of seismometers and infrasound sensors by comparison with a reference sensor and the usage of a known source. For example, some IMS stations have been calibrated using airgun shots, explosions, or imploding glass spheres (Harben et al. 1999, 2000). However, ambient sound (Andrew et al 2002) or mid-ocean ridge earthquakes (Hanson and Bowman 2005a) are also used to some extent.

Although microbarometers for infrasound detection can be effectively calibrated in the laboratory, an accurate in situ determination of the frequency response of the entire system deployed in the field is important to ensure comparability as the frequency response is changed on site by, e.g. a wind-noise reduction system (WNRS). This is done by measuring the frequency response of the entire system to a signal (e.g. ambient noise) and comparing it with the known response of a co-located reference sensor (e.g. Gabrielson 2011), using suitable time windows in which the pressure field is constant over the entire system including the reference sensor (Green et al. 2021).

For all three technologies, traceable calibration in the laboratory is the most accurate. However, it is technically difficult to have all field sensors regularly calibrated in the laboratory. On the one hand, laboratory calibration is time-consuming. On the other hand, the sensors are missing in the networks when they are dismantled for calibration, whereby the detection threshold can be influenced and technical requirements for the operation of IMS stations are violated. These technical requirements include “Data Availability” and “Mission capability”; the latter is defined by the CTBTO (2022) as follows: “A mission capable station is one that properly acquires the appropriate amount of data from the sensors and transmits these data to the CTBTO International Data Centre while meeting the data availability, timely data availability, and data quality requirements imposed on a CTBTO International Monitoring System station.”.

The criteria for “Mission Capability” of the respective technologies and stations are defined in detail in the respective operational manuals provided by the CTBTO and can also be found in Pilger et al. (2017). For infrasound arrays, for example, at least 70% of the elements must be operational, while for seismic array stations at least 80% of the elements should be operational. “Data availability” is defined as the percentage of data that is received by the International Data Centre and is specified to be 98% for all three technologies.

Concerning the detection threshold, in arrays, the removal of an element (sensor) changes the array response (array transfer function), which results in degradation of the detection capability as well as of the array performance with respect to the determination of azimuth and velocity (e.g. Chapter 2 in Pilger et al. 2017; Gabler and Ceranna 2021, Gibbons et al. 2015). Furthermore, site-specific factors that can change the frequency response, e.g. the WNRS for infrasound stations, have to be taken into account. Therefore, an on-site calibration is considered, which also includes the site-specific conditions and allows a calibration of the sensors without interrupting the measurement. Reference sensors, so-called transfer standards, are used for this purpose. These are calibrated in a metrological traceable manner and are installed in close proximity to the field sensor to be calibrated (sensor under test). Assuming that the sensor under test and the reference sensor measure the same coherent excitation signal, and that the reference sensor has a precisely determined and traceable response function, the response function of the sensor under test can be determined. Advantages are the undisturbed recording and the additional determination of uncertainties in amplitude and phase.

There are several characteristics that a source should possess in order to be considered as an eligible excitation source for the on-site calibration procedure: (1) It is important that the sources generate signals well above the noise level of the sensors, which also is station and sensor dependent (e.g. Berger et al. 1979); (2) the source should be a so-called ground truth event, i.e. time and location of the source should be known/determinable, and (3), if possible, the magnitude or, in the case of an explosion, the yield may be known/determinable as well; (4) the source should at least excite waves in the frequency range under consideration, i.e. between 0.01 and 20 Hz, and (5) to ensure comparability between calibrations and to detect any drift of the sensor, the source should be reproducible in its characteristics, i.e. magnitude/yield, as well as waveform and frequency content should be stable and comparable between similar events. In addition, it is important that the signals between the sensor under test and the reference sensor are coherent.

## Seismic Sources

Seismic waves are emitted by numerous natural and anthropogenic sources. The most prominent sources being tectonic earthquakes and volcanoes, which are capable of generating seismic waves that are recorded worldwide. However, rivers as well as the oceans and the atmosphere similarly emit recordable seismic waves with a much smaller amplitude by interacting with the solid earth through different processes. In addition to these and countless other natural sources, there are also a number of man-made processes that radiate seismic energy over a broad range of frequencies. Besides explosions, which are observable at greater distances, traffic as well as industrial activities are among the generators of seismic waves.

In a review paper, Díaz (2016) describes various sources of seismic background noise using a single, two-week seismic broadband recording. Additionally, a historical overview of observations of various natural and anthropogenic phenomena is provided, including microseisms and Earth tides. Foulger et al. (2018) give a review of global human-induced earthquakes with reference to HiQuake, the Human-induced Earthquake Database (Wilson et al. 2017). Various anthropogenic processes and some related examples are given that are thought to have resulted in or triggered induced seismicity, which results from the stress perturbations in the crust caused by these anthropogenic processes such as hydraulic fracturing or the impoundment of water reservoirs.

### Natural Sources

#### Tectonic Earthquakes and Tectonic Tremor

About 95% of all tectonic earthquakes observed worldwide occur at plate boundaries, of which the majority (ca. 85%) occurs in subduction zones. The remaining 5% are mainly intraplate earthquakes (Bormann et al. 2013). Tectonic earthquakes occur when the brittle part of the Earth’s crust is subject to stress that exceeds its fracture strength, in fact such stress/strain results from the relative motion of the lithospheric plates with respect to each other, leading to sudden ruptures along existing faults (Bormann et al. 2013). These ruptures, mostly shear ruptures, are characterized by different focal mechanisms, with strike-slip, normal, and thrust (reverse) faulting being the basic mechanisms, but the rupture is generally best described by a combination of these different mechanisms. Although it is generally assumed that deformation, and hence stress, occurs mostly at plate boundaries without deforming the plate interior, earthquakes also occur within the plate interior, especially along ancient rifts and pre-existing faults. The causes of intraplate deformation and earthquakes are still not entirely clear, but possible causes include glacial isostatic adjustments (e.g. Mörner 2009; Brandes et al. 2015), salt tectonics (e.g. Leith and Simpson 1986; Dahm et al. 2011; Katz and Hamiel 2019), or rifting (e.g. Gangopadhyay and Talwani 2003), as well as large-scale tectonic stresses and strength variations within the lithosphere (Talwani 2017; Ghosh et al. 2019).

Earthquake signals are characterized by many parameters, including waveform, signal length, onset time, amplitude, and polarity of the different observed wave phases (e.g. Kanamori and Brodsky 2004; Bormann 2012; Bormann et al. 2013). All these parameters depend on the wave type (body/surface wave) and phase as well as on the source mechanism and duration. In addition, the distance between source and sensor and thus the propagation path of the seismic waves through the Earth’s interior affect the parameters (Bormann et al. 2012, 2013).

The seismic energy emitted by earthquakes is distributed over a wide frequency range (Ide and Beroza 2001). There is a relationship between the frequency band generated and the seismic moment, i.e. there is a dependence of the source spectrum on the event magnitude (e.g. Huerta-Lopez et al. 2000; Bormann et al. 2013), which is described by the Brune model (Brune 1970). The high-frequency component of the source spectrum is determined by the stress parameter, while the low-frequency component is proportional to the seismic moment M_0_ (Atkinson 1993). Earthquakes of a given moment magnitude M_w_ appear to have similar spectral values and shapes in different tectonic regions (Chen and Atkinson 2002), but the measured waveforms and associated spectrograms often differ from each other, especially with respect to the frequency content and distribution of the main energy. This is caused by source, propagation, as well as location effects. During the propagation of the waves from the source to the seismometer site, numerous changes in the waveform occur along the path. Multiple reflections and refractions result in many signal phases, intrinsic attenuation causes frequency-dependent amplitude attenuation and phase shifts, and scattering creates complicated superpositions of waves with different paths (e.g. Chen and Atkinson 2002). Ground conditions at the measurement site play a role (Huerta-Lopez et al. 2000); for example, reverberation occurs in shallow sediment layers, causing frequency-dependent amplification (e.g. Chen and Atkinson 2002). However, the rock material at the source also affects the frequency content. Rautian et al. (1978) observed higher frequencies for earthquakes occurring in crystalline rocks than for earthquakes occurring in sedimentary rocks. In general, the frequency range of small earthquakes is primarily related to the material through which the wave moved, whereas the observed frequencies for larger earthquakes reflect the characteristics of the source (Butcher et al. 2020). Earthquakes can therefore generate frequencies from 0.01 Hz up to several tens of Hertz and are typically of short duration, usually less than a minute, more likely in the range of several tens of seconds (Nakano et al. 2019). The most common frequency range observed lies between 2 and 8 Hz (Kaneko et al. 2018; Nakano et al. 2019). While body waves are characterized by short wavelets, surface waves exhibit long wave trains due to dispersion, which become longer with increase in distance from the source and can last up to several hours for very strong earthquakes. Depending on the epicentral distance, different phases primarily shape the signal: local and regional earthquakes are mainly characterized by crustal and upper mantle phases, whereas teleseismic earthquakes are dominated by body waves (Kennet 2005; Snoke 2009; Bormann et al. 2012).

Another phenomenon that has only become observable and detectable in recent years due to highly sensitive sensors are slow earthquakes. These are observed in the frequency range between 0.1 Hz and 1.0 Hz (Kaneko et al. 2018). Tectonic tremors are associated with the superposition of many low-frequency earthquakes and are observed in the range between 2 and 8 Hz (Shelly et al. 2007; Kaneko et al. 2018). Tectonic tremor signals usually show an emergent onset rather than a distinct impulsive one, are weak, and their duration can range from several tens of seconds to several minutes. In addition, the waveforms are rarely similar to each other (Nakano et al. 2019). Signals in the range below 0.1 Hz are described as very-low-frequency events (VLFE). Both slow earthquakes, tectonic tremors, and VLFE are associated with slow deformation processes and are likely part of the broadband observation of a common source process originating in shear slip (Ide et al. 2007; Kaneko et al. 2018).

There are countless studies where different earthquakes are investigated in terms of their characteristics: Studies dealing with the major earthquakes of the last decades can be found for example in Rhie et al. (2007) and Tajima et al. (2013). Bilek (2010) gives an overview of the large earthquakes along the South American subduction zone, Papadimitriou et al. (2018) describe details of a strong earthquake, and moderate earthquakes are discussed in Craig (2019) and Wimpenny and Watson (2021). Descriptions of source parameters and signals for small earthquakes are given, for example, in Fan and Wallace (1991) and Levin et al. (2010).

**Fig. 1:**
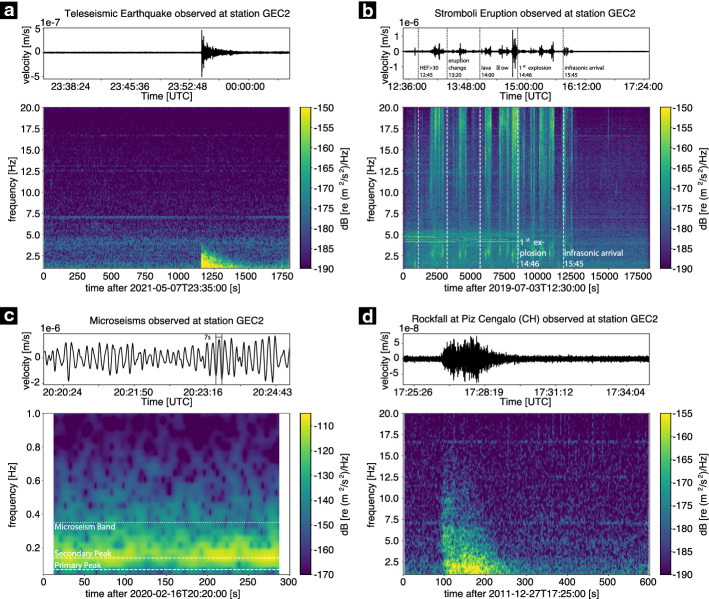
Examples of recorded seismic waveforms and associated spectrograms from natural sources at the seismic station GEC2 (PS19) in the Bavarian Forest, Germany. An example waveform and associated spectrogram for a teleseismic earthquake is shown in **a**. In **b**, the waveform for a volcanic eruption at Stromboli volcano (3 July 2019) is shown. The marked times in the seismogram are based on the eruptive activity description in Andronico et al. (2021). Example waveforms and spectrograms for microseisms and rockfall are given in **c** and **d**. In **c**, the microseism band is marked by a white dotted line; the primary and secondary peaks are highlighted by white dashed lines. The shown rockfall event in **d** occurred at Piz Cengalo, Switzerland, in a distance of approximately 418 km from the station GEC2. The waveforms of **a**, **b**, and **d** are bandpass filtered with a Butterworth bandpass filter between 1 and 20 Hz. For the microseisms a Butterworth bandpass filter between 0.04 and 1 Hz is applied. The spectrograms are calculated with either 90% (**a**, **b**, **d**) or 99% (**c**) overlapping hanning windows with a length of 2^10^ (**a**), 2^12^ (**b**), 2^11^ (**c**), or 2^9^ (**d**) samples, respectively. For more information, see Table 2 (Appendix)

Figure 1a shows an example of a teleseismic earthquake recorded at station GEC2, PS19, Germany. The earthquake had a body wave magnitude of mb 5.7 and the epicentre was located at 18.7° S, 177.4° W in the Fiji Island region in a depth of 384 km, according to the United States Geological Survey (USGS). The waveform (top) is bandpass filtered between 1 and 20 Hz using a Butterworth bandpass filter. In the lower part, the corresponding spectrogram is shown. The spectrogram, which shows power spectral densities for each successive segment, is calculated using Welch’s average periodogram method, a hanning window with a defined length and overlap adapted to the respective data example to ensure comparability between the examples and smoothness of the image (see Table 2, Appendix, for more information on the applied parameters for each example.).

#### Volcanic Tremor, Volcanic Earthquakes, and Volcanic Eruptions

Seismic waves are emitted by various volcanic processes including eruptions, magma flow, shallow gas explosions, shear fractures, and caldera collapses (Schick 1981). Based on their waveform, spectral content, and the medium in which they occur, volcanic seismic signals are further subdivided (Ntepe and Dorel 1990, and references therein; Malfante et al. 2018). Numerous studies show the broadband nature of these signals, which are triggered by a variety of known and hypothetical source mechanisms; however, there is still imperfect knowledge about the source mechanisms of numerous volcano-seismic events (Schick 1981; Chouet et al. 1999; Wassermann 2012).

Volcano-tectonic earthquakes are brittle-fracture events. They exhibit spectral characteristics similar to tectonic earthquakes and are therefore indistinguishable from the latter (Ntepe and Dorel 1990; Chouet 1996; Malfante et al. 2018). The frequency content, determined at the sensor without back-propagation to the source, ranges from 1 to 5 Hz, but can exceed this range for events at greater depths (Wassermann 2012). It should be noted that higher frequencies in particular may not be measurable at greater distances due to absorption effects, and that other factors along the propagation path also influence the signals. Volcano-tectonic earthquakes are characterized by well-defined onsets of P and S waves (Ntepe and Dorel 1990; Wassermann 2012), suggesting source processes in the bedrock, mainly slip along a fault plane, which results from shear failure caused by stress changes induced by magma motion (Chouet 1996; Wassermann 2012; Malfante et al. 2018).

Volcanic tremor is the most commonly measured seismic signal associated with volcanoes and often a sign of high volcanic activity (e.g. Wassermann 2012; Bormann et al. 2013). Tremor is characterized as a monochromatic, continuous seismic signal with prolonged amplitude that can last from a few seconds up to several minutes and days, and sometimes for months or longer, and has a non-impulsive onset (Hofstetter and Malone 1986; McNutt 1992; Chouet 1996; Malfante et al. 2018). Tremor occurs in a narrow-band frequency range of 1 Hz to 9 Hz (McNutt 1992; Wassermann 2012; Malfante et al. 2018) and the source of tremor cannot be precisely localized in time and space (Bormann et al. 2013). Volcanic tremor is the seismic expression for a variety of physical processes and there are many models describing the sources of tremor (Hofstetter and Malone 1986; Haney et al. 2020). Generally, tremor is associated with fluid processes, i.e. it is thought to be caused by the movement of magma in magma chambers or channels beneath the volcano (Hofstetter and Malone 1986; Chouet 1996).

Long-period events show similar tempo-spectral characteristics as those of tremor (emergent signal onset, no distinct body wave arrivals), and are therefore associated with the same source mechanisms (Ntepe and Dorel 1990; Wassermann 2012). They resemble small tectonic earthquakes in duration, but exhibit a different frequency content (1–3 Hz). In addition, so-called very long-period signals in the frequency range between 0.01 and 0.5 Hz have been observed. These are often directly associated with volcanic explosions and can only be observed in the near field (Chouet et al. 1999; Wassermann 2012).

Seismic signals from explosive eruptions show distinct signal characteristics and a frequency content between 0.01 and 3 Hz. In addition, an airwave with the typical sound velocity of 330 m/s usually occurs, which is triggered by the sonic boost during the eruption. Nearly all explosive eruptions are accompanied by tremor (McNutt 1992; Malfante et al. 2018) and are associated with sudden magma extrusion as well as ash and gas emission (Wassermann 2012; Malfante et al. 2018).

So-called hybrid events, events with a combination of different characteristics, have been observed at Redoubt Volcano, Soufrière Hills Volcano, Montserrat, and Mount St. Helens Volcano (Wassermann 2012; Malfante et al. 2018, and references therein). Furthermore, seismic signals are also generated by other processes often accompanying volcanic activity such as rockfall, landslides, pyroclastic density flows, and lahars; these are characterized by high frequencies (> 5 Hz) and spindle/cigar-shaped envelopes and show very complex waveforms (Wassermann 2012).

An example for the recorded waveform of a volcanic eruption at Stromboli volcano is given in Fig. 1b, showing the bandpass filtered waveform (top) and the respective spectrogram showing the power spectral density (bottom). The marked events in the seismogram are based on the description of the eruptive activity found in Andronico et al. (2021). All events were shifted in time by about 3 min, which corresponds to the travel time between Stromboli and the seismic station GEC2 (PS19). Around 12:45 UTC (12:48 UTC at GEC2), the hourly explosive frequency (HEF) exceeds 30, which is a very high value (Andronico et al. 2021). Around 13:20 UTC (13:23 UTC at GEC2), a change in eruption style from jet-like to violent spattering is observed. A small lava flow occurs around 14:00 UTC (14:03 UTC at GEC2), which is a very unusual event. At about 14:43 UTC (14:46 UTC, GEC2), lava began to be ejected simultaneously from almost all vents, and approximately 2.5 min later the first explosion occurred, the signal of which was recorded at GEC2 at about 14:48 UTC. One hour later, the infrasonic signal of the first explosion also reaches the seismic station.

#### Microseisms

Globally, both close to the coastline and in the deep interior of the continents, high levels of seismic background noise are observed in the frequency range between 0.05 and 2 Hz, with dominant peaks near periods of 7 s (0.14 Hz) and 14 s (0.07 Hz), respectively (e.g. Longuet-Higgins 1950; Bromirski et al. 2005; Aster et al. 2010; Ardhuin et al. 2015). These continuous oscillations are termed microseisms. Figure 1c gives an example record of microseisms. The primary and secondary peaks are clearly visible.

The energy of these oscillations is mostly generated by ocean waves and seismic signals are generated by distinct mechanisms that couple that energy into ground motion (Aster et al. 2010; Ardhuin et al. 2011; Bromirski et al. 2013). According to the observed peaks in the frequency spectrum and based on the suspected mechanisms of their origin, microseisms are further subdivided into the primary (0.04–0.17 Hz) and secondary microseism (0.08–0.34 Hz; Ardhuin et al. 2011). Primary microseisms are generated by direct interaction of ocean pressure fluctuations with the seafloor and the peak is observed at the frequency of ocean waves (Traer et al. 2012; Bromirski et al. 2013; Traer and Gerstoft 2014). Secondary microseisms, which show a peak at double the frequency of ocean waves, are mostly generated by the interaction of ocean wave trains propagating in opposite directions generating standing waves (Longuet-Higgins 1950; Ardhuin et al. 2011; Traer et al. 2012; Traer and Gerstoft 2014). These waves are caused either by wave reflections from the coastlines or by opposing winds and storm systems (Aster et al. 2010). Bromirski et al. (2013) show that the sources of secondary microseisms are found in near-coastal areas, which were theoretically explained by Longuet-Higgins (1950). According to Bromirski et al. (2005), the source of significant primary microseisms is found in shallow water.

#### Mass Movements: Rockfalls, Landslides, Avalanches

Various mass movements including avalanches, landslides, and rockfalls generate seismic signals. Although these are different in nature and differ in their characteristics, they can all be considered as moving seismogenic sources. Common to all of the above processes is the downslope movement of material due to gravitational processes. In general, the seismic signatures of mass movements are very complex due to the existence of many moving sources and the influence of the medium, topography, and small-scale local conditions (Suriñach et al. 2005).

The characteristics of seismic signals caused by avalanches are described in numerous studies. One of the first studies was published by Lawrence and Williams (1976), who showed that avalanches have characteristic signals distinguishing them from other events. The seismic signals are of long duration (> 10 s), lack impulsive onsets and show a specific spindle pattern that is also observed for pyroclastic flows from volcanoes (Kishimura and Izumi 1997; Lacroix et al. 2012; Perez-Guillen et al. 2019). However, the signal shape depends both on the path of the avalanche (Suriñach et al. 2001) and on the relative position of the seismometer to the avalanche (Biescas et al. 2003). The majority of seismic energy lies below 30 Hz with a central frequency below 10 Hz, but higher frequencies are occasionally observed (Kishimura and Izumi 1997; Lacroix et al. 2012; Perez-Guillen et al. 2019). Because of the moving nature of the avalanche, the frequency content of the signals is not stationary. The dominant peak shifts to higher frequencies as the avalanche approaches the sensor resulting in a triangular shape of the spectrograms (Kishimura and Izumi 1997; Biescas et al. 2003; Perez-Guillen et al. 2019), which seems to be a general and independent feature of these mass movement phenomena, regardless of the location and type of flow according to Suriñach et al. (2005).

The first described seismic observations of a landslide was made by Galitzin (1915) and Jeffreys (1923). Berrocal et al. (1978) show that seismic signals of a landslide could be detected up to a distance of almost 3000 km. Weichert et al. (1994) review a number of well-known cases of seismic events associated with landslides and their possible mechanisms. In general, seismic recordings of landslides display an emergent behaviour (Weichert et al. 1994). During a landslide, different processes occur, which can be divided into rockfall, granular flow, and slopequakes (Provost et al. 2018). Granular flow (wet/dry debris or rock flows) shows seismic signals that are cigar-shaped and can last up to several thousand seconds. No phases can be distinguished in the seismogram and the frequency content is greater than 10 Hz. Slopequakes correspond to sources within the landslide body and usually show signals of short duration (< 1–2 s). The associated spectrogram has a triangular shape, comparable to the observation from avalanches. The seismic signals for rockfall during a landslide clearly show the individual impacts both in the waveform and the spectrogram. The frequency content is mainly above 10 Hz, but frequencies below 10 Hz are also observed for individual impacts. P and S waves are difficult to distinguish and surface waves dominate (Provost et al. 2018). A review of historical landslides caused by earthquakes is given in Keefer (2002). Brodsky et al. (2003) present seismically determined bounds on the frictional coefficients for three large volcanic landslides. A good overview of published seismic data on landslides and the signal properties for numerous examples is given in Provost et al. (2018).

The seismic properties of rockfall are investigated by Deparis et al. (2008) and Feng et al. (2019). The seismic signals of different events show many complex waveforms with emergent signal onsets and include both body and surface waves (Deparis et al. 2008). Individual impacts of the rock on the slope are seen as individual peaks in the seismic signal, but the waveform as well as the spectrogram generally depend on the local topography, material, geometry of the slope, and distance between the source and the sensor (Feng et al. 2019). The main part of energy is observed for frequencies of 10–60 Hz and 80–90 Hz. Figure 1d shows an example of a rockfall at Piz Cengalo, Switzerland, recorded at a distance of approximately 418 km from the source at GEC2, PS19 in Germany, with the main energy found below 10 Hz.

### Other Natural Sources of Seismic Waves

#### Earth Hum, Solid Earth Tides

In the seismic record, continuous oscillations with frequencies of 0.3 to 20 mHz are termed Earth’s hum. It has been first reported for large earthquakes, but has also been observed worldwide at sites on seismically quiet days with a constant level and only little seasonal variations (Nawa et al. 1998; Rhie and Romanowicz 2004; Tanimoto 2005; Webb 2007). The observed frequencies fall within the range of the theoretical eigenfrequencies of the fundamental spheroidal modes of the Earth. Nawa et al. (1998) have first identified these oscillations and suggested them to be not of earthquake but of atmospheric or oceanic origin. A mechanism based on the turbulent atmosphere has been described by Tanimoto (1999), but atmospheric sources seem to be negligible (Webb 2008) and the Earth’s hum is rather excited by the interaction of oceanic infragravity waves with the Earth. The study of Rhie and Romanowicz (2004) provides an observational evidence for this oceanic excitation hypothesis, for which the theoretical evidence is given in Tanimoto (2005). As the sources of the Earth’s hum are located within the oceans, investigations on the source regions reveal that the Earth’s hum originates in the northern and southern oceans during the Northern and Southern Hemisphere winters, respectively (e.g. Rhie and Romanowicz 2004, 2006; Bromirski and Gerstoft 2009; Ermert et al. 2017). Webb (2007, 2008) demonstrates that the hum originates from the interaction of infragravity waves with the continental shelves. While Webb (2008) also proposes the interaction of infragravity waves over the deep ocean basins as possible source, no indication for that is found by Bromirski and Gerstoft (2009) or Ermert et al. (2017). A first observation of the hum on ocean bottom seismometers is presented by Deen et al. (2017).

Besides the Earth’s hum, the solid Earth tides may be observed in the seismic record, showing characteristic periods of 12 and 24 h, respectively, originating from the gravitational effects of the Sun and Moon leading to elastic deformations of the solid Earth (e.g. Díaz 2016).

#### Atmospheric Phenomena: Thunderstorms and Meteoroids

Kappus and Vernon (1991) and Lin and Langston (2009) investigated waveforms of ground motions generated by thunder and found signals characterized by impulsive onsets showing a characteristic N-shape and short signal durations of 5 s to more than 30 s. The signals have very broad spectra with peak frequencies between 6 and 12 Hz and reverberations with a frequency between 4 and 7 Hz and. Depending on the intensity of the lightning and thunder (e.g. type of lighting, direction of current, energy), the amplitudes show great variations. Lin and Langston (2009) propose that ground motions are induced by acoustic to seismic coupling, similar to seismic signals generated by the shock wave of meteoroids.

Ishihara et al. (2003), Langston (2004), and Pujol et al. (2005), among others, analysed the shockwaves from meteoroids recorded by seismographic networks to reconstruct the trajectory of meteoroids. Edwards et al. (2008) give a review of the history of seismic signals generated by meteoroids including waveform characteristics and possible source mechanisms. Seismic signals produced by meteoroids recorded on seismometers show distinct waveform characteristics. Several effects cause the generation of seismic signals. First, the shock wave of a meteoroid can be measured directly, which occurs when the meteoroid moves through the atmosphere at supersonic speeds (e.g. Ishihara et al. 2004). A shock wave usually exhibits an impulsive first negative ground motion in the seismogram, a so-called reversed N shape, followed by oscillations longer than 10 s, and is characterized by high frequencies (1–10 Hz). Typical distinct P- and S-wave arrivals are usually not present. In addition, an apparent velocity of about 330 m/s is evident (e.g. Ishihara et al. 2003; Langston 2004; Kumar et al. 2017). Second, if the shock wave is strong enough, its energy couples with the ground and generates ground-coupled acoustic waves that can be measured at the sensors (e.g. Ishihara et al. 2004; Arrowsmith et al. 2007a). In the majority of observed cases, this occurs directly, i.e. by local loading of the surface by the overpressure of the incident shock wave. An overview of the mechanisms can be found in Edwards et al. (2008). Arrowsmith et al. (2007a) observed four classes of seismoacoustic waves related to meteoroids depending on their waveform characteristics. These range from impulsive, showing only a sharp high-amplitude initial onset with a short duration (< 10 s), over reverbatory signals, displaying a high-amplitude initial onset followed by long-lasting reverberations (< 80 s), to dispersed signals with no sharp onset. Not only the shock wave, but also the impact of the meteoroids on the Earth’s surface generates seismic signals, which is very rarely observed (Edwards et al. 2008).

#### Seismic Events Related to the Ice Masses

The ice masses of the Earth produce a variety of seismic signals. Especially glaciers generate signals related to their movement that comprise a wide range of waveforms and frequency contents. These events include rapid retreat, disintegration of large ice volumes, sliding at the base due to glacial flow, ice fracturing, or calving events. The source mechanisms generating the observed seismic signals are not fully understood and further depend, e.g. on the properties of the glacier (Hammer et al. 2015 and references therein).

Calving events show emergent, long seismic signals of 4–10 s duration or longer in a low-frequency band between 1 and 3 Hz (e.g. Richardson et al. 2010; Walter et al. 2012). Typical seismic signals with clear P- and S-wave onsets are rarely observed; instead, the waveforms display a complex behaviour with sequences of several signals (Köhler et al. 2012). Some signals are dominated by Rayleigh and Love waves with frequencies below 0.1 Hz. The mechanisms behind the seismicity related to iceberg calving are discussed in Bartholomaus et al. (2012). They find that the detachment of an iceberg form the terminus of a glacier as well as the iceberg-sea surface impact are the main mechanisms producing seismic energy in the frequency range between 1 and 20 Hz.

Motion at the glacial base is characterized by long, emergent, low-frequency, monochromatic signals in the range of 1 to 2 Hz (Hammer et al. 2015). Ekström et al. (2003) detected moderate earthquakes and classified these as the new class of as glacial earthquakes. They found the seismic records of these events observed at larger regional distances to be depleted of higher frequencies. Compared to tectonic earthquakes of similar size, the events were of much longer duration (30 to 60 s). The events displayed unusually small amplitudes on the short-period seismograms and complex, low-frequency surface waves with large amplitudes on the long-period seismograms, indicating a source process of long duration related to the strike-slip motion of downhill sliding of the glacial ice mass. Since most events occur in late summer, glacial earthquakes are associated with large ice-loss events such as calving of large icebergs. The waveforms show long periods (> 30 s) with large amplitudes and are registered worldwide (Nettles and Ekström 2010). They have significant energy at periods of between 20 and 100 s, and a much longer duration than tectonic earthquakes of similar magnitude (Tsai et al. 2008).

Microseismicity is also observed at glaciers (West et al. 2010): There are low-frequency events with emergent onsets and long codas (complex wavetrain following the primary arrival produced by scattering of the wavefield; Bormann 2012) dominating the range between 6 and 15 Hz, high-frequency impulsive arrivals with a dominant frequency between 20 and 35 Hz, and hybrid events with both impulsive onsets and low-frequency codas.

#### Natural Seismic Noise

Naturally observed noise sources include rivers and wind. They produce permanent non-coherent signals over a wide frequency range (1 mHz–50 Hz) are neither well localized in space nor in time and are most apparent through their seasonal variations (Burtin et al. 2008). Smith and Tape (2019) observed the influence of a river around 10 Hz and Díaz (2016) reported that variations in the discharge of a nearby river are evident in both the seismogram and the spectrum. Burtin et al. (2008) observed temporal and spatial variations in the entire frequency spectrum between 10^–3^ and 100 Hz along the Trisuli River, Nepal, which are related to monsoon rainfall, snow melting, and fluctuations of precipitation. The seismic noise is partly generated by stream turbulence, but ground vibrations generated by bed load transport also play a role (Burtin et al. 2008).

Variations in noise levels are observed across all frequencies as a function of wind speed. Wind noise is spatially and temporally variable on small scales and produces a variety of seismic waveforms similar in amplitude to microseismicity (Johnson et al. 2019; Smith and Tape 2019). In addition to the direct interaction of wind with the sensor, near-surface wind turbulence and long-wavelength atmospheric pressure waves can also generate seismic signals. Johnson et al. (2019) observed earthquake- and tremor-like signals in the range between 1 and 8 Hz due to the interaction of the wind with obstacles at the surface.

### Anthropogenic Sources

#### Nuclear and Chemical Explosions, Quarry Blasts, Mining Activities

An explosion is a process in which a large amount of energy is released in a relatively short time. We consider here explosions that are man-made and mostly controlled, i.e. the location and time of the source are often known and the yield can be estimated from the used substances and volumes. While smaller explosions are observed locally and regionally on the seismic record, large nuclear explosions can be recorded worldwide. In addition to nuclear explosions, there is a variety of different chemical explosion types. These include single explosions, multiple-hole instantaneous explosions, and ripple-fired explosions, all of which are associated with mining operations. Other chemical, often accidental explosions are also observed at seismic stations (e.g. Koper et al. 2002; Pilger et al. 2021b).

The different explosion types can be distinguished from each other on the basis of several discriminatory features such as radiation pattern and signal duration, which also allow a discrimination between explosions and earthquakes (e.g. Kim et al. 1994; Stump et al. 2002; Richards and Kim 2007). The most significant difference between explosions in general and earthquakes is observed in the radiation pattern. Compared to earthquakes, explosions are characterized by an impulsive initial outward compression motion in all directions, resulting in an N-shape pulse signature (e.g. Richards and Kim 2007). In addition, explosion signals show a shorter rise time and duration as well as a higher frequency content compared to earthquakes. Unlike tectonic earthquakes, both nuclear and chemical explosions are dominated by P waves and radiate weaker S waves over all frequencies, especially for frequencies greater than 10 Hz (Wüster 1993; Kim et al. 1994; Prastowo and Madlazim 2018; Wang et al. 2020). The P wave is then followed by high amplitude Rayleigh waves, which are strongly affected by the explosions’ depth and the overlying topography (Stevens et al. 2017). Because of the strong P waves the P/S amplitude ratio, which is larger for explosions than for earthquakes, is one way to distinguish between them, in particular for larger magnitudes (M > 4; Koper et al. 2008; Wang et al. 2020). The mechanisms underlying the generation of seismic waves by an explosion are complicated and are in detail described in Johnson (1994). A purely explosive source cannot explain all the waveforms and radiation patterns observed for (underground nuclear) explosions (Toksöz and Kehrer 1972). Therefore, different source models for nuclear explosions exist which are reviewed in Massé (1981).

**Fig. 2:**
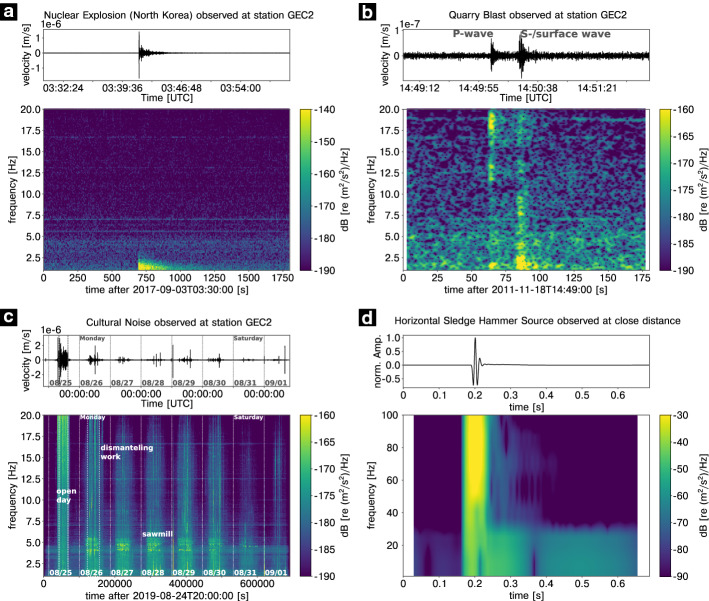
Examples of recorded seismic waveforms and associated spectrograms from different anthropogenic sources at the seismic station GEC2 (PS19) in the Bavarian Forest, Germany (**a**, **b**, **c**,) and at a 4.5 Hz geophone (**d**). The waveform and spectrogram of a nuclear explosion (DPRK 6) in North Korea are shown in **a**. **b** shows the waveform and spectrogram of a quarry blast recorded in a short distance from the source. The first signal, representing the P wave, is followed by a second signal about 20 s later, which represents the S wave/surface waves. The record of cultural noise for eight consecutive days is given in **c**. On the first shown day (Sunday), there has been the open day at the seismic station. The beginning and the end of the open day are marked by vertical white dashed lines. Note the higher energy at higher frequencies on Monday, which is related to dismantling work from the previous day. Higher energy between 3 and 6 Hz on weekdays is associated with human activities during the working hours, i.e. forestry work or truck traffic. According to Marty et al. (2021), peaks at 4 and 6 Hz are related to a sawmill. The waveform and associated spectrogram of a horizontal hammer blow against a steel plate coupled to the ground by steel spikes at its bottom recorded by a close-by horizontal geophone is shown in **d**. The waveforms of **a**, **b**, and **c** are bandpass filtered applying a Butterworth bandpass filter between 1 and 20 Hz. For the hammer blow example, a Butterworth bandpass filter between 1 and 100 Hz is applied. The spectrograms are calculated with either 90% (**a**, **b**, **c**) or 95% (**d**) overlapping hanning windows with a length of 2^10^ (**a**), 2^9^ (**b**), 2^15^ (**c**), or 2^9^ (**d**) samples, respectively. For more information, see Table 2 (Appendix)

As an example waveform of a nuclear explosion the recorded seismogram of the last (i.e. sixth) nuclear test by the Democratic People’s Republic of Korea (DPRK), conducted on September 3rd, 2017 at 03:30:01 UTC, is shown in Fig. 2a. The test has been the most powerful test ever conducted by DPRK and it took place at Mt. Mantap (41.3° N, 129.1° E) in a depth range of approximately 2 km below the surface (e.g. Wang et al. 2018). The explosive yield was in the range of 160 to 400 kilotons of TNT equivalent (e.g. Gaebler et al. 2019). The explosion produced signals with a magnitude of mb 6.1 on the body wave magnitude scale (e.g. Koch and Pilger 2019). About 8 to 9 min after the actual explosion, another seismic signal with a body wave magnitude of mb 4.6 was recorded, caused by the collapse of the cavity (e.g. Myers et al. 2018, 2019). Gaebler et al. (2019) find that the seismic waveforms, especially long-period ones, of all North Korean nuclear tests resemble each other, concluding that all events have a similar radiation pattern in common.

The observations and spectra of different chemical explosion types are described in Kim et al. (1994). They find that ripple-fired blasts show distinct frequency bands related to the repetitive nature of the source, which allows a discrimination from instantaneous blasts or earthquakes. Stump et al. (2002) show that mining blasts display a repeated pattern in the frequency domain due to interference effects resulting from a regular pattern of delays between individual explosions during the blast. Studies investigating the difference between quarry blasts and earthquakes are numerous (e.g. Hedlin et al. 1989; Allmann et al. 2008). They observe a characteristic time-invariant spectral behaviour (spectral banding) for ripple fired quarry blasts which can be used as discriminant between single-fired and ripple-fired explosions. Seismic signals of quarry blasts have a higher P- to S-wave ratio compared to earthquakes (Horasan et al. 2009). A recent study uses local to regional distance surface waves to estimate magnitudes and locations of industrial blasts (Kintner et al. 2021). Figure 2b gives an example of the recorded waveform of a single quarry blast with a source-receiver distance of approximately 167 km. The first signal, the P wave, is followed about 20 s later by a second signal of slightly higher amplitude, which corresponds to the S- and surface waves.

#### Cultural Noise

Seismic signals caused by human activity such as traffic or industrial machinery are referred to as cultural seismic noise. It is characterized by daily and weekly variations in its intensity (Bonnefoy-Claudet et al. 2006; Groos and Ritter 2009; Sheen et al. 2009) and is usually attenuated relatively quickly, i.e. it is observed only at a short distance from its source (Riahi and Gerstoft 2015). Lecocq et al. (2020) recently showed that the lockdown due to the COVID-19 pandemic resulted in the longest reductions in global noise levels to date. Cultural noise is associated with frequencies in the range above 1 Hz (e.g. Bonnefoy-Claudet et al. 2006; Groos and Ritter 2009; Sheen et al. 2009; Smith and Tape 2019; Cannata et al. 2021).

Sheen et al. (2009) observed a correlation between seismograms in the 0.01 to 0.05 Hz frequency range and train schedules. Brenguier et al. (2019) and Pinzon-Rincon et al. (2021) describe the use of freight trains as a seismic source to study near-surface structures. The waveforms show similarity to episodic tectonic tremors with clear spectral lines in the 1–20 Hz range. Wind turbines are another source of anthropogenic noise (Stammler and Ceranna 2016; Marcillo and Carmichael 2018). Lacroix et al. (2012) observed that cars generate long spindle-shaped signals (50 s) in the frequency range above 5 Hz.

In the frequency range between 0.6 and 1 Hz, natural and anthropogenic sources overlap, making differentiation more difficult. In this range, mainly wind-induced building oscillations occur (Groos and Ritter 2009). An example of cultural noise is given in Fig. 2c, which was recorded for eight consecutive days including the open day at the seismic station GEC2, PS19, highlighting the broadband nature of cultural noise. Here, the noise is especially related to people walking in close proximity to the sensor. Note the higher energy at higher frequencies up to 20 Hz on Monday, which is related to dismantling work from the previous open day. The higher energy between 3 and 6 Hz during the working hours on the weekdays is associated with human activities, i.e. forestry work or truck traffic. The peaks at 4 and 6 Hz are related to a nearby sawmill (Marty et al. 2021).

#### Controlled Sources: Drop Weights, Sledge Hammers and Vibration Sources

Seismic sources commonly used for near surface investigations and shallow reflection purposes by industry, e.g. sledge hammer, vibration sources, shot guns, and drop weights, are reviewed in Miller et al. (1986, 1992, 1994), Pullan and MacAuly (1987), Abe et al. (1990), Doll et al. (1998), and Herbst et al. (1998), among others. They found a site dependence of the produced signals concerning the excited waveforms, frequency content, amplitudes, as well as repeatability. As high-frequency signals are needed for typical near-surface seismic reflection surveying, the mentioned studies primarily focused on the excitation of frequencies higher than 50 Hz. Note that most studies used 28 Hz or 100 Hz, occasionally 10 Hz or 4.5 Hz geophones, which are rarely corrected for instrument responses. The frequency spectra shown in Herbst et al. (1998) show peaks in the frequency range between 40 and 120 Hz, but low frequencies are also excited in their tests.

The basic principle for both sledge hammer and (accelerated) drop weight sources is a mass of weight hitting a plate on the ground from a certain height and transferring momentum into the ground. The force on the ground creates a seismic wave that propagates through the subsurface (e.g. Neitzel 1958). The properties of sledge hammer sources were investigated in, e.g. Keiswetter and Steeples (1995). The hammer mass and velocity as well as the mass and size of the coupling plate were varied. Brom and Stan-Kłeczek (2015) found a dominant frequency of 50 to 60 Hz and Hartantyo (2016) a frequency range of 30 to 80 Hz for hammer blows, while Malovichko et al. (2005) observed frequencies of 5 to 30 Hz. Keiswetter and Steeples (1995) show an increase in low frequencies with increase in hammer mass. The variations in observed dominant frequencies are related to the seismic energy, which increase with increase in hammer and/or plate mass, resulting in a larger proportion of lower frequencies. Figure 2d shows an example of a waveform recorded close to a horizontal sledge hammer blow against a steel plate, which is coupled to the ground by steel spikes at its bottom. Although the highest energy is found at frequencies larger than 40 Hz, a considerable amount of energy at low frequencies is present. Compared to the previously published data, a very broadband signal down to a frequency of 1 Hz is shown here. This may be due to the source used (excitation of SH-waves by a horizontal hammer blow compared to P-wave sources), but also due to the use of 4.5 Hz geophones, which have a flat response function in the low-frequency range compared to the 28 Hz or 100 Hz geophones mostly used in the studies mentioned. For near surface applications transient signals are desirable. As in the example shown, they are characterized by a short signal duration, which results in a broadband spectrum. These signal types allow a higher resolution in seismic imaging of the subsurface.

The characteristics of drop weights have been studied by Domenico (1958), Neitzel (1958), Ganguly and Moissa (2005), and others. They found that the dominant frequencies are lower than those of dynamite shots/explosions, but the source characteristics have very high similarity with the dynamite signature. Hartantyo (2016) for example measured frequencies between 30 and 90 Hz for their mobile weight-drop source. Abe et al. (1990) observed that for weight-dropping sources the dominant excited frequency range depends on the ratio of the weight and coupler masses. The weight mass needs to be much smaller than the coupler mass to excite low frequencies, which are dominant over higher frequencies. A specific example of the use of drop weights can be found in Jolly et al. (2012). They used a helicopter-based high-impact mass drop with weights of 700 kg (sand) from a height of 310–380 m to investigate seismic properties of a volcano. Frequencies between 2 Hz and more than 10 Hz were generated.

Vibrating sources are the most commonly used non-explosive sources in seismic exploration (e.g. Keary et al. 2002). Vibrators are used to transmit seismic energy into the subsurface. The signal typically employed is a so-called sweep. The sweep signal is precisely known and therefore repeatable. A sweep is defined as a continuously oscillating signal of constant amplitude with monotonically varying frequency, i.e. each frequency within the defined bandwidth occurs only once (Goupillaud 1976; Rietsch 1977). There are different types of sweep signals. In a linear sweep, the frequency varies linearly with time, i.e. it increases (up-sweep) or decreases (down-sweep) monotonically. A characteristic of a linear sweep is the flat frequency response over the entire bandwidth of the source (Goupillaud 1976; Rietsch 1977). In addition, there are various nonlinear sweep signals, e.g. exponential, inverse-linear, or quadratic sweep. An overview is given in Goupillaud (1976). Most vibration sources provide a bandwidth between 10 and 80 Hz (e.g. Keary et al. 2002), but slightly lower frequencies (down to 5 Hz; e.g. Wei and Phillips 2013) can also be generated. The lower-frequency limit is given by mechanical and hydraulic constraints, such as the maximum displacement of the reaction mass (e.g. Bagaini 2008; Wei et al. 2018). Several studies have addressed the question of how to lower the minimum frequency limit. Meier et al. (2016) developed a counter-rotating eccentric-mass vibrator that can produce measurable frequencies starting at 0.5 Hz (Wei et al. 2018). In addition to developing new vibrators, other sweep definitions can also shift the lower-frequency limit to smaller frequencies (e.g. Dean 2014; Reust et al. 2015; Tellier et al. 2015). In addition to large vibrotrucks (usually hydraulic vibrators), there is a growing number of small electro-mechanical vibrators for near surface applications (Keary et al. 2002).

In addition to the above-mentioned sources, pyrotechnics (e.g. Benjumea and Teixidó 2001; Brom and Stan-Kłeczek 2015) or a rotating eccentric mass (ACROSS seismic source; Kasahara et al. 2015), among others, were used as seismic sources. They showed maximum amplitudes for the frequency range of 50 to 90 Hz for the pyrotechnic sources (Brom and Stan-Kłeczek 2015) and 10 to 50 Hz for the ACROSS seismic source (Kasahara et al. 2015). Toney et al. (2019) describe the use of an industrial pile driver as a broadband and repeatable seismic source that generates frequencies in the range of 1 to 3 Hz. Yokota et al. (2004) describe the use of percussion drill as an energy source for seismic surveys while drilling (SWD). The signals from percussion drilling are dependent on the blow rate and force of the drill and are band-limited (monochromatic) under normal use. The frequency content of the generated signals can be varied by manually controlling the hydraulic pressure and thus the blow rate and force of the drill bit. In Yokota et al. (2004), the time series as well as the spectra are shown for normal use, an “on–off” experiment, and linear up and down sweeps. The latter two procedures can excite a wider band of frequencies between 20 and 90 Hz.

#### Induced/Triggered Seismicity

Anthropogenic seismicity termed as induced and/or triggered is occurring as a consequence of various man-made processes. These earthquakes, just like tectonic earthquakes, occur when the stress field of the crust changes. Since the stress state of the crust is close to failure, even small perturbations of this state can lead to failure and earthquakes.

The term “induced” in this context describes seismicity caused by processes that produce a stress change similar to the ambient shear stress. In contrast, the term “triggered” is used when the stress perturbation is only a fraction of the surrounding shear stress field. Most earthquakes are triggered; only a minority are induced earthquakes. Underground mining may cause induced earthquakes, as stress changes comparable to the natural stress field can be generated (McGarr et al. 2002).

Foulger et al. (2018) review numerous global induced earthquakes with reference to HiQuake (Wilson et al. 2017), describing the related anthropogenic processes and providing examples of triggered seismicity. These include earthquakes triggered by surface operations (adding/removing mass, by, e.g. water impoundment behind dams, construction of tall buildings, quarrying), extraction processes from the subsurface such as groundwater extraction or mining, and injection of liquids and gas into the subsurface for different purposes. In addition, relationships and correlations between these processes (e.g. rate/volume of injection) and the characteristics of the induced seismicity (number of earthquakes, magnitude, and seismic moment release) are highlighted. McGarr et al. (2002) provide an overview of case studies and possible causative activities and mechanisms. Kundu et al. (2015) show that anthropogenic processes related to groundwater extraction can lead to stress perturbations by crustal unloading, that might have triggered the 2015 Mw 7.8 Gorkha, Nepal, earthquake. The influence of surface mining by investigating seismic events near open pits is discussed by Kocharyan and Kishkina (2018).

Examples of seismicity in relation with hydraulic fracturing are given in Das and Zoback (2011). They observed seismic events of 10 to 100 s in duration in the frequency range between 10 and 80 Hz without any distinct P- and S-wave arrivals. In Nicol et al. (2011) and Horton (2012), cases of seismicity correlated with fluid injection and extraction are described and implications on the dependency between temporal and spatial occurrence, size, and number of events with injection/extraction rates and volumes are drawn. Dokht et al. (2020) observe and analyse many events with similar characteristics to tectonic earthquakes up to 100 km from a mining site that had been triggered by the mining activities. Examples of different seismic signals related to mining processes are also described in Malovichko (2012).

### Non-Seismic Noise

The term noise is generally used for ambient vibrations of the ground and is divided into seismic and non-seismic noise. Seismic noise is unwanted ground motion, whereas non-seismic noise is caused by various local conditions (Doody et al. 2018). These include seismometer self-noise as well as seismometer sensitivity to local pressure and temperature fluctuations and variations in the magnetic field (e.g. Beauduin et al. 1996; Forbriger 2007; Doody et al. 2018).

At long periods greater than 100 s, the self-noise of the instrument, that is the self-noise of the digitizer in combination with the seismometer, and the sensitivity of the instrument to non-seismic noise sources play a key role (e.g. Doody et al. 2018; Ringler et al. 2020a).

The noise generated by pressure fluctuations is mainly caused by the elastic response of the Earth to these pressure variations. Thereby, the attraction of the air masses (e.g. induced by cold and warm atmospheric fronts) and the resulting deformations of the Earth’s crust result in accelerations, strains, and tilts.

A large part of the observed noise below 0.01 Hz is caused by local temperature fluctuations (Beauduin et al. 1996; Doody et al. 2018). Most of the physical and geometrical characteristics of a device vary with ambient temperature fluctuations. One type of temperature-related noise is due to thermal expansion, i.e. the change in linear dimensions of the instrument’s elements, which is recorded by the seismic sensor as ground motion (Kislov and Gravirov 2012). Additionally, tilt due to differential thermal expansions between the levelling feet occur (Sleeman and Melichar 2012).

Broadband seismometers are sensitive to variations in the Earth’s magnetic field with a period between 40 and 800 s (0.00125–0.025 Hz), e.g. caused by magnetic storms (Forbriger 2007; Ringler et al. 2020b). The sensitivity to magnetic field fluctuations varies from sensor to sensor and depends on the installation. The variations in the magnetic field produce an apparent acceleration of the seismic sensor due to the ferromagnetic properties of the suspension spring or other components (Forbriger et al. 2010).

## Hydroacoustic Sources

As about 70% of the Earth’s surface is covered by water and most tectonic plate boundaries are located below the oceans, many sources emitting hydroacoustic waves are located in the ocean region. Natural sources include marine life, especially whales and dolphins, but also marine volcanic activity and earthquakes. In addition to the cryosphere and its interaction with water, wind and weather also generate detectable hydroacoustic waves. On the anthropogenic side, the largest source is ship traffic, but also industrial activities such as drilling or offshore wind turbines and especially marine seismic exploration contribute significantly to the noise level in the ocean through their emitted hydroacoustic sound. An overview of various anthropogenic sources of hydroacoustic waves is given in Hildebrand (2009). Detailed studies on anthropogenic sources are found in Bohnenstiehl et al. (2012) and Wiggins et al. (2016). Marine volcanism is examined in detail in Tepp and Dziak (2021) and Talandier et al. (2006) provide an overview of signals associated with the cryosphere. For a general overview of noise in the ocean, see Miksis-Olds and Nichols (2016). Many studies focus on the sounds of marine mammals; examples are Nieukirk et al. (2004) or Wiggins et al. (2016).

While seismic waves can be detected with seismometers on land, permanent monitoring of the oceans is technically more difficult. Nevertheless, only a few monitoring stations distributed over the oceans are necessary, because of the efficient propagation of hydroacoustic waves in the SOFAR channel over long distances. The signals are divided into tertiary (T) phases and hydroacoustic (H) phases according to the source location. H phases are generated by sources located in the water (e.g. ship traffic), while T phases are signals converted from seismic to acoustic phases (e.g. earthquakes). Similarly, T phases are also recorded at land stations after a (back-) conversion of H phases at, e.g. island flanks or continental shelfs.

### Natural Sources

#### Earthquakes

Most of the earthquakes observed worldwide occur at plate boundaries, at both collision zones (subduction zones) and spreading centres (mid-ocean ridges), of which many are located beneath the oceans. A large proportion of earthquakes are observed on global monitoring networks on land; however, about 70% of the Earth’s surface is covered by water and thus is not part of these monitoring networks (Webb 1998). Therefore, it is difficult to detect and localize small submarine earthquakes using land-based seismic networks alone (Yun et al. 2009). Hydroacoustic stations such as those installed for the CTBT verification (e.g. Guilbert et al. 2005; Hanson and Bowman, 2005a), and short-period stations for studying seismicity at, e.g. mid-ocean ridges (e.g. Fox et al. 2001; Smith et al. 2003) are used to detect seismic waves generated by submarine earthquakes. Regional as well as global hydroacoustic stations therefore provide significant improvements in the detection accuracy and threshold (e.g. Hanson and Bowman 2005a; Yun et al. 2009).

At hydrophones, mainly T waves are registered (Guilbert et al. 2005). They are generated by the coupling of seismic waves with the water column at the seafloor before they are trapped in the SOFAR channel and propagate in this waveguide at the speed of sound in water (1.5 km/s; e.g. Williams et al. 2006). The exact processes of coupling are not fully understood and there are many theories regarding the excitation mechanisms of T phases (Tolstoy and Bohnenstiehl 2006). The conversion of seismic to T waves depends on bathymetry, but scattering also plays an important role (Guilbert et al. 2005). A detailed overview of different possible T wave generating processes is given in Tolstoy and Bohnenstiehl (2006). Illustrations of various coupling mechanisms for T phase generation are presented in Williams et al. (2006). Because of the efficient transmission of T waves in the SOFAR channel, many small earthquakes are detected at great distances (Fox et al. 2001). Seismic activity at spreading centres using multiple autonomous hydrophone moorings and also IMS hydrophone stations has been studied in Fox et al. (2001), Smith et al. (2003), and Hanson and Bowman (2005a). Graeber and Piserchia (2004) examine T waves observed at one IMS hydrophone station in the Indian Ocean and showed that mainly seismic energy from P and Pn waves coupled into the SOFAR channel and generated observable T waves.

**Fig. 3:**
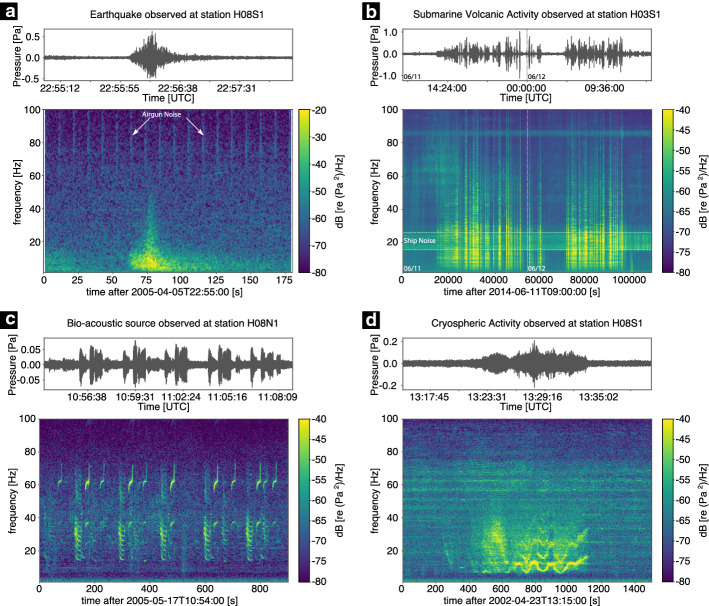
Examples of recorded hydroacoustic waveforms and associated spectrograms from four natural sources at stations of the IMS hydroacoustic network. The waveform and spectrogram of an mb 4.7 earthquake in northern Sumatra, Indonesia, recorded at hydrophone H08S1 of hydrophone station HA08 in the Indian Ocean are shown in **a**. **b** shows the waveform and spectrogram of submarine volcanic activity at Monowai, Kermadec Arc, Southwest Pacific Ocean, recorded at hydrophone H03S1 of hydrophone station HA03 in the Pacific Ocean. Between 18 and 25 Hz the spectrogram also shows ship noise (white dotted lines). The record of a bio-acoustic source, most likely whale vocalizations, recorded at hydrophone H08N1 of hydrophone station HA08 in the Indian Ocean is shown in **c**. The waveform and associated spectrogram of an iceberg event recorded at hydrophone H08S1 of hydrophone station HA08 is shown in **d**. The waveforms of **a**, **c**, and **d** are bandpass filtered applying a Butterworth bandpass filter between 1 and 120 Hz, the lower cut-off frequency for the volcanic signals (**b**) is set to 2 Hz. The spectrograms are calculated with 90% overlapping hanning windows with a length of 2^9^ (**a**), 2^15^ (**b**), 2^10^ (**c**), or 2^10^ (**d**) samples, respectively . For more information, see Table 2 (Appendix)

T waves show energy in the frequency range between 1 and 100 Hz (Tolstoy and Bohnenstiehl 2006). In the far field, the main energy of hydroacoustic earthquake waves is in the range between 2 and 8 Hz (Hanson and Bowman 2005a). The signal is characterized by an emergent onset and usually has a lens-like shape (Williams et al. 2006). Depending on their source region, the signals have different characteristics. While signals from deep-sea plains tend to be symmetric, signals from subduction zones tend to show lower frequencies with multiple peaks (Tolstoy and Bohnenstiehl 2006). The amplitudes of T waves show no dependence on the water depth of the event, but decrease with increase in distance from the source (cylindrical attenuation due to geometric spreading, 1/distance; Dziak et al. 2012). Figure 3a shows an example of an earthquake recorded at the hydroacoustic station H08S1 (Chagos Archipelago, UK, Indian Ocean). The earthquake had a body wave magnitude of mb 4.7 and the epicentre was located at 1.07° N, 97.4° E in northern Sumatra (Indonesia) according to the United States Geological Survey (USGS). Repeating high-frequency signals of short duration are seen in the spectrogram and are related to Airgun noise (see 4.3.2).

Although the observation of T waves has a great benefit in terms of detection threshold, some disadvantages result from the complexity of the coupling mechanisms and the propagation medium. For example, phase information is lost and earthquake characteristics such as magnitudes, focal mechanisms, and hypocentres are difficult to derive. In addition, the source location from T wave analysis does not represent the seismic hypocentre, but rather the location where seismic energy couples into the water column (Williams et al. 2006). Furthermore, large bathymetric features such as seamounts can obstruct the propagation path of the T wave from the source to the receiver (Fox et al. 2001). Williams et al. (2006) examine the relationship between T phase location and earthquake epicentre to draw conclusions on the source mechanisms from T wave characteristics. They characterize T waves by amplitude, onset time (time interval between first emergence to first peak), and rise time (time interval between first emergence to maximum peak) and list possible relationships with the seismic source.

#### Submarine Volcanoes

Most of the Earth’s volcanic activity (75–80%) occurs beneath the sea at mid-ocean ridges and subduction zones. Because of these locations, little is known about the spatio-temporal distribution of eruptions and other volcanic events and a lack of widespread geophysical sensors in these regions makes it difficult to observe volcanic related events, thus many go unnoticed (e.g. Dziak et al. 2012; Tepp et al. 2019). A useful tool to observe submarine volcanism are hydroacoustic signals that couple into the SOFAR channel and are recorded at large distances (Caplan-Auerbach et al. 2017; Metz et al. 2018). The first hydroacoustic observation of underwater volcanism was made by Dietz and Sheehy (1954) at a distance of about 400 km. Since then, hydroacoustic methods have been used to record seismic activity associated with submarine volcanic eruptions throughout all ocean basins (Dziak and Fox 2002; Tepp and Dziak 2021).

Summaries about submarine volcanism investigated using hydroacoustic recordings are given in Norris and Johnson (1967) and Tepp and Dziak (2021). Fox et al. (2001) use autonomous hydrophone moorings for long-term monitoring of seismic activity on the East Pacific Rise, observing tremors and earthquakes associated with volcanic activity. Metz et al. (2016, 2018) examine recordings at a hydrophone array of the IMS network over a 3.5-year period for signals related to activity at Monowai, identifying several episodes of volcanic activity and classifying Monowai as one of the most active submarine volcanoes in the world, as well as the predominant source of low-frequency sound in the central region of the Tonga-Kermadec arc. It is also the largest distance (16,000 km) at which hydroacoustic signals of volcanic origin have been detected. Talandier et al. (2020) compare the South Sarigan explosion with man-made underwater explosions and identify similarities based on, e.g. signal duration, pulse character, inverse dispersal of frequency. Caplan-Auerbach et al. (2017) describe the world’s first real time recordings of an entire eruption cycle at Axial Seamount in 2015, and also classify a new category of signals defined as submarine Hawaiian explosive activity. Dziak et al. (2012) use a long-period hydroacoustic record to calculate explosive gas flux during an eruption of a submarine volcano.

The same processes as for subaerial volcanoes also play a role in generating seismicity of submarine volcanoes (e.g. Talandier and Okal 1987; Dziak and Fox 2002). These include earthquakes (volcano-tectonic, low-frequency), various eruptive processes such as explosive eruptions and lava extrusions/flows, and mass wasting events (landslides, debris flows), as well as different types of tremor (e.g. Metz et al. 2018). An overview of the relevant processes can be found in the review by Tepp and Dziak (2021). Because of the many mechanisms involved, a variety of seismoacoustic signals with different characteristics are produced, some of which are difficult to distinguish from other events. The continuous or repetitive nature of a volcanic eruption is one criterion by which it can be distinguished from an artificial explosion (Norris and Johnson 1967).

In general, explosions and earthquakes tend to be characterized by broadband signals containing higher frequencies, while signals due to fluid movement and oscillations have lower frequencies and can exhibit a harmonic behaviour (e.g. Metz and Grevemeyer 2018). Earthquakes show frequencies in the range up to 50 Hz (Bohnenstiehl et al. 2013). Explosive eruptions are characterized by signals of short duration of tens of seconds and a broadband frequency range up to 100 Hz and more (e.g. Green et al. 2013). Commonly, signals exhibit impulsive properties (i.e. a rapid rise and sharp end) and multiple similar signals occur in clusters with short intervals (e.g. Norris and Johnson 1967; Green et al. 2013; Metz and Grevemeyer 2018). Rotian eruptions, probable the submarine equivalent to Strombolian eruptions, show repetitive pulses up to several minutes in duration with increase in amplitude and sharp cut-offs. The pulses are either broadband or narrow-banded and the frequency can vary between pulses. This eruption type is related to cyclic gas built up and escape (Tepp and Dziak 2021). Submarine Hawaiian explosions exhibit broadband (1–100 Hz), long (2 min–1 h) diffuse signals (Caplan-Auerbach et al. 2017). Bohnenstiehl et al. (2013) have observed violent explosions associated with interactions of rising magma with seawater, characterized by impulsive signals. Tepp et al. (2019) have also observed low-frequency dispersion in recordings of explosions at long distances.

Tremor signals are continuous, harmonic low-frequency signals (< 20 Hz, partly also < 40 Hz) of long duration with an emergent signal onset (e.g. Dziak et al. 2012, 2005; Tepp et al. 2019). Dominant frequencies often show a spectral shift, i.e. variation with time (e.g. Dziak and Fox 2002; Dziak et al. 2005). For example, Green et al. (2013) observed 4 and 10 Hz as dominant frequencies, while Metz and Grevemeyer (2018) identified tremor with peaks at 6 and 16 Hz, and Dziak and Fox (2002) showed tremor with a fundamental frequency of 10 Hz and harmonics at 20, 30, and 40 Hz. In contrast, Metz et al. (2018) barely detected narrowband harmonic tremor for Monowai. The recordings examined by Norris and Johnson (1967) and Bohnenstiehl et al. (2013) also include low-frequency rumbling (1–20 Hz). Lava flows and extrusions can produce both impulsive signals and tremors. Due to their usually low energy, they are only detectable on local scales and difficult to identify at regional and global distances (Tepp and Dziak 2021). An example of submarine volcanic activity recorded at hydroacoustic station H03S1 (Juan Fernández Island, Chile, Pacific Ocean) is shown in Fig. 3b. The submarine volcanic activity origins at Monowai, Kermadec Arc, Southwest Pacific Ocean (e.g. Metz et al. 2016).

#### Bioacoustic Sources

Biological sound sources are characterized by seasonal patterns in detection, are often not continuous, and exhibit frequency and amplitude modulations and variations (Sousa and Harris 2015). Marine organisms, especially marine mammals such as various species of whales and dolphins, produce acoustic signals over a wide range of frequencies. The signals vary in their characteristics from species to species and in some cases they show unique characteristics between individuals (e.g. Nieukirk et al. 2004; Dunn and Hernandez 2009), which may allow tracking over long distances in the future (Le Bras et al. 2016).

Sound produced by marine organisms varies in the frequency range from 20 Hz to several hundred kHz and is used for communication, social interaction as well as for navigation, mating, and sensing (Hildebrand 2009). Fish produce sound in the range of 50 to over 2000 Hz, both as individuals and in groups (Cato and McCauley 2002). Broadband clicks produced by dolphins have peak frequencies of about 120 kHz (Bottlenose and White beaked dolphins) and vary in duration. A tabular overview of dolphin and whale signal parameters can be found in Rasmussen et al. (2002). The sounds of various whale species have been recorded on hydrophones across all ocean basins up to a distance of 200 km (e.g. Sousa and Harris 2015; Le Bras et al. 2016). Kuna and Nábělek (2021) used sounds emitted by fin whales to derive information about sediment and crustal properties. They show that in addition to the signal in the water, reflections and refractions of these signals at various interfaces are also recorded.

Blue whales emit low-frequency (10–100 Hz), long-duration, repetitive calls with fundamental frequencies in the range of 17–20 Hz (e.g. Andrew et al. 2002; Dunn and Hernandez 2009; Le Bras et al. 2016). The calls of fin whales are in the same frequency range but show a series of sweeping pulses with regular spacing (Andrew et al. 2002; Nieukirk et al. 2004). Minke whales produce pulsing sounds with a central frequency of 30 Hz and a spacing of about 1 s (Nieukirk et al. 2004). The humpback whale is known for its unique songs. The sound of these whales in the frequency range from 20 to 2500 Hz consists of complicated sequences of different patterns (Cato and McCauley 2002; Rossi-Santos 2015). The clicking sounds of sperm whales are of a higher frequency (1–10 kHz; Cato and McCauley 2002). Sousa and Harris (2015) observe two unidentified bioacoustic signals, the so-called Diego Garcia Downsweep (DGD) with a mean frequency range of 19.3–45.0 Hz and a mean duration of 36.5 s and the Diego Garcia Croak (DGC) with a mean frequency range of 16.9–49.6 Hz and mean signal duration of 13.1 s.

Figure 3c gives an example for a recorded bio-acoustic source, which represents most likely whale vocalizations, highlighting the distinct waveform and spectral characteristics as well as the highly repetitive nature of these signals.

#### Seismic Events Related to the Ice Masses

Processes associated with icebergs such as breakup and disintegration generate hydroacoustic signals that are observed primarily in the Southern Hemisphere and contribute significantly to background noise in the Indian Ocean (e.g. Evers et al. 2013; Chapp et al. 2005; Matsumoto et al. 2014). This is mainly due to the fact that, on the one hand, most hydroacoustic stations are located in the southern oceans and, on the other hand, that there are less land masses in the southern compared to the northern hemisphere and thus less unblocked propagation paths. In addition, there are significantly larger ice masses in the southern hemisphere and a larger number of icebergs as described by Matsumoto et al. (2014) who state that the mean annual iceberg flux from Antarctica is at least two times greater than that from Greenland. Hydroacoustic signals generated by icebergs, also referred to as “cryosignals”, are very diverse and can be divided into two groups based on their signal and spectral characteristics. The first group shows rather monochromatic signals, whereas the second group shows a broader spectrum with a number of preferred frequencies. The signals of both groups can be further subdivided into individual classes. An overview of the respective classes and their properties as well as examples of spectra is found in Talandier et al. (2006). Chapp et al. (2005) name the first group as “variable harmonic tremor” (VHT) and the second as “cusped pulse tremor” (CPT). VHT are polychromatic signals with single strong spectral peaks that fluctuate with time. The fundamental frequency lies between 4 and 10 Hz, and energy is present up to more than 80 Hz. The signals can last between 1 and 30 min and are characterized by abrupt onset and termination. CPT show short single pulses with approximately equal inter-pulse intervals. The duration of a pulse with a following interval is usually 25–90 s and signals with several consecutive pulses can last from 10 min to over an hour. CPT signals show energy in the frequency range from 4 to 80 Hz and, like VHT, exhibit fundamental frequencies between 4 and 10 Hz. Both signal groups resemble signals from submarine volcanoes (Chapp et al. 2005). All signals are generated by exceptional events, i.e. they originate from iceberg breakup, disintegration, or collision. Group one originates from near the Antarctic coastline, and group two originates from icebergs drifting on high seas (Talandier et al. 2006). The harmonic nature of both signals suggests a resonance mechanism within the ice, but may also be generated by scraping of the ice mass across the shelf (Tolstoy et al. 2004b; Chapp et al. 2005). Hanson and Bowman (2005a) observe transient and impulsive signals from the Antarctic coast due to cracking noises within the ice. Glowacki et al. (2015) and Köhler et al. (2019) have investigated hydroacoustic signals related to iceberg calving events in Spitsbergen. Glowacki et al. (2015) apply high-frequency underwater ambient noise recordings in shallow water (10–45 m) to correlate the recorded acoustic energy with ice impact energy from calving events at a marine-terminating glacier. They found energy with higher frequencies (> 1 kHz) for typical subaerial events, frequencies between 10 and 1000 Hz for sliding events, and frequencies below 100 Hz for submarine events. Köhler et al. (2019) combine both seismic and hydroacoustic observations to estimate volumes of individual calving events. With hydrophones deployed in shallow water (45 m), they observe signals in a frequency range below 15 Hz and link observed multiple arrivals and weak signals after the main calving related signal to ice avalanched, air bubble pulse and ice break up in the calved ice block. An example of cryospheric activities, mostly related to icebergs in the Antarctic, recorded at hydrophone station H08S1 in the Indian Ocean is given in Fig. 3d.

### Other Natural Sources of Hydroacoustic Waves

#### Tsunami

Tsunamis are water waves that can propagate over very large distances. They are mainly excited by strong seaquakes, but also by submarine landslides and volcanic eruptions. When generated by earthquakes, the water column is set in motion due to a vertical movement of the sea floor. Tsunamis are not pressure or sound waves, but are classified as gravity waves because gravity acts as restoring force on the motion of the water. They have very long wavelengths in the order of 10 to 500 km (e.g. Heron et al. 2008; Yu 2014) and their propagation speed depends on the water depth; for example, the theoretical speed at a water depth of 5000 m is about 8000 km/h. In deep water, the vertical displacement and thus the amplitude is small, but becomes larger with decreasing water depth (Hanson et al. 2007a). In comparison, the wavelength decreases with decreasing water depth.

The low-frequency oceanic gravity wave causes pressure fluctuations that can be registered on hydrophones, although these are often not designed for the range typical of tsunamis (Hanson and Bowman 2005b; Okal et al. 2007). The typical frequency band for tsunamis registered at hydrophones ranges from 0.14 to 8 mHz, but depends on parameters of the inducing source as well as the water depth (Hanson et al. 2007a). In the raw hydroacoustic data, tsunami signals are masked by background noise and are therefore not detectable. The signals become visible by adjusting the response in the low-frequency range and by deconvolution (Matsumoto et al. 2016a, b).

Signals from the 2004 Sumatra tsunami were recorded at hydrophone stations of the IMS network in the Indian Ocean (Hanson and Bowman 2005b; Okal et al. 2007). Matsumoto et al. (2016a) describe the signals of the tsunami of the 2011 Tohoku earthquake (11 March 2011) observed on the hydrophones at the IMS hydroacoustic station HA11 (Wake Island). Matsumoto et al (2016b) analyse the acoustic signals of the tsunami triggered by the 2015 Chile earthquake. In all studies, high energy is visible in the frequency range from below 1 mHz to 25 mHz and the tsunami signals exhibit strong dispersion (e.g. Hanson et al. 2007a; Okal et al. 2007). Hanson and Bowman (2005b) were also able to observe signals from tsunami waves reflected from coastlines.

#### Submarine Landslides

Submarine landslides occur in certain areas that are particularly vulnerable to slope failures, including fjords, deltas, canyons, volcanic islands, and the open continental slope (Lee 2005). Signals from marine landslides at volcanoes have been observed in hydroacoustic data, sometimes at great distances (e.g. Caplan-Auerbach and Duennebier 2001; Caplan-Auerbach et al. 2001, 2014; Chadwick et al. 2012). Synolakis et al. (2002) examine the characteristics of T waves from the Papua New Guinea landslide (17 July 1998) and the associated tsunami recorded on hydrophones in the Pacific Ocean.

Landslides are recognizable and distinguishable from other events in hydroacoustic data and associated spectrograms because of their distinctive characteristics. The signals often begin with a low-frequency “rumble” (< 50 Hz) with an emergent signal onset, followed by a broadband coda (1–3000 Hz) lasting from a few ten seconds to minutes, sometimes hours, which is also referred to as “hiss” (e.g. Caplan-Auerbach and Duennebier 2001). The “rumble” is presumably caused by the failure of a large block, while the “hiss” is due to the downslope movement of the material. Examples of typical spectra can be seen in Caplan-Auerbach and Duennebier (2001) and in Chadwick et al. (2012). A prominent feature in the spectra are horizontal frequency bands that form due to interference between direct and reflected arrivals. The spacing between these bands changes with time due to the moving nature of the landslide as a signal source (Caplan-Auerbach et al. 2001, 2014).

#### Noise by Weather and Waves

Many other natural processes have an influence on the noise level in the ocean, especially waves and wind, but also currents, rain, or surf. The spectra are spatially as well as temporally variable (e.g. Li 1981).

Rain generates noise in the range between 5 and 20 kHz; the impact of spray increases the noise level between 1 and 1000 Hz (Prosperetti 1988). Surf breaking is observed in the range between 0.5 and 10 Hz (McCreery et al. 1993); Babcock et al. (1994) suggest a relationship of very low frequencies (< 20 mHz) with surf. Air bubbles, generated by biological activity, rain, and breaking waves, play an important role in ocean noise, evident in the frequency range up to 200 Hz, where they amplify pressure oscillations due to water turbulence. In the kilohertz range, air bubbles can themselves significantly increase the noise level through oscillations (Prosperetti 1988).

Noise generated by wind can be found in the entire range between 1 and 125 Hz. There are several theories that attempt to explain the generation of this low-frequency noise by surface wind. An overview of these is given in Nichols and Bradley (2013). According to Li (1981), the source mechanisms can be divided into four groups: wind turbulence, interactions between ocean surface waves, interaction of surface waves with oceanic turbulence, and spray and bubbles. A dependence of noise on wind in the range between 1 and at least 500 Hz is observed, but in both Chapman and Price (2011) and Duennebier et al. (2012), an increased wind influence on the noise level is only found above 100 Hz. In the 1–20 Hz range, large-scale ocean turbulence appears to play a role. Above 25 Hz, the source is in the agitation of the entire ocean surface, and a correlation with wind speeds and wave heights is observed (Li 1981). An influence of wind or wind-generated waves on the entire spectrum between 0.4 and 80 Hz has also been discovered by Duennebier et al. (2012). A comparison with wind speeds shows an increase of the noise level with increase in wind speed. Between 0.4 Hz and 6 Hz, a saturation occurs, the so-called Holu Spectrum (McCreery et al. 1993). Increased wind speeds between 7 and 10 m/s lead to the onset of wave breaking and consequently to increased noise levels above a few 100 Hz (Prosperetti 1988). Most notable is the correlation of the highest energy levels with microseism peaks (0.1 Hz and 0.16–0.3 Hz, respectively) due to surface wind wave interaction (Longuet-Higgins 1950; Hasselmann 1963; Babcock et al. 1994). Using cross-correlation of noise spectra in the microseism band (0.1–1 Hz) from the Atlantic, Ball et al. (2016) identify temporal amplitude variations within a year and dominant source regions associated with the main microseismic excitation mechanisms and sources.

In the very low-frequency range, between 20 and 100 mHz, currents and turbulences play a role (Babcock et al. 1994). Ugalde et al. (2019) observe monochromatic tremor with peaks between 5.5 and 7 Hz and spectral gliding, which is probably related to bottom water currents.

### Anthropogenic Sources

#### Underwater Explosions (Nuclear and Chemical)

Numerous studies address the characteristics of underwater explosions detected on hydrophones (e.g. Munk et al. 1988; Hanson et al. 2007b; Bowman et al. 2005b; Prior et al. 2011). Explosions, both nuclear and chemical, produce a sound wave that can propagate long distances in water within the SOFAR channel (e.g. Weston 1960; Munk et al. 1988), so that signals from small explosions (yield < 40 kg TNT) can be detected on hydrophones at distances up to 16,000 km (Prior et al. 2011). Note that explosions are mostly conducted during local day time, rarely at night (Hanson et al. 2007b).

In the case of an explosion, a spherically symmetric shock wave propagates through the water, which is characterized by an instantaneous increase followed by an exponential decay of the pressure. In addition, the high pressure creates an expanding gas bubble, which contracts due to hydrostatic pressure and starts a damped oscillation. During each minimum, the bubble emits a pressure pulse, whose amplitude decreases with time. A detailed description of the signal generation can be found, e.g. in Weston (1960). Bubble oscillation is only possible if the explosion occurs at sufficient water depth, otherwise the bubble breaks at the water surface (Heyburn et al. 2018). The bubble pulses produce peaks and troughs in the amplitude spectrum caused by interference of the initial pulse with the subsequent bubble pulses (e.g. Reymond et al. 2003; Hanson et al. 2007b). Both the spacing of the peaks and the bubble pulse delay time (time between the initial pulse and the first bubble pulse) depend on charge weight, water, and detonation depth (Heyburn et al. 2018). Typical bubble pulse delay times are 0.1–0.5 s (Hanson et al. 2007b).

**Fig. 4:**
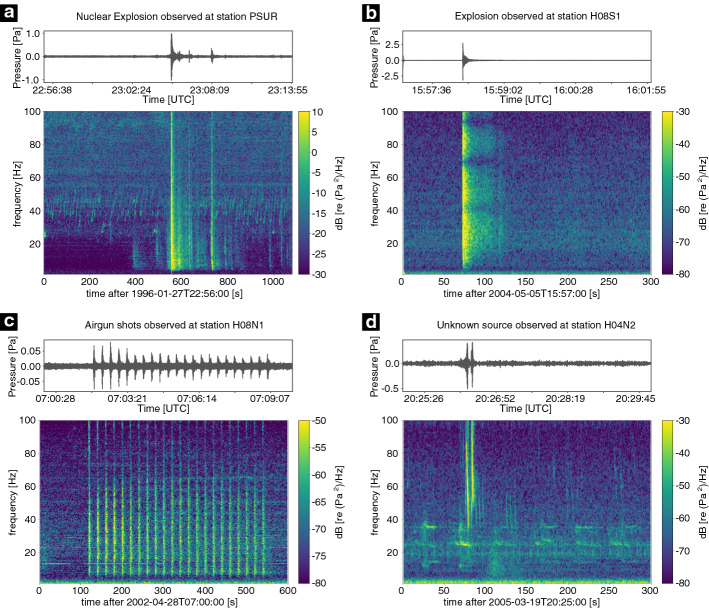
Examples of recorded hydroacoustic waveforms and associated spectrograms from four anthropogenic sources at stations of the IMS hydroacoustic network (**b**, **c**, **d**) and at the hydroacoustic station Point-Sur (PSUR), California (**a**). The waveform and spectrogram of the last French nuclear test conducted in 1996 near Muroroa, Fangataufa Atoll, South Pacific, are shown in **a**. **b** shows the waveform and spectrogram of an accidental under-water explosion in the Bay of Bengal recorded at hydrophone H08S1 of hydrophone station HA08 in the Indian Ocean. The record of an airgun survey conducted in the Indian Ocean recorded at hydrophone H08N1 is displayed in **c**. The waveform and associated spectrogram of an unknown source, probably a submarine SONAR, recorded at hydrophone H04N2 of hydrophone station HA04 in the southern Indian Ocean is shown in **d**. The waveforms are bandpass filtered applying a Butterworth filter between 1 and 120 Hz. The spectrograms are calculated with 90% overlapping hanning windows with a length of 2^10^ (**a**), 2^9^ (**b**), 2^10^ (**c**), or 2^9^ (**d**) samples, respectively. For more information, see Table 2 (Appendix)

The frequency content of underwater explosions depends on the size and depth of the detonation (e.g. Munk et al. 1988). The larger the yield, the smaller the generated and recorded frequencies (Bowman et al. 2005b). The signals are usually broadband with frequencies ranging from 2 Hz to at least 80 Hz, and frequencies as high as 125 Hz have also been observed. The majority of energy is usually below 20 Hz (e.g. Milne 1959; Munk et al. 1988). Signals from explosions exhibit dispersive properties: at first, the signals contain mainly low-frequency components (5–10 Hz), while high frequencies up to 80 Hz dominate in the later part of the signal (Prior et al. 2012). The typical properties are also seen in the examples given in Figs. 4a and b, showing the waveforms as well as the associated spectrograms of the last French nuclear test (Fig. 4a) conducted in 1996 near Muroroa, Fangataufa Atoll, South Pacific, recorded the hydrophone station Point-Sur (PSUR), California, and an accidental underwater explosion (Fig. 4b) in the Bay of Bengal recorded at hydrophone H08S1 of hydrophone station HA08 in the Pacific Ocean.

The signals from explosions usually show an increase in amplitude until a peak, followed by a sharp cut-off (crescendo shape). This is typical of signal propagation in the SOFAR channel (Prior et al. 2011, 2012). The signals are of short duration compared to earthquake signals (e.g. Milne 1959; Okal 2001; Hanson et al. 2007b) and after the initial pulse, the oscillations of the gas bubble are often seen as further pulses (e.g. Reymond et al. 2003; Hanson et al. 2007b; Adushkin et al. 2004). Changes in signal shape usually result from interaction with bathymetry such as seamounts or reflections along the path (Blackman et al. 2003; Prior et al. 2011).

An example of the observations of nuclear explosions can be found in Milne (1959). Hanson et al. (2007b) detect more than 300 explosions of various origins (military exercises, demolition and construction, blast fishing) on hydrophone data over a 6-year period. Heyburn et al. (2018) analyse hydroacoustic signals from three underwater chemical explosions as part of US Navy shock tests near Florida observed at IMS hydrophone stations and elsewhere. Prior et al. (2012) describe the signals of an accidental explosion recorded at an IMS hydrophone station at up to 8000 km distance during the Shallow Water 2006 experiment. Bowman et al. (2005b) investigate two events in the Bay of Bengal that were identified as explosions based on their high-frequency content and spectral scalloping. Adushkin et al. (2004) present the results of a study of observed seismic, acoustic, and hydroacoustic effects at distances of up to 30 km from underwater explosions of various intensities in a lake. The latest and so far strongest (i.e. sixth) nuclear weapons test by the Democratic People’s Republic of Korea (DPRK6, September 2017) was studied by Nielsen et al. (2018). They use P phases that travelled through the subsurface to the coast before they were converted to T phases and recorded at distant hydroacoustic stations to localize the event. Compared to the localization using 125 land-based seismic IMS stations, the explosion was localized 27 km further to the south when using the registrations at six hydrophone triplets of the IMS hydrophone stations alone.

A different example of an explosion is the Argentine submarine ARA (Armada de la República Argentina) San Juan. Hydroacoustic data from two IMS hydrophone stations were used to investigate the disappearance of this submarine in November 2017 as unusual hydroacoustic signals were recorded by two stations at a distance of 6000 to 8000 km and localized near the submarine’s last known position shortly after the last contact with the submarine. The unusual signal shows an isolated pulse-like event of short duration (10 s), with short rise time, dispersion, and broadband frequency content between 1 and 80 Hz with a dominant frequency between 7 and 10 Hz (Nielsen et al. 2020; Vergoz et al. 2021). Vergoz et al. (2021) were able to show that the signals are two impulsive sources within a short time interval. Using a calibration blast at the last known position of the submarine, it was shown that signals from this area can be detected at the hydroacoustic stations (Nielsen et al. 2020; Vergoz et al. 2021). In this case, the dominant frequency of the calibration event is higher (15–20 Hz) and the amplitude is lower compared to the signals related to the submarine. Vergoz et al. (2021) infer successive implosions of the two compartments of the submarine from the characteristics of the hydroacoustic signals, but the exact source mechanism cannot be determined from the signals. The submarine was found on 17 November 2018, exactly one year and two days after its disappearance in a water depth of about 900 m around 55 km from its last known position and less than 20 km apart from the location of the hydroacoustic anomaly provided by the CTBTO (Nielsen et al. 2020). By additionally using several reflected hydroacoustic arrivals, the source location accuracy of the anomalous hydroacoustic event associated with the implosion of the ARA San Juan could be significantly improved, giving an estimated location 3.5 km from the known location of the wreckage (Vergoz et al. 2021).

#### Controlled Marine Sources for Exploration and Research and Sonar

A variety of active/controlled marine seismic sources is used for different applications. High-frequency sources such as chirp systems (500 Hz < f < 200 kHz), boomers and sparkers (50 Hz < f < 4000 Hz) offer a high resolution, while low-frequency sources like airguns and waterguns (20 Hz < f < 1500 Hz) allow a greater depth of investigation. Because of effective transmission, seismic exploration signals are among the main sources for elevated ambient sound levels in the oceans (e.g. Nieukirk et al. 2004; Hildebrandt 2009; Miksis-Olds and Nichols 2016). Wiggins et al. (2016) describe airgun signals as a constant dominant noise source in the frequency range between 10 and 100 Hz in the Gulf of Mexico. Tolstoy et al. (2004a) and Breitzke et al. (2008) show the results of broadband source calibration for airgun configurations as used for academic research to obtain information about emitted sound levels. Mougenot et al. (2017) describe various developments on marine vibrator sources that belong to the continuous, non-impulsive sources and offer the advantage of improved control over the source signature as well as signal repeatability.

*Airguns* In both industry and academia, airguns are the main seismic source to study the marine subsurface because the signals are controllable, predictable, and repeatable (Breitzke et al. 2008; Bohnenstiehl et al. 2012; Chelminski et al. 2019; Landrø and Amundsen 2010). There are several airgun types that differ in volume (0.5–60 l), operational pressure (140–200 Pa), and tow depth (6–10 m). The recorded sound level depends on frequency and distance of the source (Breitzke et al. 2008; Wiggins et al. 2016). Airguns are generally used in arrays, which provides advantages on the signal properties in amplifying the impulsive nature of the signal (Mougenot et al 2017; Chelminski et al. 2019). The arrays are usually arranged to generate a downward pulse, but there are also configurations that result in horizontal radiation (Nieukirk et al. 2004; Breitzke et al. 2008; Bohnenstiehl et al. 2012).

Airguns generate acoustic signals similar to those of underwater explosions by the explosive release of high-pressure air into the surrounding water (e.g. Hutchinson and Detrick 1984; Dragoset 2000). This produces a sharp initial pulse followed by secondary pulses generated by oscillations of the air bubble under hydrostatic pressure as it rises to the water surface (Hutchinson and Detrick 1984). These oscillations, also called bubble oscillations, produce short, broadband signals of decreasing amplitude (Hutchinson and Detrick 1984; Nieukirk et al. 2004). For arrays, the source signature is characterized by a stronger initial peak with a short rise time and much smaller bubble oscillations (Chelminski et al. 2019). In addition to volume or pressure, the reflection coefficient of the sea surface (Krail 2010) or the near-source bathymetry (Bohnenstiehl et al. 2012) has an influence on the signal shape, which therefore shows a variable character. In this context, airgun shots in shallower water depths are more likely to produce pulse-like signals of shorter duration and higher amplitude than in deep water (Bohnenstiehl et al. 2012). An overview of typical characteristics of airguns and airgun arrays is given in Breitzke et al. (2008).

Airgun signals show energy in the frequency range below 200 Hz (Nieukirk et al. 2004; Bohnenstiehl et al. 2012), mostly in the frequency band between 10 and 100 Hz (Tolstoy et al. 2004a; Miksis-Olds and Nichols 2016). The frequency content and bandwidth as well as the signal amplitude are affected by volume, pressure, and tow depth. A larger volume generates more low-frequency components (Breitzke et al. 2008). Airgun signals are effectively transmitted in the ocean over long distances due to their frequency content and have been observed at distances larger than 3000 km on hydrophones deployed to monitor seismic activity along mid-ocean ridges (Nieukirk et al. 2004; Bohnenstiehl et al. 2012). Figure 4c shows an example of an airgun survey recorded at a hydroacoustic station H08N1 in the Indian Ocean. Note the high similarity of the single pulses and the broad frequency content. Blackman et al. (2004) conclude from their experimental observations that airguns are capable of generating sufficient energy in the range between 5 and 60 Hz that can propagate through ocean basins for thousands of kilometres and Le Bras et al. (2019) demonstrate that airgun signals could also be measured up to a distance of more than 15,000 km. In this regard, airguns operated in shallow water can generate signals that get trapped in the sound channel over long distances (Bohnenstiehl et al. 2012). In long-range observations, the signals are characterized by a high degree of regular repetition, typically every 10–20 s, over longer periods of time (hours to days/months) and are distinguishable from biogenic sources due to their low-frequency content (Nieukirk et al. 2004).

*Water guns* So-called water guns generate a hydroacoustic signal through the implosion of a cavity, which is created by injecting a water jet at high velocity into the surrounding water. Due to the velocity contrast and the pressure difference between the cavity and the surrounding pressure, the cavity collapses, creating a strong impulsive shock wave (Landrø et al. 1993). In comparison with an airgun of a comparable size, a watergun generates a shorter, bubble-free broadband signal (Hutchinson and Detrick 1984). The fraction of generated high frequencies is larger for waterguns, while the low-frequency fractions are reduced. Unlike other marine sources, the signal from a watergun is not a minimum-phase wavelet, which has to be taken into account during processing.

*Signal Underwater Sound Charges (SUS) and Imploding Glass Spheres* Blackman et al. (2003, 2004) describe a series of hydroacoustic calibration shots in the Indian Ocean recorded at IMS stations, where in addition to an airgun array, signal underwater sound charges (SUS) and imploding glass spheres were used. The latter uses glass sphere and a piston driven fragmentation system that causes the sphere to break at a predetermined depth. The sphere generates a signal with an initial, relatively low-frequency pulse corresponding to the influx of water caused by the failure of the glass container. Convergence at the centre of the sphere generates a shock wave, creating a spike that dominates the recording followed by a small bubble pulse. If a multiple sphere system is used, the shock wave of the first implosion is superimposed and followed by the implosion shock waves of the other spheres. The signals of the glass spheres have a frequency content between 300 and 500 Hz in the near field and between 40 and 125 Hz in the far field. Underwater explosions for scientific purposes are usually generated by SUS charges. These are small explosive charges that produce a chemical explosion (e.g. Chapman 1985; 1988; Blackman et al. 2003). Hydrophone recordings show energy in the range between 40 and 120 Hz, with some weak signals in the range between 0 and 40 Hz. Both SUS and glass sphere signals could be detected over long distances (Blackman et al. 2003, 2004).

*Sonar* There are different types of sonar (Sound Navigation and Ranging) that emit hydroacoustic waves in different frequency ranges. A detailed overview of the characteristics of different systems can be found in Hildebrand (2009). Low-frequency active sonars with a frequency range of 100 to 500 Hz and mid-frequency sonars (1–8 kHz) are used by the military to detect submarines and emit energy primarily in the horizontal direction. Figure 4d shows a signal that can probably be associated with a submarine SONAR, recorded at the hydrophone station H04N2 (Crozet Islands, France) in the southern Indian Ocean. Commercial and civilian sonars used for navigation, detection of underwater targets, and mapping of the ocean floor (echosounding sonars, single beam sonar, multibeam sonar, subbottom profilers) produce hydroacoustic signals in the range of a few kHz to 100 kHz that tend to be directed toward the ocean floor.

### Other Anthropogenic Sources of Hydroacoustic Waves

Anthropogenically generated noise is mostly of low frequency, typically in the range below 100 Hz (Reine et al. 2014). In addition to commercial shipping, industrial drilling and dredging generate acoustically detectable noise. Dredging is used, among other areas, in harbours and waterways close to the shore to maintain and deepen the navigation channel. Drilling is mainly used in hydrocarbon exploration and production, but also in scientific applications. Other sources in this field are offshore wind farm construction and operation or geotechnical site investigations (Erbe and McPherson 2017). The frequency ranges of different sources observed in various studies are summarized in a table in Todd et al. (2020). While many studies focus on sound levels of various processes in shallow water and nearshore areas (e.g. Dickerson et al. 2001; Blackwell et al. 2004; Blackwell and Greene 2006; Reine et al. 2012), there are also a few studies addressing deeper water (e.g. Jimenez-Arranz et al. 2019; Todd et al. 2020). The various processes can often be identified by distinct frequency characteristics.

#### Ship Traffic

Various authors have observed an increase in ambient noise levels in the frequency range between 5 and 100 Hz in the Pacific and Indian Oceans over the last decades, which is largely attributed to an increase in ship traffic (e.g. Andrew et al. 2002; Blackman et al. 2002; Harben and Hauk 2010; Miksis-Olds and Nichols 2016). In addition to cargo ships in commercial shipping, supply vessels, drill ships and rigs, seismic and transport vessels, and icebreakers also insonify the oceans. Acoustic sounds from ships are primarily found in the frequency range between 10 and 1000 Hz and can be transmitted and detected over long distances (e.g. Reine et al. 2014; Sadaf et al. 2015; Wiggins et al. 2016). The sound is primarily caused by cavitation of air bubbles due to propeller rotation (e.g. McKenna et al. 2012). Therefore, low-frequency spectral bands (e.g. 8 Hz, 17.5 Hz, and 60 Hz) and their harmonics are often detected in the frequency spectrum as a prominent feature that can be attributed to the propeller’s blade speed (Curtis et al. 1999; Blackwell and Greene 2006; Baumgartner et al. 2018). Metz et al. (2016) show that ship traffic appears in the form of a continuous, quiet noise band in the range of 18 to 25 Hz (Fig. b).

Ugalde et al. (2019) identify closely passing ships in hydrophone and OBS data. The high-frequency signals show multiple harmonics and last for several hours. Cosens and Dueck (1993) study the sounds of icebreakers in the Arctic. Commercial cargo vessel such as container ships and tankers emit primarily hydroacoustic signals below 40 Hz and bulk carriers emit signals with a frequency of about 100 Hz (McKenna et al. 2012). The time–frequency spectrum exhibits a U-shape as the ships pass the sensor. Chapman and Price (2011) show a correlation between noise and major shipping routes by calculating a horizontal directionality from the noise.

#### Drilling, Dredging, Oil Exploration and Production

Blackwell et al. (2004) describe and characterize the underwater sound levels, frequency ranges, and distance dependence of various sources from the Northstar drilling platform during drilling and oil production. They observed different peaks with high sound levels. Frequencies between 125 and 160 Hz are associated with oil production and the observed peak at 1 kHz is caused by machinery. In general, the highest acoustic energy is observed between 60 and 250 Hz and between 650 and 1400 Hz during winter, whereas during the summer months the highest observed frequencies are in the range between 30 and 100 Hz (Blackwell and Greene 2006). Dickerson et al. (2001) investigate various underwater sounds generated by bucket dredging operations. The individual processes are examined in terms of frequency content and sound level. The main part of energy is measured in the range between 20 and 1000 Hz and can be detected up to about 10 km. Lower frequencies could not be recorded in this example due to the instruments used. Reine et al. (2012, 2014) characterize underwater sounds from a backhoe dredge and describe in detail the sound level characteristics of individual processes. The most common signal observed is sound from the engine or generator (with a peak at 400 Hz). Other processes show peaks at 315 Hz (bottom impact/grabs), 630 Hz (hydraulic ram sounds), 250 Hz (pop noise), 100 Hz and 500 Hz (both barge loading sounds), and around 1200 Hz (anchoring spud sounds).

The main part of the energy of different drilling processes is found in the range between 40 and 400 Hz (Erbe and McPherson 2017). Geotechnical drilling emits less energy than, for example, oil production drilling, as the latter uses much larger platforms. Besides the drilling process itself, support vessels and helicopters also contribute to the underwater noise field. Todd et al. (2020) show frequency spectra for various drilling processes of a jack-up exploration drilling rig on the Dogger Bank (North Sea). Significant sound pressure levels were observed in the range between 2 and 1400 Hz, with drilling noise mostly below 10 Hz. Jimenez-Arranz et al. (2019) map the sound field during normal production of a mobile offshore drilling unit in the North Atlantic, where mainly low-frequency energy in the range below 250 Hz is emitted.

#### Offshore Wind Turbines

Offshore wind turbines are a source of underwater noise not only during operation, but already during construction, where pile-driving generates underwater sounds by the impact of the hammer on the pile that is transmitted through both the water and the subsurface (e.g. Madsen et al. 2006; Norro et al. 2011). These signals are impulsive and occur at relatively constant intervals of 1 to 4 s over an extended period of time (e.g. Amaral et al. 2020). They usually show an oscillatory decrease after the pulse-like onset (Nedwell et al. 2007). For pile driving, Betke et al. (2004) measure frequencies between 100 and 300 Hz and Madsen et al. (2006) measure frequencies below 500 Hz. Matuschek and Betke (2009) show an example waveform and spectra for pile driving with the maximum energy in the range between 100 and 400 Hz. The frequency content depends on the distance to the source. For example, Nedwell et al. (2007) show that the spectra at a distance of 10 km contain significantly lower frequencies (100 Hz to > 100 kHz) than at a distance of 100 m.

During operation, offshore wind turbines generate underwater sound by the rotation of the rotor blades, which is transmitted to the water through the tower and foundation (e.g. Betke et al. 2004; Norro et al. 2011). The underwater sound level during operation is highly variable, as has been described by, e.g. Norro et al. (2011) and Yang et al. (2018). The sound level and the frequencies depend on the distance to the source, wind speed, water depth, type and number of wind turbines, rotation speed, and the subsurface properties (e.g. Degn 2000; Madsen et al. 2006). In general, the sound generated during operation is continuous, has tonal components and frequencies below 1000 Hz, and a lower sound level than passing vessels at the same distance (e.g. Norro et al. 2011). Yang et al. (2018) show that there is a correlation between the measured underwater acoustic data and wind speed-dependent turbine vibrations (30–500 Hz) and conclude that the underwater sound is generated by mechanical vibrations of the tower. Norro et al. (2011) measure the highest sound pressure level between 150 and 300 Hz and Betke et al. (2004) observe peaks between 60 and 250 Hz. A table with frequency ranges and sound levels compiled for different studies can be found in Tougaard et al. (2020). Degn (2000) has also observed increased noise levels below 50 Hz due to wave impact against the structure. Most offshore wind turbines are installed in relatively shallow waters (e.g. in Germany: 10–50 m), so the propagation of sound is dominated by reflections, which results in a rapid decrease in amplitude, making long-range detection difficult (Madsen et al. 2006).

#### Tidal Stream Turbines

The sound emitted by a tidal stream turbine under water has been studied by Risch et al. (2020). The main energy of the generated sound is between 50 and 1000 Hz. The signal is tonal with an oscillating fundamental frequency around 100 Hz and a bandwidth of 25 to 75 Hz. Harmonics were observed up to 2000 Hz. The signal could be detected above the ambient noise up to 2000 m away from the turbine during calm seas.

#### Underwater Loudspeaker

Many underwater loudspeakers are based on sound generation mechanisms that are not capable of producing low-frequency sound. Furthermore, they are often limited in their use to shallow water depths. Due to these limitations, Fonseca and Alves (2012) developed a new reliable underwater sound source capable of generating sound with high accuracy in the frequency range between 10 and 3000 Hz. The system includes an underwater sound generator and the corresponding electronic driver. The sound is generated by a rigid plate actuated by purely electromagnetic forces.

## Infrasonic Sources

Pressure fluctuations with a frequency below the human hearing threshold of 20 Hz are referred to as infrasound. The lower-frequency limit for infrasound is defined at the Brunt-Väisälä frequency, which corresponds to the natural frequency of vertical oscillations in a stably stratified atmosphere due to gravity and buoyancy as the restoring forces. Between the acoustic cut-off frequency and the Brunt-Väisälä frequency acoustic waves transit to acoustic-gravity and gravity waves, while air pressure is replaced by gravity as the restoring force. Both characteristic frequencies are in the range of 10^–2^ to 10^–3^ Hz, depending on the temperature and humidity. A variety of natural sources generates infrasound, including earthquakes, volcanic eruptions, auroras, and meteoroids, as well as lightning. In addition, a large number of anthropogenic sources also emit infrasound, e.g. explosions (chemical, nuclear) and mining activities, rocket launches and satellite re-entries into the atmosphere, as well as wind turbines and other man-made machinery. Descriptions of different natural and man-made sources in terms of signal characteristics, generation mechanisms, and theoretical background information are given by Blanc (1985) and Bedard and Georges (2000). Very large explosions such as strong volcanic eruptions and large objects entering the Earth’s atmosphere can cause both infrasound and gravity waves (e.g. Ripepe et al. 2010). Furthermore, there is a correlation between the detection of gravity waves and lightning activity, the latter of which is particularly associated with infrasound observations in the tropics (e.g. Farges et al. 2021). Blanc et al. (2010) have observed atmospheric gravity waves due to thunderstorm cloud convection and a detailed description of gravity waves from thunderstorms is given by Blanc et al. (2014).

Infrasonic waves propagate in the atmosphere and their propagation is influenced by atmospheric conditions (e.g. Arrowsmith et al. 2008; Shani-Kadmiel et al. 2018). Waveguides in the atmosphere, which are created by wind and temperature gradients, and the resulting stratification of the atmosphere enable the propagation and detection of infrasound at large distances (e.g. Bowman and Lees, 2017; Shani-Kadmiel et al. 2021). Similar to the SOFAR channel in the oceans, acoustic energy can be trapped in three dominant atmospheric waveguides: the tropospheric waveguide below an altitude of maximum 16 km, and the stratospheric and thermospheric waveguides, which extend to altitudes of 40 to 55 km and 110 to 160 km, respectively. For a description of these waveguides, we refer to, e.g. Drob et al. (2003).

Like seismic waves, the registered infrasonic signals are divided into phases depending on the waveguide they propagate through, or on the refracting layer. The phases are distinguished on the one hand by their trace velocity and on the other hand by the quotient of epicentral distance and travel time, called celerity (e.g. Ceranna et al. 2009; Vergoz et al. 2019). Signals that have been refracted in the troposphere (tropospheric phase) are labelled “Iw” and usually have a trace velocity in the range of the speed of sound (~ 330 m/s) with typical celerity values around 340 m/s (e.g. Ceranna et al. 2009; Vergoz et al. 2019). Infrasonic waves trapped between the stratopause and the ground are called stratospheric phases (“Is”). Their trace velocities are about 360 m/s and the celerity ranges between 280 and 310 m/s with an average of 300 m/s (e.g. Le Pichon et al. 2006). Waves propagating between the ground and the lower thermosphere are termed thermospheric phases (“It”). They have a trace velocity larger than 350 m/s and are characterized by a low celerity of 250 to 280 m/s. However, due to strong attenuation in the upper atmosphere, these phases are not always observed, particularly not at large distances (e.g. Ceranna et al. 2009; Vergoz et al. 2019). In addition, multiple signals, i.e. signals that have been reflected several times between a boundary layer and the ground, can also occur; these are then assigned a subsequent number (e.g. It2).

Moreover, the wind direction in the atmosphere plays an important role with regard to the propagation and the detectability of infrasound, which results in a directionality of observations (e.g. Assink et al. 2016). Especially the detection of stratospheric phases correlates with the seasonal variation of the prevailing stratospheric wind direction. Due to the strong winds in the stratosphere, these phases can propagate over large distances.

### Natural Sources

#### Meteoroids

Fragments of a comet or asteroid are called meteoroid, very bright meteoroids are called bolides or fireballs (e.g. Silber and Brown 2019). Upon entering the Earth’s atmosphere, large meteoroids generate infrasound that can propagate throughout the Earth’s atmosphere because of its low-frequency properties as well as refraction and channelling in atmospheric waveguides (e.g., Arrowsmith et al. 2007b; Ens et al. 2012; Le Pichon et al. 2013). Infrasonic signals can be used to determine the energy radiated by meteoroids (Silber et al. 2018), as well as their location and trajectory (e.g. Brown et al. 2002; Pilger et al. 2020). Empirical relations between signal parameters and properties of the meteoroid, especially the yield, have been established by ReVelle (1995), Ens et al. (2012), or Silber and Brown (2019), among others. Arrowsmith et al. (2007b) use infrasound signals from three large bolides recorded at numerous infrasound stations of the IMS to present a method for differentiating these signals from other large global events. The detection capability of the IMS at short and long range was investigated by Pilger et al. (2015) using the Chelyabinsk meteorite. De Groot-Hedlin and Hedlin (2014) use the same event to investigate the long-range propagation of infrasound and the source characteristics.

There are different mechanisms involved in the infrasound generation from meteoroids and a detailed description of the theoretical background can be found in Silber and Brown (2019). Meteoroids travel at a velocity which is significantly larger than the speed of sound (e.g. Ens et al. 2012). Upon entering the atmosphere, the meteoroid is decelerated and loses some of its kinetic energy (Le Pichon et al. 2002b), creating a shock wave that results in pressure waves in the infrasonic range propagating approximately perpendicular to the path of the meteoroid (Le Pichon et al. 2002b; Ens et al. 2012). Despite the loss of energy, the body still passes through the atmosphere at supersonic speeds and can be considered as a very fast-moving point source (e.g. Brown et al. 2002; Le Pichon et al. 2013) that also generates a strong shock wave (Silber et al. 2018). This mechanism can be well approximated by a cylindrical line-source explosion (e.g. Ens et al. 2012; Pilger et al. 2015, 2020).

The strongest infrasound signal is usually generated by the fragmentation or explosion of the meteoroid caused by heating due to friction in the atmosphere (e.g. Le Pichon et al. 2013; Pilger et al. 2020). This process resembles a point source and the infrasound propagates quasi omnidirectional (Ens et al. 2012; Pilger et al. 2020). In most cases, the explosion occurs in the upper atmosphere (70–110 km altitude), but larger objects may also fragment at lower altitudes (Le Pichon et al. 2002b). In addition to these sources, the impact of the meteorite at the solid Earth generates an infrasound signal (Ens et al. 2012; Silber et al. 2018), but this is observed only in very rare cases.

The typical frequency range observed for meteoroid events lies between 0.2 and 3 Hz (Ens et al. 2012). The lowest observed signal frequencies are around 0.01 Hz (e.g. Le Pichon et al. 2013), while ReVelle et al. (2004) observed signals with frequencies significantly larger than 1 Hz. Le Pichon et al. (2013) record signals with dominant frequencies between 0.01 and 0.05 Hz. For the Chelyabinsk event, Pilger et al. (2015) observe frequencies at about 0.025 Hz and lower. The frequency range reported in Brown et al. (2002) is between 0.1 and 0.35 Hz, while the main energy in Le Pichon et al. (2002b) was found between 0.3 and 0.8 Hz. The different observed frequency ranges partly result from different properties of the meteoroid itself or its trajectory and partly from the different propagation paths through a spatio-temporally variable atmosphere (Le Pichon et al. 2013). In general, larger meteoroids/events emit lower frequencies (e.g. ReVelle 1995; Silber et al. 2018; Silber and Brown 2019) and signals with a dominant frequency lower than 0.05 Hz suggest high explosion energy (Le Pichon et al. 2013).

**Fig. 5:**
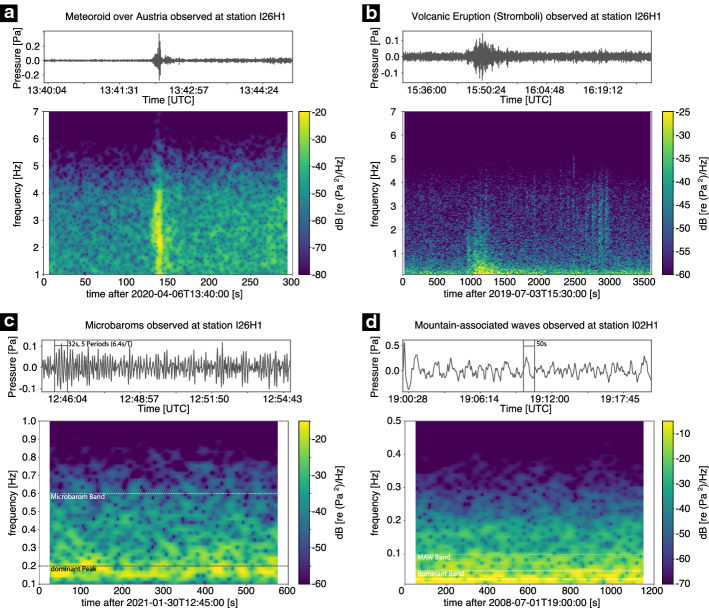
Examples of recorded infrasonic waveforms and associated spectrograms for selected natural sources at stations of the IMS infrasonic network. The waveform and spectrogram of a meteoroid close to Salzburg, Austria, recorded at element I26H1 of the infrasound array IS26 in south-eastern Germany, are shown in **a**. **b** shows the waveform and spectrogram of the July, 3rd 2019 Stromboli eruption recorded at element I26H1 of the infrasound array IS26. The record of microbaroms from the Atlantic Ocean, recorded at element I26H1 of the infrasound array IS26 is displayed in **c**, the upper frequency of the microbaroms band is marked by a white dotted line, and the grey dotted line marks the dominant peak of 0.2 Hz. The waveform and associated spectrogram of mountain-associated waves (MAW) in the Andes, South America, recorded at element I02H1 of the infrasound array IS02 in southern Argentina is shown in **d**. The MAW band is marked by a white dotted line, the dominant band between 0.025 and 0.05 Hz is marked by a white dashed line. For both the microbaroms and MAW signals, the typical period is marked in the waveform panel. The waveforms are bandpass filtered applying a Butterworth bandpass filter between 1 and 4 Hz (**a**), 0.1 and 4 Hz (**b**), 0.1 and 0.6 Hz (**c**), or 0.01 and 0.1 Hz (**d**). The spectrograms are calculated with either 99% (**a**, **c**, **d**) or 90% (**b**) overlapping hanning windows with a length of 2^8^ (**a**), 2^10^ (**b**), 2^10^ (**c**), or 2^11^ (**d**) samples, respectively. For more information, see Table 2 (Appendix)

The observed waveforms are highly variable in their length, polarity, amplitude as well as onset behaviour (e.g. Le Pichon et al. 2002b; De Groot-Hedlin and Hedlin 2014). In addition, signals registered at longer distances from the source are subject to modifications due to atmospheric conditions along the propagation path, e.g. the amplitude shows a dependence on the prevailing winds in the atmosphere (e.g. Ens et al. 2012). The shock waves generate signals with the N-shape typical for sonic booms and short (1–10 s) signal duration. This particular signal shape can be observed up to a distance of 300 km (Silber and Brown 2014), because the influence of the atmosphere on the signal modification is rather small within this distance (Ens et al. 2012). In some cases, several successive N-shaped pulses are also registered. Thereafter, increasingly more signal shape changes occur caused by the influence of atmospheric properties along the propagation path (e.g. Silber and Brown 2014; Silber et al. 2018). Signal durations ranging from several seconds (e.g. ca. 50 s; Le Pichon et al. 2002b), to several minutes (10–15 min; Brown et al. 2002; Arrowsmith et al. 2007b), to several hours (2–3 h; Le Pichon et al. 2013) are observed. At longer distances, the signals show an emergent onset and a slow decay (e.g. Le Pichon et al. 2013; Silber and Brown, 2014). This is interpreted as a superposition of signals along multiple propagation paths, but may also be produced by the spatio-temporal extension of the source (Le Pichon et al. 2013). Figure 5a shows the waveform of a meteoroid over Austria recorded at the IMS infrasound station IS26 in Germany.

#### Volcanic Eruptions

Volcanism is highly variable and spans a spectrum of processes ranging from effusive to explosive behaviour. As a result, volcanic processes generate a variety of low-frequency acoustic signals that vary in both duration and frequency content and that are associated with different source mechanisms (Matoza and Fee, 2018; Matoza et al. 2019). Explosive volcanic eruptions are among the strongest sources of infrasound observed on Earth (Cannata et al. 2011; Matoza et al. 2017). As the majority of the energy of a volcanic eruption is released into the atmosphere, infrasound is a suitable technology that can be utilized to monitor volcanoes (Campus 2006). Because of efficient ducting in atmospheric waveguides and low attenuation, the signals are detectable in distances of several thousand kilometres, making infrasound the only ground-based monitoring technique for volcanoes applicable at very large distances (e.g. Cannata et al. 2011; Marchetti et al. 2019). For near-source measurements (< 5 km; Lacanna and Ripepe 2013), the atmosphere has little effect on the recorded signal because it is relatively homogeneous and isotropic within this distance (Perttu et al. 2020). Therefore, signals measured in the near-field represent the source processes at the volcano (Johnson and Ripepe 2011) and can be used to characterize eruption properties as well as to determine pressure and material release into the atmosphere (e.g. Fee and Matoza 2013; Perttu et al. 2020). Low-frequency (< 0.5 Hz) infrasound signals correlate with ash cloud properties such as height and lateral extent (e.g. Fee et al. 2010, 2013).

Processes that generate infrasound include explosions, various eruption types (fountaining, strombolian, volcanic, subplinian, plinian), and rockfalls, as well as pyroclastic density and mudflows. As infrasound is generated only when the source processes are coupled with the atmosphere (e.g. during an explosive eruption), most sources are found near the surface or are aerial. Many are related to the surface release of volcanic gases, which can produce both explosions and long-lasting vibrations, i.e. tremors (Fee and Matoza 2013; Ripepe et al. 2018), but internal magma dynamics also play a role in generating the signals (Cannata et al. 2011). However, the mechanisms behind these processes are diverse and some are not understood in detail.

The infrasound generated by volcanoes has a wide spectral bandwidth, with most signals falling in the range between 0.5 Hz and 4 Hz (Campus 2006; Fee et al. 2010). Due to atmospheric absorption, the frequency content depends on the propagation distance; infrasound signals measured at larger distances contain a lower proportion of high frequencies (e.g. Fee et al. 2010; McNutt et al. 2013). In addition, frequency differences are also evident in signals recorded in different directions (e.g. Le Pichon et al. 2005a; Green et al. 2012; Perttu et al. 2020). The waveforms of the individual source processes show differences, but they can also vary for the same source on a temporal scale (e.g. Cannata et al. 2011). The signal length ranges from a few seconds to several months and the amplitudes span a wide dynamic range (e.g. Matoza and Fee 2018). However, waveform characteristics not only depend on source processes such as the yield (i.e. the strength of the eruption), but also show variability with the distance of the measurements from the event and atmospheric wind conditions (e.g. Fee et al. 2010, 2013; Campus 2006; De Angelis et al. 2012). The amplitude of long-range recordings is sensitive to diurnal variations in wind speed (Green et al. 2012); the duration of the signal increases with distance due to, e.g. multi-pathing (Fee et al. 2013), and passage through a caustic changes the signal phase (Fee et al. 2013). Complex propagation effects as well as topography and crater morphology result in complicated waveforms (Fee et al. 2010; Matoza and Fee 2018). For on overview of waveforms and spectra for individual eruption and signal types, we refer to Johnson and Ripepe (2011) and Matoza et al. (2019).

Infrasonic tremor signals are continuous pressure oscillations that can last for a very long time (up to several months; Fee and Matoza, 2013). There are both monochromatic and harmonic tremors. The former have a single peak in the spectrum, while the latter are characterized by a fundamental frequency and associated harmonics. If the frequency varies with time, it is also referred to as “gliding” tremor. In addition, broadband and spasmodic (variable amplitude) tremors are observed (Fee and Matoza 2013). The sources of tremor are as diverse as the signal characteristics: besides oscillation and bursting of gas bubbles, resonances in magma, effusive eruptions, various degassing processes, and roiling lava lakes are also possible generating mechanisms (e.g. Matoza et al. 2010; Johnson and Ripepe 2011; Matoza and Fee 2018). Tremor-like signals with broadband spectra are produced by fountains from low-level fissure eruptions (Fee and Matoza 2013), which also often exhibits signal characteristics similar to explosive signals with an impulsive, positive first onset. Observed frequencies are above 2 Hz (Cannata et al. 2011).

Explosions exhibit a variety of signal shapes and lengths, but are mostly abrupt and short duration events characterized by a compression and subsequent decompression of similar amplitude followed by a short coda of a few seconds to minutes (Fee and Matoza 2013). They result from a sudden release of pressure, which generates a shock wave (Le Pichon et al. 2005a). These pulses often have N shapes (Matoza et al. 2011; Fee et al. 2013) and are a common feature of explosive eruptions (Fee et al. 2010; Johnson and Ripepe 2011). Most signals of explosive nature display a frequency content in the range between 0.075 and 2 Hz (Fee et al. 2013). Strombolian activity is characterized by one or more impulses of short duration with a compressional onset, followed by a decompression signal of longer duration (Fee et al. 2013; Fee and Matoza 2013). According to the strombolian bubble vibration model, the infrasound signal is generated by the vibration of a thin magma layer due to pressure fluctuations within a shallow bubble (Cannata et al. 2011).

In the context of Vulcanian eruptions, Fee et al. (2013) observed emergent signal onsets that can probably be explained by degassing processes during magma ascent. Most often, however, this type of eruption exhibits an impulsive onset with a short signal duration, generated by the interactions between water and magma, the failure of a “lava plug” sealing a conduit, or the coalescence of large amounts of gas (Fee et al. 2013). Vulcanian eruptions are similar in signal shape to those of explosions and Strombolian eruptions. In some cases, very strong eruptions can produce a shock wave resulting in the characteristic N-shape of the signal (Fee and Matoza 2013). Vulcanian-Plinian eruptions are characterized by a superposition of short impulsive, explosive signals and broadband tremor (Matoza et al. 2011 and references therein). For Plinian eruptions, Fee et al. (2010) observed signal frequencies ranging from 0.25 Hz to less than 0.1 Hz. Subplinian eruptions typically exhibit sustained signals (several minutes to hours) with a broadband spectrum (Fee et al. 2013; Fee and Matoza 2013). A peculiarity observed mainly during long-lasting Vulcanian and Plinian eruptions is the similarity of some signals of large amplitude and long duration to jet noise. This signal shape is associated with jet flow within the eruption column (Matoza et al. 2009, 2017; Fee and Matoza 2013).

Volcanic activities further generate long period signals related to shallow long-period seismicity and degassing processes (Fee and Matoza 2013), as well as ultra-long period signals (~ 50–230 s, mean ~ 120 s; 0.008 Hz) associated with the rise and oscillations of an ash plume (Fee et al. 2013; Fee and Matoza 2013). Acoustic-gravity waves (period 200 s–300 s; 0.003–0.005 Hz) are thought to result from excitation of the atmosphere by large amounts of thermal energy generated during strong volcanic eruptions (Kanamori 2004; Fee et al. 2013). Examples are the eruptions of Krakatau (1883), Agung (1963) or Mount St. Helens (1980), where global atmospheric oscillations with frequencies in the millihertz range could be observed (Johnson and Ripepe 2011). Additionally, both large and moderate volcanic eruptions can also generate gravity waves with periods ranging from 300 s to several minutes, i.e. frequencies < 0.003 Hz (Fee and Matoza 2013), which have been observed during the eruption of Okmok Volcano (2008, Alaska) or the eruption of Mount Pinatubo (1991, Philippines; e.g. Kanamori 2004).

By means of recorded infrasound signals, the eruption histories of remote volcanoes can be reconstructed (e.g. Matoza et al. 2011) and based on the correlations of observed signals with different eruption processes, e.g. Ripepe et al. (2018), Marchetti et al. (2019), and Matoza et al. (2019) propose infrasound as a tool for automated and near-real-time early warning and reporting systems on regional and global scales. Figure 5b gives an example of a waveform recorded at the infrasound array IS26 from an explosive eruption of Mt. Stromboli, Italy. The same signal is seen as converted ground motion in the seismic record in Fig. 1b.

#### Microbaroms

A continuously oscillating pressure signal, known as microbaroms, is detected at infrasound stations worldwide (e.g. Landès et al. 2012; De Carlo et al. 2020). Microbaroms are characterized by quasi-monochromatic continuous wave trains in the frequency range between 0.1 Hz and 0.6 Hz (e.g. Hupe et al. 2019a; Sindelárová et al. 2021) and usually show a peak around 0.2 Hz, which corresponds to twice the dominant ocean surface wave frequency of 0.1 Hz (e.g. Hetzer et al. 2008). This signal, like microseisms, is ocean-generated noise, and is thus the acoustic equivalent of microseismicity, exhibiting a very similar signal shape and frequency range, suggesting a common source mechanism (e.g. Landès et al. 2012; Bowman and Lees 2017).

Microbaroms result from second-order nonlinear interactions of ocean surface waves (wind waves) of the same frequency propagating in opposite directions (e.g. Bowman and Lees 2017; De Carlo et al. 2021). This is consistent with the theories of Longuet-Higgins (1950) and Hasselmann (1963) for the origin of microseisms (Posmentier 1967). On the one hand, microbaroms are generated by surface motion compressing the overlying air and, on the other hand, by ocean radiation (e.g. Landès et al. 2014). They propagate in the atmosphere, especially in the stratosphere (e.g. De Carlo et al. 2020), and because of low absorption and effective ducting between the stratosphere and the ground, microbaroms can be detected at distances of several thousand kilometres from the source (e.g. Hupe et al. 2019a). Yet, propagation and detection depend on the atmospheric conditions along the propagation path and are strongly influenced by the prevailing (stratospheric) winds (Bowman and Lees 2017). For signal detections in the Northern Hemisphere, the source regions are located in the North Atlantic, North Pacific and Indian Oceans. In the Southern Hemisphere, signals are mainly detected from the Antarctic Circumpolar Current (Bowman and Lees 2017; De Carlo et al. 2021). There are clear seasonal trends in the identified source regions as well as in the signal strength, which are largely influenced by the stratospheric wind and temperature conditions (e.g. Landès et al. 2012, 2014). According to Landès et al. (2014), the source regions and their variability follow the global atmospheric circulation model and the north–south variations of ocean-storm activities in both hemispheres. The correlation is particularly evident at mid-latitudes, where tropospheric and stratospheric winds influence both the source regions and the propagation of microbaroms (De Carlo et al. 2021).

De Carlo et al. (2020) provide an overview of the mathematical background of individual generation mechanisms of microbaroms, reviewing the unified microbarom source theories developed by Brekhovskikh et al. (1973) and Waxler et al. (2007). De Carlo et al. (2021) show the first quantitative validation of global microbarom modelling based on ocean wave models, a new source model, and atmospheric attenuation. Hupe et al. (2019a) model microbarom amplitudes and the direction of arrivals at the German infrasound station IS26 using an operational ocean wave interaction model and a semi-empirical attenuation relation to model the source and signal amplitude, respectively, to better understand seasonal variations in the characteristics (e.g. amplitude, direction of arrival) of the microbarom detections. The spatio-temporal variations of microbarom detections at a global scale were modelled by Landès et al. (2014) combining the source term resulting from the nonlinear ocean wave interaction and a simplified description of the long-range infrasound propagation through the stratospheric waveguide, and the results were compared with observations at infrasound stations. An example recording of microbaroms originating over the Atlantic Ocean and registered at the infrasound array IS26 in Germany is given in Fig. 5c, showing the highest energy inside the typical dominant frequency range.

#### Mountain-Associated Waves (MAW)

Mountain-associated waves (MAW) are an infrasonic phenomenon originating from regions with high mountains or mountain chains. Cook (1969) first observe such signals in North America during the 1960s and linked them to mountainous regions as a result of triangulation. The IMS infrasound network allowed to determine the major global source regions of MAWs (Blanc et al. 2018; Ceranna et al. 2019; Hupe et al. 2019b), among which are the southern Andes as well as high mountain ranges in Central Asia, in New Zealand, and North America.

The exact source mechanism of MAWs has not fully been solved yet. In addition to topography, the meteorological conditions were considered relevant not only for the propagation of MAWs (Rockway et al. 1974), but also and particularly for the excitation. Larson et al. (1971) found a correlation between the MAW occurrence as well as amplitude and tropospheric wind conditions, particularly of mountain chain crosswinds and proposed complex feedback mechanisms of the acoustic energy reinforcing the sound-producing flow, such as ground and terrain reflection or turbulent flows when surrounding obstacles such as mountain peaks. Similarly, Chimonas (1977) propose that vortex shedding at mountain peaks led to acoustic emission when wind oscillations are scattered to acoustic modes. Chunchuzov (1994) propose that strong wind gusts within turbulent flows near mountains produced acoustic impulses superimposing to signals that can be detected at remote instruments. Since Chunchuzov’s model enabled to reproduce observed amplitudes of MAWs, he reinforced the theory of turbulence being involved in the process. Hupe et al. (2019b) conclude that crosswinds were not sufficient to fully explain the seasonality in MAW detections at different stations, with a maximum occurrence during the winter. They hypothesized that breaking gravity waves at different altitudes played a role in the MAW excitation and Blanc et al. (2018) already demonstrated the matching source regions of both phenomena.

MAWs have frequencies between 0.01 and 0.1 Hz and typical durations of minutes to several hours. The dominant periods observed vary from 20 to 40 s (Bedard 1978) or up to 80 s (Larson et al. 1971). The waveform characteristics of the detected events depended on the distance from the source terrain, as more distant mountain ranges apparently resulted in lower frequencies at the sensors than nearer sources. MAWs have also been measured by infrasound sensors on a balloon when crossing the southern Andes (Poler et al. 2020). An example waveform recording and its associated spectrogram is shown in Fig. 5d, showing mountain waves recorded at IMS station IS02 in southern Argentina. A typical period of 50 s is seen in the waveform plot and the signal shows the highest energy in the typical MAW band below 0.1 Hz.

### Other Natural Sources of Infrasonic Waves

#### Severe Storms, Lightning, Tornados

Phenomena that occur during severe weather generate low-frequency pressure variations (infrasound and atmospheric gravity waves; e.g. Bedard 2005). In particular, these phenomena include tornadoes (e.g. Talmadge and Waxler 2016), thunderstorms (e.g. Assink et al. 2008; Lamb et al. 2018), and hailstorms (e.g. Bowman and Bedard 1971), which are all associated with highly convective storms or cells (e.g. Georges and Greene 1975). The emitted infrasound is detected on nearby sensors, but can also be registered at long distances (e.g. Georges and Greene 1975). Since the spectra of storm types differ, these variations in radiated infrasound can potentially be used to identify the source and obtain information on mechanisms and the producing storm type (e.g. Bowman and Bedard 1971). Both Bedard (2005) and Schecter et al. (2008) observe that infrasound is primarily registered when the generating storms also produced hail. Bowman and Bedard (1971) found a relation between the dominant infrasound frequency and the diameter of the hailstones.

For strong storms, infrasound is observed in the ranges of between 0.02 and 0.08 Hz and between 0.5 and 2.5 Hz (Bowman and Bedard 1971; Bedard 2005). In between these ranges, microbaroms dominate, therefore, signals from storms are not easy to identify. A tabular overview of the properties of various phenomena based on model calculations can be found in Bedard (2005).

In thunderstorms lightning (cloud-to-ground and intracloud), produced by the mutual repulsion of charged water droplets in charged regions of the cloud, is responsible for much of the acoustic emissions, producing both acoustic waves in the audible (namely thunder) and infrasonic range (e.g. Assink et al. 2008; Farges and Blanc 2010; Arechiga et al. 2014). The main mechanism is believed to be the generation of a shock wave by the rapid thermal expansion of the lightning channel. After a lightning discharge, an acoustic rarefaction pulse is produced as atmospheric pressure equilibrium is restored (Dessler 1973; Pasko 2009). Signals from lightning are therefore single pulses with a typical N-shape, i.e. compression followed by rarefaction, observed in the frequency range between 0.1 and 10 Hz, with many observations between 0.2 and 2 Hz (e.g. Schecter et al. 2008; Lamb et al. 2018). A mechanism that explains the observed amplitude of infrasonic signals from thunder is given in Lacroix et al. (2019) which is based on the theory of Few (1969). Intracloud lightning generates at least one, but most probably two, infrasonic pulses in opposite directions (Arechiga et al. 2014). Other possible phenomena that generate infrasound during a thunderstorm are so-called transient luminous events, which include sprites (high-altitude discharges), halos, elves, and blue or gigantic jets (Liszka 2004; Farges et al. 2005; Farges and Blanc 2010). Their physical mechanisms and consequently their signal properties are diverse. Infrasonic signals from sprites have been suggested by Liszka (2004) and first detected by Farges et al. (2005). da Silva and Pasko (2014) describe possible mechanisms of infrasound generation from sprites. Infrasound signals from sprites show frequency dispersion, i.e. low frequencies are registered before higher ones, resulting in a chirp shape of the signal, which can be explained by propagation effects (dispersion) and expansion of the sprites leading to signal length of up to 150 s (Farges and Blanc 2010). A theoretical description based on numerical modelling concerning the dispersion effects observed in infrasonic signals originating from sprites is given in de Larquier and Pasko (2010).

Tornadoes emit infrasound in the range between 0.5 and 10 Hz (e.g. Schecter et al. 2008; Elbing et al. 2019; Petrin and Elbing 2019). Bowman and Bedard (1971) observe signals in the frequency range between 0.02 and 0.08 Hz for tornadic storms, and Petrin and Elbing (2019) as well as Elbing et al. (2019) observed a peak at about 8 Hz with non-harmonic overtones up to 44 Hz. The frequency content is related to the size of the tornado: the lower the observed frequency, the larger the tornado. The main source of infrasound associated with tornadoes is not yet clear (e.g. Schecter et al. 2008; Petrin and Elbing 2019). Possible mechanisms include electromagnetic sources, non-equilibrium effects, various aeroacoustic processes, latent-heat-related sources, and also vortex vibrations, but according to current research the contribution to the observed infrasonic signals from tornados from electromagnetic sources such as lightning, electrostatic charging and nonlinear pulse stretching is unlikely. (e.g. Petrin and Elbing 2019). Bedard (2005) showed that the measured infrasound signals can be explained by the radial-modes-of-vibration model of Abdullah (1966). Frazier et al. (2014) assume that vorticity is the main source of infrasound. Schecter et al. (2008) propose three-dimensional Rossby waves and adiabatic processes involving hail as the primary mechanism. In addition, vibrations, rotations, and turbulence as well as the unsteady motion of a developing or mature tornado may play a role as signal sources (Schecter et al. 2008). Since infrasonic signals are observed minutes (Frazier et al. 2014; Elbing et al. 2019) to an hour (Georges and Greene 1975) prior to the initial observation or touchdown of a tornado, infrasound is predestined as a possible technology for early warning systems.

#### Aurora

Atmospheric infrasound associated with auroral and magnetic activity is observed at high latitude infrasound stations in, e.g. Alaska, Canada, Scandinavia, and Antarctica (Wilson 1967, 2005; Wilson and Nichparenko 1967). The aurora is formed in the uppermost layers of the Earth’s atmosphere by the collision of energetic particles (mainly electrons) with ionized oxygen and nitrogen atoms. The collisions transfer kinetic energy into the atmosphere, resulting in a temperature rise, and the accompanying pressure rise triggers an acoustic wave that propagates to infrasound sensors at the Earth’s surface (e.g. Wilson et al. 2005).

There are two types of auroral infrasound. Type 1 is referred to as aurora infrasonic waves (AIW), which are generated by auroral bow waves. Type 2 are pulsating aurora infrasonic waves (PAIW) generated by pulsating auroras, which usually occur in the morning hours as very bright auroras with fluctuating brightness (Wilson 2005; Wilson et al. 2005). Auroral infrasonic waves show three characteristic features: (1) high anisotropic emission of pressure waves in the direction of auroral motion; (2) they are detected at the Earth’s surface by infrasonic sensors only when the auroral motion is at supersonic speed; and (3) the waveform resembles that of a shock wave (Wilson 1967; Wilson and Nichparenko 1967). In addition, a midnight reversal in the azimuth of arrival of the infrasound waves from east to west of the magnetic meridian is evident, corresponding to the observed midnight reversal in the direction of auroral motion (Wilson 1967). The shock wave is usually observed about 6 min after the aurora reaches the zenith (Pasko 2012).

The main infrasonic signal seems to originate from the dynamics of the moving aurora. Currently, there are two possible mechanisms involved in the generation of auroral infrasound: the Lorentz force and Joule heating during supersonic motion (Pasko 2012). Based on the assumption that only infrasound from aurorae moving at supersonic speeds is registered, and resulting from the characteristic waveform of these signals, a shock wave mechanism is postulated for the origin of auroral infrasound (e.g. Wilson 1967). AIW are associated with the supersonic motions of auroral bows, which contain strong electrojet currents that are coupled to the neutral atmosphere via the Lorentz force (Wilson 2005). The infrasound signals are generated by a bow wave created by the moving auroral element. The surface imprint of this bow wave propagates in the same direction and at the same speed as the source of the auroral arc (Wilson et al. 2005). PAIW are produced by pulsating auroras whose brightness fluctuates regularly with periods of 2 to 50 s, periodically heating the atmosphere (Wilson et al. 2005). They have a horizontal extent of 10 to 200 km and a vertical extent of 2 to 25 km and exhibit a pulse repetition period of 1 to 40 s (De Larquier et al. 2010).

Auroral infrasound signals exhibit frequencies ranging from 0.01 Hz to 0.1 Hz (e.g. Wilson and Nichparenko 1967; Wilson et al. 2005). In most cases, oscillations with irregular periods are evident. There is little difference in frequency content between AIW and PAIW, but AIW appear to have a contribution of slightly lower frequencies (Wilson et al. 2005; Pasko 2012). In general, the signals are characterized by multiple oscillations of widely varying periods and exhibit a fairly variable waveform (Wilson and Nichparenko 1967). In 10% of the cases, the signals show a signature typical of shock waves with a first positive peak, followed by a longer negative phase and an overall short signal duration (Wilson 1967, 2005). The signals from AIW exhibit an impulsive character, larger amplitudes, and appear to arrive at the stations from a high angle (~ 50°; Wilson 2005), while the waveform from PAIW is characterized by very long (several hours), continuous, quasi-periodic wave trains (Wilson et al. 2005). Another distinguishing feature between PAIW and AIW is their trace velocity: it averages 0.460 km/s for AIW (Wilson and Nichparenko 1967) and is larger than 1 km/s for PAIW (Wilson et al. 2005). More complex waveforms indicate multiple paths propagation effects, but can also result from the superposition of multiple sources (Wilson 1967).

#### Earthquakes

Earthquakes, especially strong ones (moment magnitude Mw > 6), generate seismic waves that, as they propagate across the Earth’s surface, radiate infrasonic waves into the atmosphere due to the vertical components of surface motion (e.g. Cook 1971; Hernandez et al. 2018) like the Mw 8.3 Tokachi-Oki earthquake (Japan, 25 September 2003) that produced seismic velocity amplitudes in the order of 1.2 cm/s and 0.8 cm/s and a differential pressure perturbation of 4 and 3 Pa, respectively, at seismic and infrasonic sensors of two seismoacoustic arrays in the Republic of Korea at distances of 1480 and 1550 km (Kim et al. 2004). By mechanically compressing or decompressing the atmosphere, surface motion generates an acoustic pressure wave (Shani-Kadmiel et al. 2021). Three types of signals are typically identified: (1) local infrasound, (2) epicentral infrasound, and (3) diffracted infrasound or infrasound from secondary sources (e.g. Le Pichon et al. 2005b; Che et al. 2007; Arrowsmith et al. 2009). An illustration of the three different processes is given in Arrowsmith et al. (2010). Local infrasound is a pressure change caused by the vertical displacement in the area of the infrasound sensor when seismic surface waves are passing by and are the first signals to be detected as they propagate as seismic waves with velocities larger than 2 km/s. Strong low-frequency surface motions in the epicentral region, caused by the sudden displacement of the ground, are converted to sound pressure waves (ground-coupled air waves) which propagate nearly horizontal to the Earth’s surface through the atmosphere with a mean speed of 300 m/s and are recorded as epicentral infrasound signals which arrive after the local and secondary signals. The third type is generated by the interaction of by-passing seismic surface waves with topographic features such as large mountain ranges (e.g. Le Pichon et al. 2002a) or sedimentary basins (e.g. Che et al. 2007; Marchetti et al. 2016), where seismic waves efficiently couple to atmospheric infrasound (e.g. Le Pichon et al. 2003; Hernandez et al. 2018). As these signals propagate partly through the ground and partly through the atmosphere, they are registered as the second signals (e.g. Shani-Kadmiel et al. 2018). A further distinction of the infrasonic wave types is based on the observed horizontal trace velocity. This is > 3 km/s for type 1 (local infrasound; seismic waves) and about 0.35 km/s for both type 2 (epicentral infrasound) and type 3 (secondary sources; e.g. Le Pichon et al. 2005b).

Epicentral infrasound shows frequencies in a broadband range (0.05–4.4 Hz), typically between 1 and 3 Hz (e.g. Marchetti et al. 2016; Pilger et al. 2019). Mutschlecner and Whitaker (2005) and Le Pichon et al. (2003) observed frequencies of 0.8 Hz and 0.1 Hz. As higher frequencies are attenuated by absorption, lower frequencies are observed with increase in distance from the source (Pilger et al. 2019). There are relations between the hypocentral depth and the frequency as well as the amplitude of the infrasonic signal (Mutschlecner and Whitaker, 2005). While the frequency decreases with increase in hypocentre depth, shallow seismic sources produce large amplitude ground motions and thus large amplitude infrasound (Shani-Kadmiel et al. 2018). The signal shape and amplitude depends on the distance between the sensor and the source, the propagation path, and the stratospheric wind direction (e.g. Le Pichon et al. 2006). Infrasound signals usually show emergent onset (e.g. Arrowsmith et al. 2009), can be spindle-shaped (Marchetti et al. 2016), and are of long duration (a few minutes to more than 40 min; e.g. Che et al. 2007; Pilger et al. 2019). A long-lasting signal indicates a larger source region and is often associated with secondary sources (e.g. Le Pichon et al. 2005b, 2006).

Infrasound observations allow that conclusions about the characteristics of the generating earthquake can be drawn. Mutschlecner and Whitaker (2005) analysed the properties of infrasonic signals for more than 30 earthquakes and found relations between seismic magnitude and signal amplitude and duration, respectively. Likewise, Le Pichon et al. (2006) and Shani-Kadmiel et al. (2018) observe a correlation between the signal amplitude of epicentral infrasound and the seismic magnitude, which is based on the relationship between magnitude and the strength of ground motion. The signal length is correlated with the magnitude and the seismic source mechanism (Le Pichon et al. 2006). Hernandez et al. (2018) describe infrasonic signals radiated by the Mw 6.2 Amatrice earthquake (Central Italy, 24 August 2016) and compare the acoustic surface pressure derived from infrasound records and the seismic source pressure derived from measured seismic ground motion. They find an agreement between the back projected infrasound, given in sound pressure level values (SPL) and the acoustic peak surface pressure (PSP) calculated from measured peak ground acceleration (PGA). In a distance of 130 km from the epicentre, an infrasonic signal with a maximum peak-to-peak amplitude of 5 Pa is observed. In comparison, Tinti et al. (2016) show amplitudes in the peak ground velocity between 6 and 22 cm/s at various strong motion accelerometers within a radius of about 45 km around the epicentre. Likewise, Walker et al. (2013) show a correlation between regions with high peak shaking amplitudes with high acoustic surface pressure for the Tohoku earthquake (Japan, 11 March 2011) by a back projection of infrasound signals. Similar to Kim et al. (2004) who find a correlation between local infrasound and ground velocity in both amplitude and phase, a positive correlation between the vertical seismic component (max. peak 1.9 mm/s) and infrasonic signals (max. peak 0.3 Pa) was also found by Laštovička et al. (2010) for a weak earthquake (M3.6) in the Czech Republic. Le Pichon et al. (2005b) investigate infrasound generated by large earthquakes in the Sumatra region between 2004 and 2005 and showed that ground motion near the epicentre and vibrations of nearby land masses radiate infrasound. They observed three distinct waveform signatures associated with the 2004 Sumatra–Andaman earthquake, namely seismic phases, T phases, and purely atmospheric arrivals, with the first two being observed due to the sensitivity of the sensors to ground vibrations. Pilger et al. (2019) analyse infrasonic signals recorded at large distances for a magnitude 7.5 Earthquake (28 September 2019, Indonesia) and identified source regions related to the intense shaking of both the epicentral region and nearby topography. Che et al. (2007) observe atmospheric infrasound waves at regional distances following the 16 August 2005 Miyagi-Oki (Japan) earthquake and applied a source-location procedure to construct earthquake generated infrasound source regions finding that the comparatively long duration of ground motion in a sedimentary basin could be defined as one source of infrasound related to the large earthquake. Shani-Kadmiel et al. (2021) show that infrasound signals may be used to derive acoustic shaking intensity maps (infrasound based shake maps) using the example of the Haiti earthquake (2010).

#### Mass Movements: Avalanches, Pyroclastic Flows, Rockfalls

Snow avalanches, pyroclastic flows, landslides, icefalls from glacier collapse, and rockfalls are all considered as mass movements with similar flow dynamics (Havens et al. 2014a), that represent a non-stationary, extended and complex source for infrasound (Ripepe et al. 2009; Marchetti et al. 2015).

The frequency range of infrasonic signals emitted by the different mass movement processes is proportional to the moving volume and lies between 0.4 and 10 Hz (e.g. Ripepe et al. 2009; Havens et al. 2014a). The range typically observed for avalanches is between 1 and 5 Hz (Scott et al. 2007; Marchetti et al. 2015). For pyroclastic flows, Ripepe et al. (2009) observed signals in the frequency range between 0.4 and 7 Hz. Marchetti et al. (2021) found peak frequencies of around 2.5 to 3 Hz for icefalls associated with a glacier collapse, which are superimposed on an oscillation with a frequency of 0.1 Hz that is generated by airflow around the moving avalanche mass.

The signal shapes depend on the topography of the mountain slope (Yamasato 1997), but are generally of longer duration. Rockfall generates complex signals. Moran et al. (2008) observe higher frequency impulsive signals for the initial impact and long-period infrasonic signals (50 s/0.02 Hz) for rockfall resulting from the air displacement of the falling rock. For avalanches, the waveforms are typically spindle shaped (Kogelnig et al. 2011), showing a gradually increasing amplitude followed by a longer lasting decrease in amplitude (Havens et al. 2014a; Marchetti et al. 2015). Kogelnig et al. (2011) have registered signals of consistent length (about 60–80 s), regardless of the size of the avalanche, and noted a similarity between the infrasonic and the seismic signals. Processes generating infrasound include turbulent snow-air-flow from the avalanche front (powder cloud; Kogelnig et al. 2011) and the movement of the avalanche itself, which disturb the atmospheric pressure and generate pressure waves (Havens et al. 2014b; Marchetti et al. 2021). The emitted signals are proportional to the flow velocity and the amplitudes are higher for powder avalanches than for wet avalanches (Schimmel et al. 2017). Vertical eruptions of the powder cloud generate particularly strong pulses (Havens et al. 2014b).

The signals of pyroclastic flows have a long duration. Similar to avalanches, the main source of the signals is assumed to be at the front. Initial impulsive low-amplitude signals are caused by fracturing at the onset of the lava dome collapse followed by further single impulsive signals generated by the impact of individual lava blocks against the mountain flank (Yamasato 1997). Yamasato (1997) also observed a Doppler effect, which suggests that infrasound is also generated during the movement of the pyroclastic flow.

#### Surf

Infrasonic signals associated with surf and breaking ocean waves are observed throughout the frequency range between 0.5 and 20 Hz, with the main part of the energy being concentrated in the range from 1 to 5 Hz (Garcés et al. 2003; Le Pichon et al. 2004). The waveforms are location dependent, being influenced by the coastal characteristics (rocks, shallow lava beds, etc.), the wave height, and the breaking period. Arrowsmith and Hedlin (2005) observe impulsive signals and pulse groups over longer periods of time, which are related to single breaking wave fronts and the breaking of waves in individual segments (Garcés et al. 2003). Mechanisms include individual breaking waves, the interaction of a wavefront with a whole coastline, and bubble oscillations during surf (e.g. Arrowsmith and Hedlin 2005; Aucan et al. 2006; Garcés et al. 2006; Park et al. 2008). Most processes include the release or compression/expansion of large air volumes, generating a pressure wave (Le Pichon et al. 2004). There is a correlation between the amplitude of the pressure wave and the ocean wave height (e.g. Garcés et al. 2003; Le Pichon et al. 2004; Arrowsmith and Hedlin 2005), which is particularly evident in the peak frequency of the infrasonic signal envelope (4–10 Hz), corresponding to the breaking wave period (Aucan et al. 2006; Garcés et al. 2006).

#### Cryosphere

Various processes related to interactions between the ice masses, the ocean, and the underlying surfaces such as iceberg calving and capsizing, sea ice breaking, and collisions between sea ice and icebergs generate locally detectable infrasound (e.g. Murayama et al. 2017; Podolskiy et al. 2017). Further infrasound sources are ice quakes and stick–slip motion (Preiswerk et al. 2016). Caused by the numerous processes, the signals differ in frequency content, waveform, and duration. Most signals show energy in the range between 1 and 10 Hz. Murayama et al. (2017) observe dominant frequencies between 3 and 8 Hz and a peak in the range of 1 to 5 Hz, consistent with other mass-movement events (landslides, avalanches, etc.), was noted in Podolskiy et al. (2017). Richardson et al. (2010) observe signals with frequencies well above 5 Hz and a peak between 11 and 14 Hz. Depending on the event, impulsive signals of short duration or prolonged signals may occur. Events within the continental ice sheet or the sea ice show signal lengths of 20 to 30 s (Murayama et al. 2017). In connection with the calving of glaciers and icebergs impulsive signals with a duration of less than 30 s and frequencies of 1 to 10 Hz are observed. Podolskiy et al. (2017) also observe longer calving events (several minutes) consisting of individual short pulses spaced a few seconds apart, some of which overlapped so that a continuous oscillation was recorded. Based on the short duration, Richardson et al. (2010) conclude that the signals associated with calving and fractures couple well with the atmosphere.

#### Animals

Similar to the hydroacoustic domain, there are various terrestrial animals that use infrasound for communication including elephants, rhinoceroses, tigers, giraffes, and okapis (e.g. von Muggenthaler 2000; Herbst et al. 2012). These species are known to produce, detect, and respond to infrasound signals (e.g. Garstang 2010). Elephants produce vocalisations with a frequency below 10 Hz and can presumably perceive signals down to a frequency of 1 Hz (Garstang 2010). Most sounds are in the range between 14 and 24 Hz and usually last 10 to 15 s (Payne et al. 1986). In captive elephants, Payne et al. (1986) have observed signals in clusters up to 10 min long as well as isolated calls. Rhinos produce signals in the range between 10 and 80 Hz with the infrasonic signal components being contained in sounds audible to humans (von Muggenthaler et al. 1993). The sounds vary from species to species. Sumatran rhinoceroses emit, among other calls, so-called whistle-blows, which contain a high level of infrasound (von Muggenthaler et al. 2003). Von Muggenthaler (2000) studied the vocalisations of tigers and observed fundamental frequencies of about 17.5 Hz for the roars of some tigers. Giraffes generally produce sounds in the frequency range between 20 and 40 Hz, with vocalisations found exclusively or predominantly in the infrasonic range below 20 Hz. Okapi communicate in the range between 14 and 35 Hz and produce sounds similar to elephants (von Muggenthaler 2013).

### Anthropogenic Sources

#### Nuclear and Chemical Explosions

Similar to natural sources such as volcanic eruptions, chemical and nuclear explosions, both atmospheric and underground, generate detectable infrasonic waves. The explosive events can be detected at infrasound sensors up to long distances of several hundred to thousands of kilometres depending on the yield size and the prevailing stratospheric wind conditions. Downwind from the source, the detectability is significantly better than upwind or normal to the source (e.g. Christie et al. 2005).

The primary use of the infrasound technology in the context of the CTBT is the detection of atmospheric nuclear tests, which have not been conducted for over 40 years. However, the conducted underground (nuclear) explosions in the recent decades have been found to cause detectable infrasound. This requires the near-surface explosion source to generate a sufficiently large seismic ground motion, which converts to infrasonic energy in the form of atmospheric pressure waves due to coupling between the surface and the atmosphere (Koch and Pilger, 2019). Non-nuclear explosions in recent years, such as the accidental surface explosions at the Buncefield Oil depot (UK, 2005), the Baumgarten Gas Hub (Austria, 2017), an oil refinery near Ingolstadt (Germany, 2018), and a large accidental explosion in the harbour of Beirut (Lebanon, 2020), have been studied by Ceranna et al. (2009), Schneider et al. (2018), and Fuchs et al. (2019) as well as Koch and Pilger (2020) and Pilger et al. (2021b). Other sources of explosions include mining blasts (ReVelle 2005), train car explosions (Norris et al. 2005), and munition dump explosions (Green et al. 2011). Vergoz et al. (2019) describe the detection of 180 infrasonic signals related to the explosion of the Antares 130 rocket in 2014 recorded at one IMS and several temporary infrasound stations. Other sources are surface mining explosions and quarry blasts.

Explosions are impulsive sources (e.g. Ceranna et al. 2009). Infrasound waves are generated during atmospheric explosions by overpressure and the mechanisms for underground explosions are the same as for earthquakes, leading to the observation of multiple signals from different source regions. The signals from earthquakes and explosions are distinguished based on signal duration and amplitude. The ground motion of an explosions is shorter and more impulsive compared to that of an earthquake and explosions produce signals with larger amplitude (Che et al. 2014). The observation of strong seismoacoustic signals suggests the generation of strong infrasound signals (Fuchs et al. 2019). If the amplitudes of the infrasound waves are significantly higher than those of seismic waves at co-located sensors, this suggests that the main part of the energy of the explosion has propagated through the atmosphere (Ceranna et al. 2009). The waveforms of infrasound signals generated by explosions are divers and depend on the propagation path and medium (Schneider et al. 2018; Vergoz et al. 2019). The waveforms tend to be impulsive and of short duration (e.g. Christie et al. 2005; Green et al. 2011; Che et al. 2014). Specific waveforms usually appear depending on phase, direction, and distance. Thus, symmetric “N” shaped, but also shocked and smoothed “U” shaped signals are observed (Vergoz et al. 2019). Pulsating wave trains are a characteristic feature for the propagation within the stratospheric waveguide (Green et al. 2011). U-shapes are typical of thermospheric phases (Assink et al. 2018). With increase in distance, a dispersion is evident for tropospheric phases (Vergoz et al. 2019). The amplitude is generally higher for atmospheric than for underground explosions (Park et al. 2018). Converted infrasound waves (seismoacoustic waves) tend to have less pronounced onsets as the coupling between seismic and acoustic waves is influenced by many factors (Fuchs et al. 2019). Mining explosions and quarry blasts often show simple pulses with sudden onset (e.g. Campus and Christie 2010). Hagerty et al. (2002) describe two different types of signals, depending on the type of explosive source. Type 1 consists of one to two pulses, with the second pulse occurring more than 50 s after the first signal onset. Type 2 is an amplitude-increasing signal of up to 30 s in length and shows a series of single pulses.

Explosions generate infrasound signals within a broad frequency range (0.01 to > 4 Hz). Surface mining explosions and quarry blasts produce infrasonic signals in the range between 1 and 5 Hz and are often detectable at long distances (e.g. Stump et al. 2001; Arrowsmith et al. 2008; Czanik et al. 2021). Ceranna et al. (2009) detect infrasound waves in the range between 0.1 Hz and 2.0 Hz for the Buncefield oil depot explosion and the explosion of the Antares rocket showed a dominant frequency of 0.4 Hz (Vergoz et al. 2019). The frequency thereby depends on the size of the explosions (Norris et al. 2005), on the detonation environment as well as on the propagation path and the distance between source and receiver. Atmospheric explosions produce fewer high-frequency signals than underground (nuclear) explosions. Christie et al. (2005) and Christie and Kennet (2007) have detected signals from small atmospheric explosions in the frequency range of 0.5 to 2 Hz and 0.4 to 1.2 Hz, respectively. According to Assink et al. (2016), small-sized atmospheric nuclear tests of about 1 kt TNT equivalent are expected to generate infrasound in the frequency range of 0.1 to 0.2 Hz, whereas underground nuclear explosions are expected to generate seismoacoustic signals with higher frequencies in the range of 2–4 Hz (Schneider et al. 2018) or 1–2 Hz (Fuchs et al. 2019), depending on the source. At larger distances, the high-frequency part (> 0.5 Hz) of the signal is often lost due to attenuation effects (Christie et al. 2005; Christie and Kennet 2007). Thermospheric signals show a lower-frequency content (0.01–0.1 Hz) than tropospheric or stratospheric phases, which are characterized by frequencies > 0.1 Hz (Christie and Kennet 2007; Vergoz et al. 2019). Che et al. (2009, 2014) measure epicentral “lt” phases with a dominant frequency around 1.0 Hz and “Is” phases between 2.6 and 4.3 Hz for the North Korean nuclear tests.

**Fig. 6:**
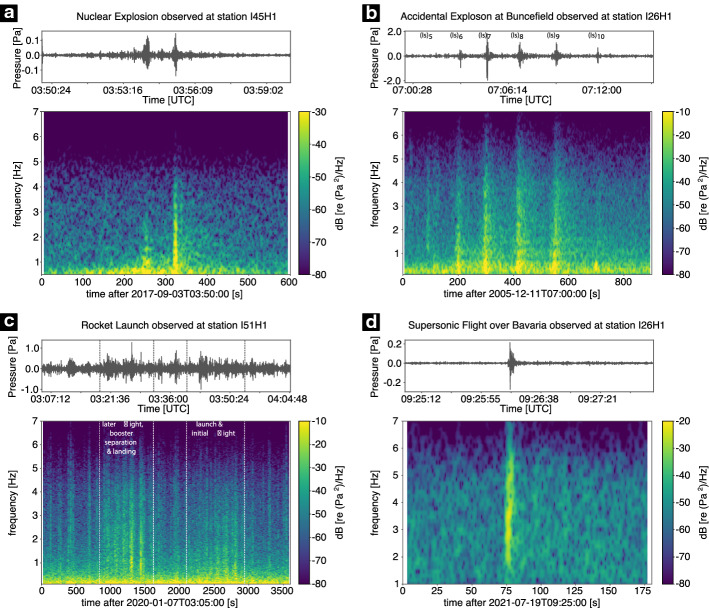
Examples of recorded infrasonic waveforms and associated spectrograms for four anthropogenic sources at stations of the IMS infrasonic network. The waveform and spectrogram of the last underground nuclear test explosion (DPRK 6) in North Korea recorded at element I45H1 of the infrasound array IS45 in eastern Russia are shown in **a**. **b** shows the waveform and spectrogram of an accidental explosion at the Buncefield Oil Depot, UK, recorded at element I26H1 of the infrasound array IS26. Different phases are marked on the waveform record. The record of the launch of a Falcon 9 rocket from Cape Canaveral recorded at element I51H1 of the infrasound array IS51 in Bermuda is displayed in **c**. Signals associated with the launch, different flight stages and booster separation are marked. The waveform and associated spectrogram of a supersonic flight over Bavaria, Germany, recorded at element I26H1 of the infrasound array IS26 is shown in **d**. The waveforms are bandpass filtered between 0.5 and 4 Hz (**a**), 0.1 and 4 Hz (**b**, **c**), or 1 and 5 Hz (**d**). The spectrograms are calculated with either 90% (**a**, **b**, **c**) or 99% (**d**) overlapping windows with a length of 2^8^ (**a**), 2^8^ (**b**), 2^10^ (**c**), or 2^7^ (**d**) samples, respectively. For more information, see Table 2 (Appendix)

As an example for a nuclear explosion, the infrasonic waveform of the most recent North Korean underground nuclear test (DPRK 6) conducted on 3 September 2017 at 03:30:01 UTC recorded at IMS array IS45 (Russia) is shown in Fig. 6a. The event also produced strong seismic waveforms, which are shown in Fig. 2a. Figure 6b shows the waveform from an accidental explosion at the Buncefield Oil Depot, UK, in December, 2005, recorded at an element of the infrasound array IS26 in Germany. Different phases can be clearly identified on the recorded waveform.

#### Rocket Launches

Scientific and military rocket launches and re-entries generate infrasound signals that have been detected since more than 50 years (e.g. Kaschak et al. 1970; Evers et al. 2018; Pilger et al. 2021a). Publications by Donn et al. (1968), Kaschak et al. (1970), and Balachandran and Donn (1971) analysed infrasound signals associated with the rocket launches of the early manned spaceflight programme (Saturn V, Apollo). Olson (2012) gives an overview of the infrasound signatures of a variety of rockets ranging from small sounding to larger spaceflight rockets, Evers et al. (2018) analyse the infrasound from four rocket launches for military purposes by the Democratic People’s Republic of Korea in 2009 and 2017, and Pilger et al. (2021a) analysed the infrasound signatures of 1001 rocket launches for space missions between 2009 and mid-2020 on infrasound stations of the IMS. Rocket launches represent well-defined ground-truth events, as usually their launch time as well as the trajectory are precisely known (e.g. McLaughlin et al. 2000; Pilger et al. 2021a).

Infrasound signals are generated by ignition, burning, and re-entry of rocket stages (e.g. Pilger et al. 2021a). The principal sources are aeroacoustic energy generated by supersonic engine exhaust and the Mach cone that forms when the rocket reaches supersonic velocities (Kaschak et al. 1970; Cotten and Donn 1971; Blom et al. 2016; McLaughlin et al. 2000). Two distinct sets of signals are registered, where one group represents the sound from the direction of the launch site (Donn et al. 1968; Balachandran and Donn 1971) and the other represents the sound originating from the re-entry of the first or second stage. If the receiver is located along the rocket’s trajectory, the re-entry signal group is observed first because of the high velocity component of the rocket approaching the receiver (Donn et al. 1968; Kaschak et al. 1970). Signal families are often observed for the different flight phases (e.g. take-off, ascent, descent, landing), arriving at the sensor along different propagation paths. Rocket signals can be distinguished from explosions due to their moving nature and much more complex source characteristics (Evers et al. 2018; Pilger et al. 2021a).

Depending on the rocket type, source mechanism, and the source-receiver distance, frequencies in the range of 0.1 Hz to 5 Hz, with the majority of energy found between 0.1 Hz and 2 Hz, are observed (e.g. Kaschak et al. 1970; McLaughlin et al. 2000). Signals of group one and two exhibit energy in the range of 0.14–0.5 Hz, and 0.1–0.33 Hz, respectively, and Kaschak et al. (1970) found a slight difference in frequencies between the re-entry (0.4–2.5 Hz; peak at 0.65 Hz) and launch phase (0.25 to 1 Hz; peak at 0.6 Hz). The frequency content of signals registered at short distances differs from those registered at longer ranges, mainly due to propagation effects (McLaughlin et al. 2000). Within a registered signal a shift to lower frequencies if the rocket ascends and accelerates away from the infrasound sensor and to higher frequencies if the rocket moves toward the sensor is observed caused by the Doppler effect (Olson 2012; Pilger et al. 2021a).

Signals of a few (3 min; Kaschak et al. 1970) to several minutes (~ 10 min) in length with gradually increasing and decreasing amplitudes (Balachandran and Donn 1971; McLaughlin et al. 2000) have been detected, with the signal length correlating with the size of the re-entering objects and with burn time in the near field (Blom et al. 2016). The signal shape changes from impulsive to more emergent with increase in distance (Evers et al. 2018). The signal strength/amplitude depends on thrust, trajectory, velocity, and sound speed as well as wind along the path (McLaughlin et al. 2000; Blom et al. 2016). The individual phases during take-off and in-flight as well as the motor type based on the waveform can be distinguished. While solid motors exhibit a strong initial transient, this is absent in liquid motors, whose waveform is characterized by a slow rise in amplitude (Olson 2012; Blom et al. 2016; Pilger et al. 2021a).

In Fig. 6c, an example waveform and the associated spectrogram of the launch of a Falcon 9 rocket at Cape Canaveral, detected on Bermuda (IS51) are displayed, showing the signals of the later flight stage first—i.e. booster separation and booster landing—followed by signals associated with the launch and initial flight stage***.***

#### Sub-/Supersonic Aircrafts, Helicopters, Atmospheric Re-entries

Objects such as supersonic aircrafts, re-entering spacecrafts, and sample return capsules generate infrasound by moving through the atmosphere at supersonic speeds. By moving at supersonic or hypersonic (defined as five times the speed of sound) speed, these objects generate a shock wave, resulting in the typical N-shape signals (e.g. de Groot-Hedlin et al. 2010) that can be registered at large distances (e.g. Liszka 1978).

The Hayabusa Spacecraft and the Hayabusa Sample Return Capsule generated distinct shock wave signals with fundamental frequencies of 7 to 8 Hz and the typical N-shape (Yamamoto et al. 2011; Ishihara et al. 2012). On transatlantic flights to Europe, infrasound waves generated by the Concorde could be detected in Northern and Central Europe (Liszka and Waldemark 1995; Pilger et al. 2018). In addition to supersonic motion, acceleration associated with a change in the flight path (trajectory) can also generate an infrasonic signal, as it also generates a shock wave (Garcés et al. 2004).

Helicopters generate infrasound by the movement of the rotor blades (e.g. Hood and Leventhall 1971; Garstang 2010). The frequencies that occur reflect the blade-pass frequency and associated harmonics and are dependent on the rotor revolution rate as well as the number of rotor blades. Finnegan et al. (2021) measure infrasound frequencies of, e.g. 28 Hz and 11.5 Hz for different helicopters and observed a Doppler shift of the signals during flight.

Figure 6d shows the recording of a recent military supersonic flight over Bavaria, Germany, recorded at IMS station IS26.

#### Controlled Infrasound Sources

Several sources such as conventional loudspeakers, pneumatic sources, and systems based on gas combustion have been developed for the controllable generation of infrasound. An overview of the different developments is given by Gorhum (2014). The first development, the so-called mobile acoustic source (MOAS), often referred to as the “mother of all speakers”, was made by the US Army and The National Center for Physical Acoustics at The University of Mississippi. This is a massive horn-coupled electro-pneumatic loudspeaker that is capable of generating signals down to 8–10 Hz (e.g. Gorhum 2014). Walker et al. (2008) present a concept based on arrays of conventional subwoofers. With the arrangement of loudspeakers in arrays, it is possible to generate signals with minimum frequencies of 8 Hz (Park et al. 2009). Gorhum et al. (2013; 2020), as well as Muir et al. (2013) and Barlett et al. (2020) use the siren concept to generate low-frequency signals in the infrasonic range. Gorhum et al. (2013) present a pneumatic source that consists of three components, namely a reservoir, a rotor/stator pair, and a motor to drive the rotating ball spindle. The source generates infrasound in the frequency range from 1 to 8 Hz by periodically releasing compressed air through the rotating ball valve. Muir et al. (2013) also use streams of compressed air released by rotating spherical valves and Barlett et al. (2020) describe a similar source, which generates narrowband signals in the frequency range between 0.25 and 10 Hz. Another efficient and compact infrasound source is the so-called rotary subwoofer or rotary speaker, that consists of a baffled fan with blades having variable pitch, driven by an electric motor (Park and Robertson 2009; Park et al. 2009). The fan is coupled to a back volume. With the rotary subwoofer it is possible to generate frequencies between 5 and 8 Hz (Smith and Gabrielson 2018). Smith and Gabrielson (2018) investigate the use of gas combustion, namely an air-propane burner, for the generation of infrasound waves. A coherent infrasonic signal with a frequency between 0.25 and 4 Hz is generated by thermal expansion and contraction due to heating of the air mass (e.g. Smith and Gabrielson 2020).

### Other Anthropogenic Sources of Infrasonic Waves

#### On-shore Wind Turbines

Alongside audible sounds, wind turbines also generate sound components in the infrasonic range (e.g. Pilger and Ceranna 2017). The infrasound signals depend on wind speed and direction, blade passing frequency, rotational speed, and total number of turbines. The propagation of infrasound occurs mainly in the atmospheric boundary layer (lower part of the troposphere), which varies in height from tens of metres to several kilometres (Marcillo et al. 2015). Propagation is best under calm wind conditions, whereas it is affected by increasing wind speed and turbulence (Styles et al. 2011).

There are different mechanisms that generate sound in wind turbines. Infrasound is mainly aerodynamic noise related to the interaction of the blades, the supporting tower, and flow gradients as well as turbulences (e.g. Tonin 2012; Marcillo et al. 2015). An important factor in this context are blade-tower interactions, which generate the main part of the observed infrasonic signals (e.g. Pilger and Ceranna 2017). Distinct discrete frequencies and a number of associated harmonics are generated when a rotor blade passes the tower. An interaction occurs between the rotors and the wind speed deficit due to the blocking effect of the tower. The tower thus causes a disturbed airflow, which exposes the rotor blade to a fluctuation in that airflow (i.e. a load deficit) at each revolution, which results in a change in uplift. Consequently, impulsive sound signals are generated (e.g. Jakobsen 2005; Marcillo et al. 2015; Pilger and Ceranna 2017; Hansen and Hansen 2020). In addition, interactions between the rotor blades and turbulence in the airflow generate broadband sound due to the change in blade loading caused by changing conditions with turbulence (Marcillo et al. 2015; Hansen and Hansen 2020).

The observed fundamental frequencies are in the range between 0.9 Hz (Marcillo et al. 2015) and 1.4 Hz (Pilger and Ceranna 2017). Multiples of these frequencies, the harmonics, are also observed (e.g. 2.7 Hz, 4.1 Hz, 5.4 Hz, 6.9 Hz; Pilger and Ceranna 2017). These fundamental frequencies reflect the time it takes successive blades to approach and pass the rotor tower (e.g. Hansen and Hansen 2020); thus, they correspond to blade passing harmonics (BPH) and are variable as a function of rotor and wind speed (e.g. Keith et al. 2018). The fundamental frequency increases with increase in wind speed and larger turbines produce lower frequencies (Hansen and Hansen 2020).

The signals have a pulse-like character. The polarity depends on the direction in which the measurement takes place in relation to the wind turbine, i.e. in which direction the movement of the blades is directed. If the movement is directed away from the sensors, the first peak is expected to be in the negative direction (Pilger and Ceranna 2017). The amplitude of the signal decreases with increase in wind speed (Styles et al. 2011) and is also dependent on any turbulence that occurs, thus it can fluctuate from revolution to revolution. Overall, the impulse-like signals repeat with a frequency depending on the number of rotor blades and their rotational speed (Pilger and Ceranna 2017). The frequency peaks become broader with increase in sensor distance from the wind turbine as well as for higher harmonics.

#### Musical Instruments

In the musical sense, the frequency range of infrasound covers four octaves between 1 and 16 Hz, but only the top one has a musical designation. The range between about 8 and 16 Hz is called double contra octave. The next higher octave (sub-contra-octave; 16–19.5 Hz) also reaches into the infrasonic range (Mühlhans 2017). There are only two instruments in the world that can produce true infrasonic sounds. These are the Sydney Town Hall Organ in Sydney, Australia, and the Convention Hall Organ in Atlantic City, USA (Fletcher 2000; Mühlhans 2017). Organs produce sound through air vibration. A stream of air is directed over an opening and against a sharp lip, the labium, causing the air stream to flutter, which creates pressure waves in the air column of the pipe (Gupfinger 2009). The Sydney Town Hall organ is able to produce a fundamental frequency of 8 Hz as its lowest note because it has a full-length 64-foot pedal reed stop (contra-trombone; Fletcher 2000). The pipe of the Atlantic City Convention Hall organ also has an open 64-foot stop with a pipe length of 19.7 m and can likewise generate a tone with a fundamental frequency of 8 Hz (Mühlhans 2017).

### Ambient Noise

Noise refers to all signals that are not generated by the sources of interest (Bowman et al. 2005a). The ambient noise field contains both coherent and non-coherent components and sources (Matoza et al. 2013) and is highly variable in terms of station, time of day and season (e.g. Le Pichon et al. 2009). The noise field in general is created by a superposition of pressure fluctuations from various local, regional and global sources such as microbaroms, wind, traffic, or industry (Christie and Kennet 2007; Marty et al. 2017). Ambient noise covers the entire frequency range between 0.03 and 10 Hz (Bowman et al. 2005a), with individual sources found in certain narrower frequency bands. The main sources are microbaroms (long-range pressure fluctuations generated over the ocean with a frequency between 0.1 and 0.6 Hz) and wind noise (short-range pressure fluctuation) due to local eddies and winds (Bowman et al. 2005a; De Carlo et al. 2021).

Wind is generally considered the most important source of noise, generating pressure fluctuations by hydrostatic and dynamic effects (wind turbulence) in the atmospheric boundary layer (Christie and Kennet 2007; Le Pichon et al. 2009; Marty et al. 2017) and is responsible for the majority of pressure fluctuations in the frequency range between 0.01 and 5 Hz (Matoza et al. 2013). Wind also generates measurable noise in the range between 0.003 and 0.01 Hz (Alcoverro and Le Pichon 2005). However, these turbulent air movements near the ground are very complex. The amplitude of wind-generated noise correlates with wind speed (Alcoverro and Le Pichon 2004) and varies with geographical location and time of day and year (Matoza et al. 2013). Another source contributing to the ambient noise field is surf (e.g. Le Pichon et al. 2004) with frequencies around and above 1 Hz (e.g. Christie and Kennet 2007; Pilger et al. 2019). In addition, aurora, mountain-associated waves (f < 0.1 Hz), and cultural sources such as traffic, trains, aircraft, and industry (1–10 Hz) also contribute to the noise spectrum. Recurrent or constant sources are waterfalls, rivers, thunder, or ongoing volcanic eruptions (Christie and Kennet 2007).

## Discussion

In the preceding sections, we have described a variety of seismic, hydroacoustic, and infrasonic sources with respect to their spectral properties as well as signal characteristics and underlying generation mechanisms. Now it is necessary to rate/classify the individual sources and their signals with respect to their use for sensor calibration and in comparison with previous studies. First of all, we will address the aim of the calibration, its implementation and its benefits, before evaluating the sources with regard to the most important factors. As stated in Sect. 2, the most important factors to consider are the repeatability of the signals, the frequency bandwidth, the determinability of or the knowledge about the spatial and temporal location as well as the size/strength of the source. Another important point is the practicability in the application and in terms of cost–benefit of the source for calibration purposes. Using an evaluation scheme, we have evaluated and ranked the sources for each of the three waveform technologies (Table 1).

**Table 1:** Classification of the SHI sources with respect to the parameters important for their usage as calibration signals. The plus and minus signs indicate how well the respective source covers the parameter and “o” denotes neutral

Source		Parameter						Limitations	comments
		Repeatability of signal^1^	Frequency range^2^	Known Location & Time^3^	Known size/Yield^3^	Practicality^4^	Calibration		
Seismology	Earthquakes	+	++	+	+	+	+		Frequency range depends on type
	Volcanoes	o	o	+	o	–	o\−	Frequencies > 10 Hz missing	Very low frequencies can only be observed in the near-field
	Microseisms	++	–	+	o	+	+		Lower-frequency range
	Earth hum, tides	++	–	+\o	o	o	o\–	Only observed after strong EQs or at very quiet sites	Only lowest frequency range
	Rockfall/Avalanches/etc	o	+	o	o	o	o	Frequencies < 10 Hz are often missing Local, small radius	
	Thunderstorms	+	o	o	–	o	o	Limited frequency range, lower & upper part are missing Very local	
	Meteoroids	–	o	o	o	o	o	Rare	
	Cryosphere	o	+	+	–	o	o		
	Natural noise	+	+	–	–	+	o		Frequency depending on source
	Explosions/Mining activity	+	+	++	+	+	+		
	Cultural noise	+	+	–	–	+	o		Frequency depending on source
	Induced seismicity	+	+	+	+	o	+		
	Drop weights, hammer	++	o	++	++	++	++		
	Vibration sources	++	o	++	++	+	+		
*Hydroacoustic*	Earthquakes	+	+	+	+	+	+		
	Tsunami	–	o	o	o	–	–	Frequencies > 0.01 Hz are missing	
	Submarine landslides	–	+	o	o	–	o\–	Do not occur very often	
	Submarine volcanoes	o	+	o	o	o	o	Localization	
	Bioacoustic sources	+	+	–	–	–	o\–	Localization	Frequency range depending on species
	Icebergs	o	+	o	–	–	o\–		
	Natural noise	o	+	–	–	o	o		Frequency range depending on source
	Explosions	o	+	+	+	+	+		
	Active sources	++	+	++	++	++	++	Expensive	
	Ship traffic	+	o	+	o	o	o		
	Drilling, dredging	o	–	++	o	o	o		
	Wind turbines	++\+	−−	++	+\o	o	+\o	Shallow water	Studies rather focused on the upper frequency spectrum
	Loudspeaker	++	o	++	++	+	+		
*Infrasound*	Meteors	o	o	o	+\o	o\–	o	Rare	Covers lowest frequency range
	Volcanoes	+\o	+\o	++\+	o	o	+\o	Various processes	
	Microbaroms	++\+	–	+\o	o	o	+\o	Source information depending on models (with uncertainties)	Very narrow frequency band
	Storms, lightning	+	o\–	++\+	o	o\–	o\–	Very local, small radius	
	Tornado	o\–	o\–	+	o	–	o\–	Very local, small radius	Not predictable in location/time
	Aurora	+\o	–	o	o\–	–	o\–	Limited to high latitudes	Covers lowest frequency range
	Earthquakes	+\o	+\o	++\+	+	+	+\o		
	MAW	+	–	o	–	–	o	Near mountainous regions	
	Mass movements	o	+\o	+	o	o\–	o\–	Local, small radius,	
	Surf	+	+\o	o	o\–	o	o	Limited to stations close to shore; source parameters depend on models (uncertainties)	
	Cryosphere	o	o	o	o\–	o\–	o\–		
	Animals	o	o\–	–	–	–	–\−−		
	Explosions	+	+	++\+	++\+	+\o	+		
	Rocket launches	+	+\o	++	+	+	+		
	Wind turbines	++	o\–	++	+\o	+	+	Distinct frequency bands	source of noise to be avoided at (IMS) sensors
	Aircraft, helicopters	+	o\–	o	o	o	o		
	Re-entries	o	o	o	o	–	o\–	Rare	e.g. space debris
	Mining activities	+	+\o	++\+	+\o	+	+		
	Controlled sources	++	+	++	+	++	++\+		
	Musical instruments	++	–	++	o	o	o	Large, immobile	
	Noise	o	o	–	–	–	o\–		

1++: very good; +: good; o: moderate; -: uncommon; −−: poor

2 ++: 80–100%; +: 60–80%; o: 40–60%; -: 20–40%; −−: 0–20%

3 ++: very well known/determinable; +: well known/determinable; o: partly known-determinable; -: poorly known/determinable; −−: Unknown/not determinable

4 ++: very good; +: good; o: moderate; -: elaborate; −−: poor

### The Calibration and Its Intended Benefits

The aim of this study is to investigate possible excitation sources for a traceable on-site calibration of seismometers, hydrophones and microbarometers. This will be achieved through comparison measurements with a reference sensor, assuming that the reference sensor and the sensor to be calibrated measure the same coherent signal. The choice of a suitable reference sensor, previously traceably calibrated in the laboratory, the so-called transfer standard, results in the desired extension of the traceability of the calibration to the field. Although a primary calibration in the laboratory is the most accurate, there are some disadvantages. A laboratory calibration means a high time expenditure, and at large arrays such as the stations of the IMS, at least one sensor would always be missing. However, this has the consequence that the station, and thus also the network, is no longer complete and consequently the detection threshold for events is reduced (e.g. Gaebler and Ceranna 2021), the operation is disrupted and technical requirements for the operation of IMS stations are violated. Due to the remote locations of several individual stations, maintenance visits are only occasionally possible, thereby making repeated laboratory calibrations not feasible. The use of on-site calibration methods circumvents these problems.

Traceable calibrated sensors offer many benefits: the improvement of data quality and the associated enhanced confidence, increased trustworthiness and credibility of the information contained in the data, lead to enhanced international acceptance of the resulting data. Hence, the operation of global and local networks for environmental monitoring and research is supported, strengthened in its reliability and reputation. An improved data quality could also contribute to enhanced signal detection through a decrease in the detection threshold of events and a better characterization of seismicity. The indirect consequences resulting from the improved data quality are also manifold and include possibilities for environmental impact assessments such as the assessment of environmental noise pollution from industry, resource exploration, or shipping. In addition, other environmental and social aspects ranging from investigations of changes in the ocean temperature and polar ice coverage, climate change, extreme events such as earthquakes and volcanic eruptions to monitoring of illegitimate nuclear testing can be investigated more reliably. The estimation of measurement uncertainties and their propagation throughout the calibration process will also provide improved opportunities for understanding sensor performance in relation to temperature or humidity variations and extremes. Furthermore, the quantities determined from the data, such as magnitude, may be specified with reliable uncertainties.

The improved and traceable calibration further enables the use of the data by various policy makers and international agreements, such as an assessment of noise pollution of the oceans. Examples include the Oslo-Paris Agreement (OSPAR) and the Marine Strategy Framework Directive. The OSPAR commission shows the impact of different anthropogenic sounds in comparison with natural sounds in the ocean in a summative figure (OSPAR 2022). The recording of various hydroacoustic, especially anthropogenic sources in the low-frequency range, could contribute to an expansion of the data basis of this figure and thus also to new guidelines for the protection of marine life. A similar influence exists in the area of on-shore and off-shore wind farms. A better assessment of the low-frequency noise generated and thus the environmental impact on both humans and other living organisms would enable more informed decision-making and thus promote the expansion of renewable energies. Further, summative figures connecting frequency and wavelength of different signal sources, such as the one provide by Silber (2014), will be improved.

A calibration of the sensors thus provides traceable data that has a large impact on scientific, environmental, economic, social as well as political communities.

### Frequency Content

**Fig. 7:**
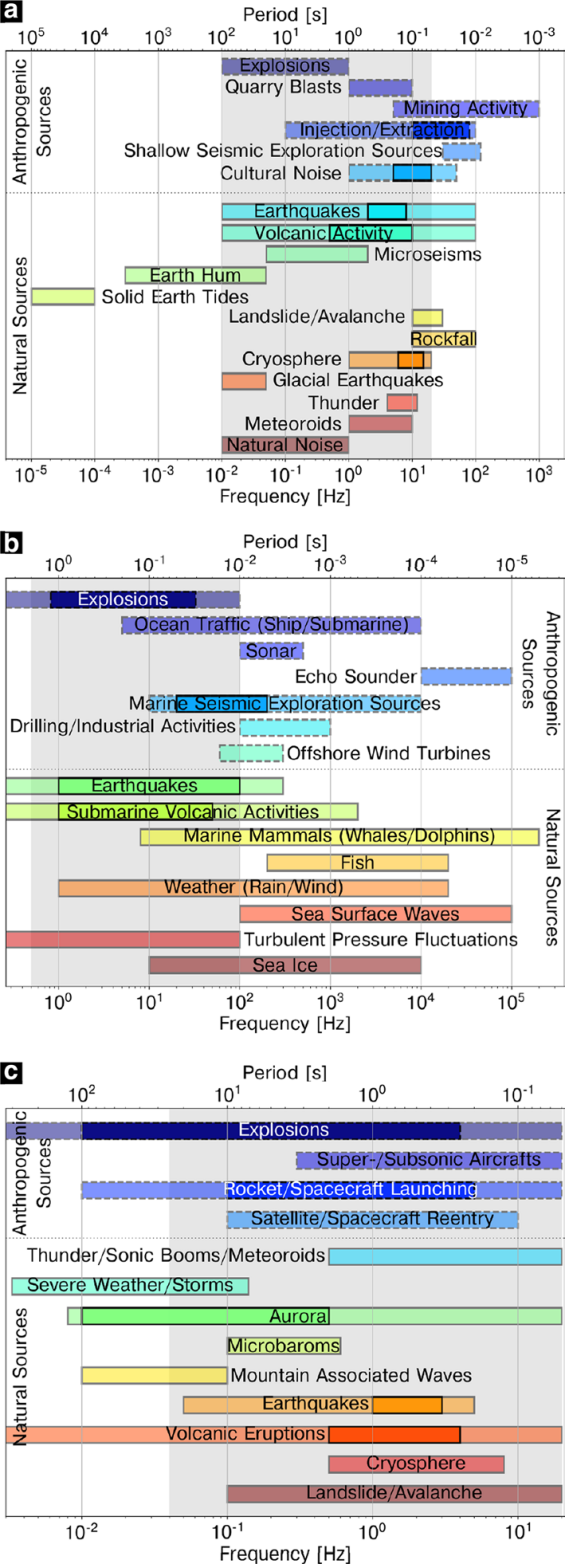
Observed frequency ranges for seismic (**a**), hydroacoustic (**b**), and infrasonic (**c**) waves. Dashed-bordered boxes illustrate anthropogenic sources, solid-bordered boxes illustrate natural sources of each wave type. More saturated colours indicate commonly observed and dominant frequency ranges. The frequency ranges to be calibrated (0.01–20 Hz for seismic waves; 0.5–100 Hz for hydroacoustic waves; 0.04–20 Hz for infrasonic waves) are highlighted in grey. Note that only the most important and not all sources are included in the figure, some sources such as tsunamis, which are well outside the frequency range under consideration, have not been included for reasons of clarity. The values are taken from the review and are complemented by values from Dahlman et al. (2011) and Carroll et al. (2017) for hydroacoustic waves, and by values found on the NOAA infrasound website (https://psl.noaa.gov/programs/infrasound/infrasonic.html) and in Le Pichon et al. (2010) for infrasonic waves

If we look at the evaluated sources of all three waveform techniques, most of them generate signals in the desired frequency range (Fig. 7) between at least 0.01 and 20 Hz (0.01–20 Hz for seismic waves; 0.5–100 Hz for hydroacoustic waves; 0.04–20 Hz for infrasonic waves). However, sources often only cover a part of this range with a rather small bandwidth. For classification purposes, we have calculated the percentage of the signal frequencies within the desired frequency range. Since the frequency range to be considered covers at least two decades, the logarithmic values of the frequencies were used. The frequency evaluation also takes into account that often several signals are combined in one category (e.g. earthquakes, volcanoes, or explosions/mining activity), which together cover a large frequency band, but individually may cover a much smaller range.

As a natural source of seismic waves, earthquakes cover the entire frequency range (> 80%); however, this depends on the earthquake type (e.g. Tectonic Earthquakes, Tectonic Tremor, Slow Earthquakes) and other parameters such as the epicentral distance, thus not the entire spectrum is covered in every case. Mass movements, as well as natural noise sources and the ice masses, cover a large part of the required frequency range (60–80%), with the first source generating mainly frequencies above 10 Hz. Signals emitted by volcanoes, meteoroids, and thunderstorms each contain about 50% of the frequencies under consideration. Microseismicity and Solid Earth tides cover only a small portion (20–40%) of the frequency spectrum. However, as the generated frequencies are found in the lowest range, they have a special significance.

Almost all examined natural sources of hydroacoustic waves cover a large part (> 60%) of the frequency range between 0.1 and 20 Hz. Only tsunami signals, which are found at the lower end, show a minor agreement. The evaluated natural sources of infrasonic waves produce signals in the significant range, but none of them covers more than 70% of it. Signals associated with various volcanic processes, earthquakes, surf, and mass movements each cover about 50 to 70% of the range of interest. Signals originating from meteoroids and ice masses have a slightly lower correspondence (40–60%), whereas, microbaroms, weather phenomena, or animals produce signal frequencies within a small range only (< 40%). Although auroras, volcanic activity, and meteoroids cover only portions of the desired frequency spectrum, it should be emphasized that these comprise the lowest frequencies of particular interest. Microbaroms also are outstanding because of the narrow, well-defined frequency band.

On the anthropogenic side, explosion sources and sources related to mining activities (e.g. quarry blasts) generate seismic signals that contain a large part (60–80%) of the appropriate frequencies. Different sources contributing to cultural noise also show suitable frequency content. Controlled sources such as drop weights, sledge hammers, or vibration sources each cover about 50% of the frequency range to be calibrated. Triggered and induced seismicity hold a special, rather hybrid position here, since both the signal characteristics and the generation mechanisms are very similar to earthquakes. Anthropogenic sources of hydroacoustic waves generate predominantly higher frequencies and thus often lie above the range of interest. Explosions and controlled sources like airguns should be pointed out, which partly fall into the relevant frequency range and cover the highest proportion of it. Among the signals generated by anthropogenic infrasonic sources, explosions, mining activities, rocket launches, and controlled sources each cover a large portion of the frequency range of interest for calibration (about 50–80%). Wind turbines generate only certain narrow frequency bands and associated harmonics, so they cover a very limited frequency range. Signals from infrasonic noise sources, re-entries, and aircraft have a frequency content agreement of about 30 to 50%.

### Signal Repeatability

Repeatability of the signal primarily aims at the stability of the waveform or, in other words, means that the waveforms are comparable, reproducible in their characteristics (magnitude/size, frequency bandwidth) and have a high degree of similarity when a signal of the same source reoccurs. This is important to ensure comparability between calibrations and to detect any drift of the sensor. In order to classify the signals, we rated the signal repeatability from very good (++) to poor (−−; Table 1).

Natural sources of seismic waves display a large variety of individual signals, but stable signal shapes with little variability for common sources processes are observed, especially in microseismicity and earth tides. Comparable waveforms often occur in earthquakes, thunderstorms, and in the signals from natural noise sources. For example, there are so-called “repeating earthquakes” (e.g. Geller and Mueller 1980; Uchida 2019; Uchida and Bürgmann 2019) that occur with similar mechanisms at roughly the same location thereby producing signals with comparable characteristics. The waveforms also have a very high degree of similarity as a result of the constant travel paths and the fact that the medium changes only slightly, which means they can be used as a kind of “ground truth”. Looking at the spectra of these signals, differences are therefore mainly sensor-related. The signal stability is less reliable for signals related to volcanic and cryospheric processes, which include a large diversity of source processes. Likewise, the signal shapes of mass movements are only rarely comparable with each other, but nevertheless exhibit common spectral features. Similar to sources of seismic waves, natural reoccurring sources of hydroacoustic waves generally produce signal shapes that differ from one another. Similarities in signal shape exist for earthquakes. Bio-acoustic sources are outstanding, being characterized by good signal stability/repeatability. The signals of one species are comparable with each other and display distinct features, but each individual also shows peculiarities in the signal. Looking at sources of infrasonic waves microbaroms, MAWs and surf are characterized by stable, low variability waveforms. The signal shapes of earthquakes, volcanoes, and aurora each exhibit a good similarity between different events. In comparison, the signals generated by, e.g. animals, the ice masses, mass movements, or tornadoes are characterized by a high variability. For both hydroacoustic and infrasonic sources, it is important to note that the propagation medium has a greater influence on the signal shape than for seismic sources because the medium changes on significantly shorter time scales.

Anthropogenic seismic signals are usually characterized by good to very good signal repeatability. Especially the signals of controlled seismic sources such as drop weights or vibration sources show a stability of nearly one hundred percent. Almost exactly repeating signals can be generated with controlled hydroacoustic sources like airguns or underwater loudspeakers and controlled explosion sources such as SUS show high signal similarity. As with the other two waveform techniques, anthropogenic sources in the infrasound field show a much better signal repeatability between events compared to natural sources. In particular, the waveforms from wind turbines and controlled sources vary little, whereas the waveforms from re-entries and mining activities are significantly more variable.

In particular, the waveform is also affected by the path through the propagation medium. Thereby, the path length has an influence on the frequencies present in the registered signal. As the propagation path increases, the proportion of high frequencies usually decreases due to attenuation effects. In addition, any reflections, phase shifts and scattering can change the waveform along the propagation path from the source to the sensor. The influence of the propagation medium plays a role especially in a time-varying media such as the atmosphere or the ocean. Signals from controlled sources are usually not affected by this, since they have only short propagation paths; they are therefore characterized by a stable waveform. Local effects like wind turbulences can also contribute to varying signal shapes. It should also be noted here that signals can change measurably even within short distances of a few meters. If the signals from the same source are observed at several sensors located close to each other, the coherence between the measured signals must be considered for the use as calibration signal. Only if the signals have a high coherence, they can be considered for calibration.

### Knowledge About Event Origin Time, Location and Size

For calibration purposes, knowledge of source parameters such as the amplitude and size as well as origin time and location of the excitation signal is of importance in order to relate and compare the output with the physical quantities and measurement uncertainties. Since signals are needed that exceed the noise level at the station, the size (magnitude, yield, etc.) should be known or determinable. The determination of the size is often done by applying empirical relations. Furthermore, knowledge of the origin time and location of the events is necessary and must be well determinable. These parameters help to identify suitable time periods with eligible signals in the recorded data. After calibration of the sensors, these parameters can be determined more precisely and also assigned with measurement uncertainties, which increases their confidence and reliability. Especially for controlled sources these parameters are of interest, because they can be varied easily and thus the influence of these factors on the calibration result can be investigated. Based on general experience and the source description above, we categorized this parameter between very good (+ +) and poor (−−; Table 1).

While the location and time of many natural sources of seismic waves are known or can otherwise be identified very well (e.g. earthquakes, volcanoes, microseisms, or noise), the magnitude is determined by empirical equations and therefore is often subject to uncertainties. Concerning natural sources of hydroacoustic waves, the location as well as the determination of the magnitude is quite moderate. Especially earthquakes can be localized well in space and time, while the location of, e.g. submarine volcanoes and bio-acoustic sources is rather difficult. For natural infrasonic sources, the time and location are not always or not exactly determinable; however, these parameters have a good to very good determinability for volcanos, earthquakes, or thunderstorms. Estimates about the size or magnitude of the event are rather good for meteoroids and earthquakes, whereas for most infrasonic sources the determinability is rather moderate.

Both the location and magnitude of anthropogenic sources of SHI waves are often well known. An exception are sources that contribute to cultural seismic noise and moving sources such as re-entries and aircraft or helicopters for which it can be harder to determine the location and time. Identifying the location and time of noise sources is rarely possible with sufficient accuracy. Although often subject to large ranges of uncertainty, the yield determination of a source works relatively well for explosions using empirical relations. In some cases, the yield is even well known, e.g. for controlled explosions. Similarly, the source signal strength can be determined well for, e.g. controlled sources or rocket launches. For noise, this again is difficult and not precisely possible.

### Practicability and Applicability for Calibration

The practicability and applicability include the recording time required, the repetition rate of events or, in the case of controlled sources, the costs and effort in field applications. For instance, a particular source may generate a wave that is eminently suitable as an excitation signal for calibration in terms of its inherent properties, but it proves to be impractical as it occurs only infrequently. Again, we rated the sources in terms of their practicability and applicability for calibration purposes between very good (++) and poor (−−; Table 1).

Seismic, hydroacoustic, and infrasonic signals from earthquakes seem to be well suited as natural excitation sources for calibration as they occur frequently, that is about 150 earthquakes per year with a magnitude of 6 and greater (USGS 2022), everywhere on Earth and are observed at great distances from the source. The amplitude is generally much larger than the sensors’ self-noise and the waveforms are stable/repeatable to some extent, allowing for repetitive and comparative measurements. Other signals tend to be less practical. Mass movements and weather phenomena occur locally and are usually detectable only within a certain radius to the source. The detection of surf is limited to sensors within a short distance from the coast, and aurora is only observed at high latitudes, limiting their applicability to certain geographic areas. This also applies to signals related to volcanic activity, which additionally appear less practicable because of the great number of involved processes. Although both terrestrial and marine volcanic activity is very widespread and signals from different volcanoes exhibit common characteristics, the repetition rates for individual volcanoes are quite variable and can sometimes be very long, making comparisons difficult. Moving sources (e.g. meteoroids, marine mammals) and submarine volcanic activity often lack exact localization. Some signals (e.g. MAW, Earth tides, microseisms) cover only very small portions of the desired frequency range. Often, however, these are of special significance as the cover the lowest frequency range of interest and the excitation of such low frequencies with, e.g. controlled sources still is difficult and associated with high costs. Nevertheless, these natural signals often require long time series to be recorded either to cover the desired frequency range or because the signals occur only occasionally. While Earth tides are always observable, data must be registered for a long period of time for detecting these low-frequency signals. Natural oscillations of the Earth (seismic waves) as well as tsunami signals (hydroacoustic waves) are linked to (very) large earthquakes, making them rare. Meteoroids produce distinctive seismic and infrasonic waveforms that are easy to identify; however, these occur too infrequently and not likely at the same location, consequently a re-measurement with a similar signal is unlikely.

On the anthropogenic side, many sources are characterized by a good applicability. Explosions and especially controlled sources appear to be good and practical excitation sources for calibration due to their excellent properties. The later stand out mainly because of their convenience and overall great practicability. Nevertheless, there are some restrictions that need to be taken into account. Not all anthropogenic sources can be registered or applied at all stations, as the stations are often installed in very remote regions. This also leads to the fact that anthropogenic noise sources are only registered with very little energy, as these signals are not regarded as useful signals. In that case, controlled sources can be applied, e.g. during maintenance work at the stations several times a year. Limiting factors with hydroacoustic controlled sources are the associated costs, which are comparatively high since the calibration of hydrophones with controlled sources requires the use of vessels together with the application of airguns. Controlled seismic sources are usually straightforward to apply and affordable. However, for some methods the costs as well as the size of sources could be limiting factors. For large vibratory vehicles, some level of infrastructure is needed in the vicinity of the sensors. Additionally, for calibration experiments with controlled sources only frequency information are obtained, but no phase information. If the phase response of the sensor is to be determined during calibration in addition to the frequency response, the type of signal probably plays a role. In general, impulsive signals, e.g. signals generated by hammer blows or drop weights, are minimum-phase signals, i.e. the energy is maximally front loaded and the rate of energy build-up in time is fastest. For sweep signals, one gets a maximum-phase signal, i.e. the bulk of the energy is at the signal end and the rate of energy build-up is slowest. A minimum-phase signal has only very small phase changes, while a maximum-phase signal shows the largest phase changes. With controlled sources, dispersive surface waves are commonly excited. In shallow-seismic field data sets where signals are recorded at different offsets from the controlled source, surface waves contribute to the wavefield and can be used to extract information about the subsurface using spectral information. An example is the so-called spectral analysis of surface waves (SASW; Stokoe and Nazarian 1983), where the phase differences between two receivers are evaluated. Therefore, an approach for obtaining the phase response of the sensors could be to apply the same source at varying distances from the sensor.

The development of controlled sources for infrasound has made progress in recent years, but requires larger equipment and a certain amount of effort. As with natural sources, the same limiting factors regarding practicality occur with some anthropogenic signal sources. Re-entries occur only rarely and wind turbines, for example, cover only a small frequency range. Offshore wind turbines are located in shallow water near the coast and the hydroacoustic signals they generate have, to our knowledge, not been observed or investigated in the deeper oceans or at greater distances from the source. Onshore wind turbines are considered to be an infrasonic noise source that should normally be avoided, and such wind turbines are thus not found in close proximity to infrasonic sensors.

### Natural and Anthropogenic Signals Applied for Calibration in Previous Studies

In situ calibration techniques have so far used a few, mainly natural sources to determine the response function of sensors (e.g. Gabrielson 2011; Wielandt 2012). Pavlis and Vernon (1994) have used ambient noise for the calibration of seismometers. Davis et al. (2005) use free oscillations of the Earth to assess the instrument responses of some stations. Davis and Berger (2007) use the Earth tides to systematically cross-check the instrument responses of the entire GSN as determined in the laboratory, for example. This is possible because the tides are observed with the strongest amplitudes at many stations worldwide, although they generally generate frequencies outside the passband of the sensors. Ringler et al. (2017) and Anthony et al. (2018) use the secondary microseismic peak to calibrate seismometers and determine absolute sensitivity estimates.

In the field of hydroacoustics, there have been very few studies addressing the in situ calibration of hydrophones (e.g. Garcia-Benadí et al. 2015; Hayman et al. 2013). Garcia-Benadí et al. (2015) use a sound pressure generator to calibrate hydrophones. In the Ascension Island experiment, airguns were used to calibrate hydrophones in situ and determine the frequency response of the system (Harben and Rodgers 2000). Most studies are concerned with calibrating hydrophones in the ultrasonic frequency range and are therefore of less interest in the present study.

Gabrielson (2011) use ambient noise to calibrate an infrasound array element. As with the use of seismic ambient noise, the duration of the record depends on the frequency being calibrated; the lower the frequency, the longer the record length. Since the signals must be coherent between the reference sensor and the sensor being calibrated, the identification of sufficiently long periods due to wind effects can be difficult. One way to identify suitable periods is described in Green et al. (2021). Marty et al. (2017) use a controlled source (infrasound generator) in addition to background pressure fluctuations for calibration. The microbarom peak is also suitable for calibration of infrasound sensors, according to Bowman and Lees (2017). In general, it is difficult to generate coherent infrasound signals with controlled sources, but there have recently been several noteworthy studies and achievements in this field (e.g. Gorhum et al. 2013; Smith and Gabrielson 2018).

The usage of ambient noise, microseisms, Earth tides, or free-oscillations is in agreement with our inferences; however, it is limited by either the required long recording time span or the rarity of the event’s occurrence and thus tends to be rather impracticable as excitation signal for calibration. The preferred utilization of controlled hydroacoustic sources is as well in accordance with our interpretation. The lack of studies and on-site applications regarding this field once again shows the necessity of considering further, well applicable calibration signals. The applications in the infrasound technology clearly show the importance of the coherence of the signals between the reference sensor and the sensor to be calibrated, and agree with our findings concerning the sources used.

According to our assessment, earthquakes appear to be a good natural excitation signal in all three technologies, but this has not been exploited in any study of which we are aware.

## Conclusions

In the low-frequency range down to 0.01 Hz, there is a lack of measurement standards and calibration procedures for seismometers, hydrophones, and infrasound sensors. As a result, data quality and traceability are significantly affected. The identification of suitable calibration procedures and excitation sources will lead to new measurement standards and therefore will contribute to an improvement of data quality and enable traceability as well as inter-station comparability. The development of laboratory-based primary and secondary calibration methods for the low-frequency range opens up the possibility of on-site calibration during operation with calibrated reference sensors. This is of particular significance, as the stations are required to be operational nearly 100% of the time regarding the safety–critical design goal of the CTBTO international monitoring system.

Following a detailed review, we rated and classified the seismic, hydroacoustic, and infrasonic sources of natural and anthropogenic origin with respect to their applicability as excitation signal for on-site calibration. We found the following necessary requirements that sources should meet in order to be considered as suitable excitation signal for on-site calibration in the low-frequency range. The frequency content is between 0.01 and 20 Hz; the signals are above the noise level of the sensors and coherent between the reference sensor and sensor to be calibrated; good knowledge about origin time, location, and size is given; and the signals are stable in their characteristics.

Many of the reviewed sources do not fully meet the deduced criteria, especially if the practicability of their application is considered. While some natural sources lack waveform repeatability and/or precise knowledge about origin time, location, and size, some anthropogenic sources are often restricted to local applications, are rare or, in the case of controlled sources, expensive to apply. However, our analysis has shown that a large number of sources may potentially be used as excitation signals. Earthquakes stand out across all three waveform technologies due to their most suitable properties. Additionally, microseisms and microbaroms can be used, as they allow a calibration at the lowermost end of the considered frequency range and have proven to be applicable in previous studies. Beyond these natural phenomena, the most anthropogenic signal sources considered meet the necessary criteria. Especially the controlled sources of each waveform technique seem to be most suitable for in-situ calibration of seismometers, hydrophones, and infrasound sensors as they show the best overall agreement with the required properties needed for excitation signals.

Due to the large number of usable sources and their specific properties, calibration is possible over a wide frequency range. Especially the low-frequency range is covered by certain sources. The use of different natural and anthropogenic signal sources for on-site calibration allows calibration during operation, which is especially important for IMS stations, and repeated calibrations with the same source signal enable comparability between stations as well as the detection of possible changes like temporal drift of the sensor. This particularly holds for permanently occurring sources like natural or cultural noise, microseisms, or microbaroms. But also controlled sources can be used for recalibrations due to their excellent signal stability. Concerning theirs use, the question of their applicability arises. Marine sources are often associated with high costs, seismic sources seem to produce signals mainly in the higher frequency range due to their application for exploration, and controlled infrasound sources have so far been mainly experimental in nature. Therefore, future tests of these sources are essential. At present, we suggest the usage of earthquake signals as natural excitation signals applicable for all three waveform technologies. Further, microseisms can be applied for seismometer and hydrophone and microbaroms for infrasound sensor calibration. As anthropogenic excitation signals we suggest to test the field application of several controlled sources as they appear to be most suitable for calibration. Following this review, we will investigate and further analyse in practice the most suitable sources as well as a variety of the other considered sources for their application in on-site calibration with a reference sensor.

## Data Availability

Access to the IMS network’s data of the seismological, hydroacoustic, and infrasound stations is available to National Data Centres of the CTBTO and can be made available to others on request through the virtual Data Exploitation Center (vDEC) at https://www.ctbto.org/specials/vdec.

## Appendix: Information About the Examples

See Table 2.

**Table 2:** Overview of the source examples in the text providing the relevant details about the shown sections and information about the events, sorted by figure number

	Date [YYYY-MM-DD]	Time UTC [hh:mm:ss]	Duration [s]	Event Location		Event origin Time UTC [hh:mm:ss]	Filter Parameters		Spectrogram Parameters		Station	Fig No	Brief description
				Latitude [°]	Longitude [°]		f_min_ [Hz]	f_max_ [Hz]	Overlap [%]	Window [samples]			
*Seismology*	2021–05-07	23:35:00	1800	18.8 S	177.4 W	23:35	1	20	90	2^10^	GEC2	1a	Teleseismic Earthquake (Fiji Region, Mb = 5.7, depth: 384 km)
	2019–07-03	12:30:00	18,000	38.79 N	15.21 E	~ 14:46 (first explosion)	1	20	90	2^12^	GEC2	1b	Volcanic Eruption (Stromboli volcano, Italy)
	2020–02-16	20:00:00	57,600	-	-	-	0.04	1	90	2^14^	GEC2	1c	Microseisms (from Atlantic Ocean)
	2011–12-27	17:25:00	600	46.2950 N	9.6020 E	17:25:43	1	20	90	2^9^	GEC2	1d	Rockfall at Piz Cengalo (Switzerland)
	2017–09-03	03:30:00	1800	41.3 N	129.08 E	03:30:01	1	20	90	2^10^	GEC2	2a	Underground Nuclear Explosion (DPRK6—North Korea; yield 160–400 kt
	2011–11-18	14:49:00	180	~ 49.0026 N	~ 11.4258 E	14:49:40	1	20	90	2^9^	GEC2	2b	Quarry Blast (Stein- & Schotterwerke Geiger near Beilngries, Germany)
	2019–08-24	20:00:00	172,800	48.8461 N	13.7179 E	08:00–15:00	1	20	90	2^15^	GEC2	2c	Cultural Noise (Open House at GERES, 2019–08-25,08:00–15:00 UTC)
	-	-	0.6875	-	-	-	1	100	95	2^9^	-	2d	Horizontal hammer blow recorded on horizontal geophone
*Hydroacoustic*	2005–04-05	22:55:00	180	1.0653 N	97.4227 E	22:20:01	1	120	90	2^9^	H08S1	3a	Mb 4.7 Earthquake Northern Sumatra (Indonesia; Data from USGS)
	2014–06-11	09:00:00	108,000	25.89 S	177.18 W	Ongoing activity	2	120	90	2^15^	H03S1	3b	Submarine Volcanic activity at Monowai, Kermadec Arc, Southwest Pacific Ocean
	2005–05-17	10:54:00	900	-	-	-	1	120	90	2^10^	H08N1	3c	Bioacoustic source (Whale; Indian Ocean)
	2002–04-23	13:15:00	1500	~ 65 S	~ 141 E	-	1	120	90	2^10^	H08S1	3d	Iceberg activity (Tolstoy et al. 2004b; Chapp et al., 2005)
	1996–01-27	22:56:00	1080	22.2224 S	138.7423 W	21:29:57.8	1	120	90	2^10^	PSUR	4a	Nuclear Explosion (French Test near Muroroa, Fangataufa Atoll, South Pacific; yield ~ 120 kt)
	2004–05-05	15:57:00	300	~ 10.14 N	~ 89.07 E	~ 15:28	1	120	90	2^9^	H08S1	4b	In water explosion (Bay of Bengal, unknown source; Bowman et al. 2005b)
	2002–04-28	07:00:00	600	-	-	-	1	120	90	2^10^	H08N1	4c	Airgun
	2005–03-19	20:25:00	300	-	-	-	1	120	90	2^9^	H04N2	4d	Other (probably Submarine Sonar)
*Infrasonic*	2020–04-06	13:40:00	300	~ 47.5 N– ~ 47.8 N	~ 12.6 E– ~ 13.4 E	~ 13:35	1	4	99	2^8^	I26H1	5a	Meteoroid (near Salzburg, Austria)
	2019–07-03	15:30:00	3600	38.79 N	15.21 E	~ 14:46 (first explosion)	0.1	4	90	2^10^	I26H1	5b	Volcanic Eruption (Stromboli volcano, Italy)
	2021–01-30	12:45:00	600	-	-	-	0.1	0.6	99	2^10^	I26H1	5c	Microbaroms (from Atlantic Ocean)
	2008–07-01	19:00:00	1200	-	-	-	0.01	0.1	99	2^11^	I02H1	5d	MAW (Andes)
	2017–09-03	03:50:00	600	41.3 N	129.08 E	03:30:01	0.5	4	90	2^8^	I45H1	6a	Underground Nuclear Explosion (DPRK6—North Korea; yield 30–300 kt)
	2005–12-11	06:01:31	900	51.77 N	0.43 E	07:00:00	0.1	4	90	2^8^	I26H1	6b	Accidental explosion at the Buncefield Oil Depot, UK; estimated yield 51 t
	2020–01-07	03:05:00	3600	28.6 N	80.6 W	02:19:00	0.1	4	90	2^10^	I51H1	6c	Rocket Launch (Falcon 9 from Cape Canaveral)
	2021–07-19	09:25:00	180	~ 49.03 N	~ 12.75 E	~ 09:25	1	5	99	2^7^	I26H1	6d	Supersonic Flight (Nürnberg, Ingolstadt, Regensburg; Bavaria, Germany)
